# Economic models of community-based falls prevention: a systematic review with subsequent commissioning and methodological recommendations

**DOI:** 10.1186/s12913-022-07647-6

**Published:** 2022-03-07

**Authors:** Joseph Kwon, Hazel Squires, Matthew Franklin, Yujin Lee, Tracey Young

**Affiliations:** 1 School of Health and Related Research, University of Sheffield, Regent Court (ScHARR), 30 Regent Street, Sheffield, S1 4DA England; 2 Warwick Medical School, University of Warwick, Gibbet Hill Road, Coventry, CV4 7AL England

## Abstract

**Background:**

Falls impose significant health and economic burdens among older populations, making their prevention a priority. Health economic models can inform whether the falls prevention intervention represents a cost-effective use of resources and/or meet additional objectives such as reducing social inequities of health. This study aims to conduct a systematic review (SR) of community-based falls prevention economic models to: (i) systematically identify such models; (ii) synthesise and critically appraise modelling methods/results; and (iii) formulate methodological and commissioning recommendations.

**Methods:**

The SR followed PRISMA 2021 guideline, covering the period 2003–2020, 12 academic databases and grey literature. A study was included if it: targeted community-dwelling persons aged 60 and over and/or aged 50–59 at high falls risk; evaluated intervention(s) designed to reduce falls or fall-related injuries; against any comparator(s); reported outcomes of economic evaluation; used decision modelling; and had English full text. Extracted data fields were grouped by: (A) model and evaluation overview; (B) falls epidemiology features; (C) falls prevention intervention features; and (D) evaluation methods and outcomes. A checklist for falls prevention economic evaluations was used to assess reporting/methodological quality. Extracted fields were narratively synthesised and critically appraised to inform methodological and commissioning recommendations. The SR protocol is registered in the Prospective Register of Systematic Reviews (CRD42021232147).

**Results:**

Forty-six models were identified. The most prevalent issue according to the checklist was non-incorporation of all-cause care costs. Based on general population, lifetime models conducting cost-utility analyses, seven interventions produced favourable ICERs relative to no intervention under the cost-effectiveness threshold of US$41,900 (£30,000) per QALY gained; of these, results for (1) combined multifactorial and environmental intervention, (2) physical activity promotion for women, and (3) targeted vitamin D supplementation were from validated models. Decision-makers should explore the transferability and reaches of interventions in their local settings. There was some evidence that exercise and home modification exacerbate existing social inequities of health. Sixteen methodological recommendations were formulated.

**Conclusion:**

There is significant methodological heterogeneity across falls prevention models. This SR’s appraisals of modelling methods should facilitate the conceptualisation of future falls prevention models. Its synthesis of evaluation outcomes, though limited to published evidence, could inform commissioning.

**Supplementary Information:**

The online version contains supplementary material available at 10.1186/s12913-022-07647-6.

## Background

Population ageing is projected to increase the prevalence of chronic diseases and frailty around the world and their associated clinical conditions including falls [1–3]. Around a third of people aged 65 and over (65+) fall each year [4]. Falls impose significant morbidity and mortality burdens on older people [5], including fear of falling [6–8], depression [9], functional decline and dependence [10–12], and fatality [13–15]. They also impose high costs on the health and social care systems [16–18], and on society through informal caregiver burden and loss of older person’s social contribution [19, 20].

Trial-based evidence consistently suggests that diverse types of falls prevention interventions in the community setting can significantly reduce the number of falls and fallers [21–23]. In England and Wales, the National Institute for Health and Care Excellence (NICE) falls prevention clinical guideline (CG161) recommends that older persons aged 65+ in the community (i.e., not in extended or institutionalised care settings such as nursing homes and hospital wards) are routinely screened for falls risk by health and social care professionals [4]. High-risk individuals should subsequently be referred to multifactorial intervention involving multidisciplinary falls risk assessment followed by tailored treatments including exercise, home assessment and modification (HAM), vision correction and medication change [4]. In addition to this proactive (i.e., initiated by professional referral) pathway, CG161 also recommends a reactive pathway for those admitted to a medical facility for a fall (multifactorial intervention and HAM) [4]. Older persons may also ‘self-refer’ by voluntarily enrolling in a falls prevention intervention (e.g., exercise) available in the community [24, 25].

Given scarce care resources, commissioning of falls prevention should be informed by economic evaluations that consider the costs and consequences of any falls prevention strategy against the next best alternative use of resources [26]. Decision modelling is a vehicle for economic evaluation that combines multiple epidemiological, intervention and economic parameters from diverse sources in a coherent mathematical and statistical framework suitable for decision-making [27]. Relative to economic evaluations alongside a single clinical study, models can inform decisions at a broader population level (rather than for specific patient groups), incorporate the long-term costs and consequences of falls, and systematically evaluate the impact of all relevant scenarios and input parameter uncertainties as commissioning relevant factors for consideration [28].

A systematic review uses systematic and explicit methods to identify, select and critically appraise relevant research in the topic area, and perform data extraction and analyses [29, 30]. Conducting a systematic review of community-based falls-prevention decision models can perform two functions simultaneously. First to inform commissioning decisions, by summarising all available model outcomes relevant to the decision problem and context; alternatively, it can identify an existing model that can be adapted and re-used [31]. Second to appraise the methodological features of models, detailing and critically appraising methodological features that significantly affect the evaluation results including structural assumptions made by decision models [26, 31]; this can be achieved by applying a pre-established methodological and reporting quality checklist, then conducting a narrative synthesis of the methodological features including their strengths and limitations [32]. Ideally, the systematic review should perform both functions together: the commissioners would benefit from the methodological appraisal that qualifies the model outcomes; the modellers basing the conceptualisation of future models on the reviewed methodological features would need to know how the features affect the model outcomes and therefore the commissioning strategy.

A prior systematic overview of systematic reviews of falls prevention economic evaluations assessed how well previous reviews had performed both functions [33]. Seven systematic reviews covering 21 decision models were identified [34–40]. The systematic overview reported that the identified systematic reviews extracted a limited range of methodological model features and evaluation outcomes to inform commissioning; for example, the extracted methodological features were limited to model type and brief summaries of data sources. A pilot Medline search by the current authors identified 10 decision models of community-based falls prevention that were not included in the aforementioned seven systematic reviews. Therefore, current systematic reviews are now outdated and provide insufficient detail.

The aim of this study is to conduct a systematic review of community-based falls prevention economic models. We systematically search for and identify community-based falls prevention decision models, then apply a pre-established checklist for assessing the reporting and methodological quality of falls prevention economic evaluations [32]. We subsequently conduct a narrative synthesis and critical appraisal of methodological features of identified models including key features of falls epidemiology, falls prevention interventions, and evaluation methods. We then formulate methodological and commissioning recommendations based on the aforementioned. This systematic review can inform commissioners and other consumers of economic evidence (e.g., care professionals and patient groups), producers of economic evidence (e.g., modellers) and systematic reviewers interested in the review methodology.

## Methods

The systematic review protocol is registered on the Prospective Register of Systematic Reviews (CRD42021232147). We followed the Preferred Reporting Items for Systematic Reviews and Meta-Analyses (PRISMA) guideline and the checklist is reported in the Supplementary Materials [29, 30].

### Data sources and study selection

The search covered the period January 2003 to December 2020 and 12 academic databases: Medline, Embase, PubMed, CDSR, CENTRAL, EconLit, CINAHL, PsycInfo, ASSIA, CRD, CEA Registry and PEDro. Grey literature was searched from online sites of the Department of Health, Chartered Society of Physiotherapy, College of Occupational Therapy, Royal College of Nursing and Age UK. A previous systematic review to inform the NICE falls prevention clinical guideline had covered the period before 2003 and found just one decision model [34]; hence, the period from 2003 was covered. The search strategy was an intersection between terms for falls, older people, and economic evaluation. All database and grey literature search strategies are given in Tables A1.1 to A1.8 and related text in Supplementary Materials. References and citations of included studies were also searched.

Two researchers (JK and YL) independently reviewed the titles and abstracts of identified articles at the first stage and the full texts of approved articles at the second stage. Those that received two second-stage approvals were included for data extraction. Another researcher (TY) arbitrated in case of disagreement.

A study was included if it: (i) targets a population of community-dwelling (i.e., not in extended or institutionalised care settings such as nursing homes and hospital wards) older persons (aged 60+) and/or individuals aged 50–59 at high falls risk; (ii) evaluates intervention(s) designed to reduce the number of falls or fall-related injuries; (iii) against any comparator(s); (iv) reports outcomes of economic evaluation (i.e., comparative analysis of interventions in terms of their relative costs and consequences [26]); (v) uses a decision model [26]; and (vi) has English full text. The age range in criterion (i) sought to increase the evidence for primary and/or earlier-life prevention which is a key principle of geriatric public health intervention [41, 42]. The Cochrane systematic reviews of community-based falls prevention randomised controlled trial (RCT) evidence had also set the lower age bound at 60 rather than 65 [21–23]; a previous systematic review of community-based falls prevention economic evaluations had covered the high-risk group aged 50–64 [37].

Models evaluating interventions for specific disease areas (e.g., stroke) with minor falls prevention components were excluded. Interventions aiming to reduce specific falls risk factor (e.g., balance) and/or health consequences of falls (e.g., fear of falling) were excluded if the model did not explicitly incorporate falls as events. Economic evaluations alongside a single clinical study were excluded but their references were searched. Eligible models included in previous systematic reviews of falls prevention economic evaluations were included [34–40].

### Data extraction and synthesis

Table 1 shows the data fields extracted from identified models, including the following categories: (A) model and evaluation overview; (B) falls epidemiology features; (C) falls prevention intervention features; (D) evaluation methods and outcomes; and (E) key methodological challenges for public health economic models. The data extraction was primarily conducted by JK, supported by YL.

**Table 1:** Data fields extracted from decision models identified by systematic review

Category	Data field
Reporting and methodological quality checklist	The checklist designed for falls prevention economic evaluations by a panel of falls prevention experts [32] was adapted to specifically suit decision models. There were 32 items, each scored 0 (recommendation not followed), 0.5 (partially followed), and 1 (fully followed), giving maximum score of 32. See Table A2 in Supplementary Materials for adapted version.
(A) Model and evaluation overview	1. Bibliography: author(s); publication year 2. Setting and aim: country; region; decision-maker; evaluation aim 3. Target population demographics and comorbidities (e.g., residence,^a^ age, sex, socioeconomic status, health conditions unrelated to falls risk) 4. Type of analysis: e.g., CEA; CUA; CBA; ROI^b^ 5. Perspective (e.g., public sector, societal) 6. Cost-effectiveness threshold: monetary amount and type (e.g., health opportunity cost in healthcare system, willingness to pay as consumer) 7. Model type (e.g., decision tree, Markov) 8. Model time horizon 9. Discount rates (if time horizon longer than 1 year) 10. Model cycle length (if any)
(B) Falls epidemiology features	1. Characterising baseline falls risk of target population 2. Characterising multiple falls per year (recurrent falls) 3. Risk factors for falls 4. Health consequences of falls: fall/injury type; long-term health consequences (e.g., institutionalisation, excess mortality risk) 5. Health utility data: fall-related loss; comorbidity status 6. Economic consequences of falls: care resource types; unit costs; all-cause/comorbidity care costs^c^
(C) Falls prevention intervention features	1. Intervention characteristics: type;^d^ comparator(s); component; access pathway^e^ 2. Falls risk screening method^f^ 3. Intervention resource use and costs: auxiliary implementation resources (e.g., marketing to improve uptake); therapeutic resources (e.g., staff labour). 4. Intervention efficacy: metric;^g^ fall type;^h^ effectiveness period^i^ 5. Wider health effects of interventions beyond falls prevention^j^
(D) Evaluation methods and results	1. Model validity: structural/face;^k^ internal; external; cross^l^ 2. Assessing parameter uncertainty: DSA; PSA 3. Scenario analyses: to assess impact of structural assumptions on outcomes. 4. Aggregate health and cost outcomes (e.g., total intervention cost, total QALY gain, total number of falls prevented) 5. Cost-per-unit ratios (e.g., incremental cost per QALY gain) 6. Wider decisional outcomes (e.g., reduction in social inequities of health) 7. Currency: original type/year; conversion to same currency for comparison 8. Discussion by evaluation authors: generalisability; policy implementation; model strengths and limitations
(E) Key methodological challenges for public health economic model	1. Capturing non-health outcomes and societal intervention costs 2. Considering heterogeneity and dynamic complexity: e.g., long-term progression of falls risk factors/profile 3. Considering theories of human behaviour and implementation: e.g., implementation quality (i.e., uptake and adherence rates) 4. Considering social determinants of health and conducting equity analyses

*Abbreviations*: *CBA* Cost-benefit analysis, *CEA* Cost-effectiveness analysis, *CUA* Cost-utility analysis, *DSA* Deterministic sensitivity analysis, *PSA* Probabilistic sensitivity analysis, *QALY* Quality-adjusted life year, *RCT* Randomised controlled trial, *ROI* Return on investment

^a^Community-dwelling or institutionalised

^b^Cost-effectiveness analysis (CEA) uses natural health units (e.g., number of falls) as health outcomes; cost-utility analysis (CUA) generic quality-adjusted life year (QALY). Cost-benefit analysis (CBA) values health outcomes using societal or consumption value of health. Return on investment analysis (ROI) only compares the net financial outcomes of two or more interventions

^c^Expert guideline on falls prevention economic evaluation recommends that evaluations report all-cause healthcare costs in the base case and fall-related costs in sensitivity analysis [32]. All-cause care costs are comprised of fall-related and comorbidity care costs

^d^Intervention type classification should follow the Prevention of Falls Network Europe categories [43]

^e^Potential intervention pathways are: proactive – initiated by professional screening/referral; reactive – initiated after medical attention for a fall; and self-referred – enrolled voluntarily by older persons

^f^Falls risk screening is required if: (1) model prescribes intervention to a subset of the whole target population with certain characteristics (e.g., higher falls risk) and this subset must be identified; and (2) model’s target population itself is a specific patient group (e.g., cataract patients) and this group must be identified from the general population before model baseline. Falls risk screening is distinct from falls risk assessment as part of multifactorial intervention

^g^This concerns models that import falls efficacy evidence from external intervention studies. Main falls incidence metrics are falls risk and falls rate, and their matching efficacy metrics are relative risk (RR) and rate ratio (RaR), respectively. Models should ensure that the external efficacy metric matches the internal falls incidence metric

^h^Like note f, this concerns decision models using external efficacy evidence. The fall type (e.g., hospitalised fall, fall-induced fracture) for the efficacy data should match that for the model incidence

^i^The effectiveness period is a function of efficacy durability and implementation sustainability. Efficacy durability should not extend beyond the intervention study’s timespan unless the intervention is sustained [32]. Key determinants of sustainability are demand-side persistence and supply-side maintenance

^j^For example, falls prevention exercise can improve cardiovascular health [25]

^k^Structural or face validity concerns validity of model structure, data sources and assumptions as assessed by modelling and disease-area experts and broader stakeholders [31, 44]. Structural validity can be assessed prospectively during the model development stage through proactive involvement of stakeholders in model conceptualisation; it can also be assessed retrospectively by evaluating scenarios on different structural assumptions [31]

^l^Internal validity concerns the accuracy of model coding; external validity concerns comparability between model and real-world results; and cross validity concerns comparability between model results and results of other models addressing the same decision problem [44]

#### Model overview and checklist scores for reporting and methodological quality

The extracted features for model and evaluation overview under category (A) in Table 1 were reported. A checklist specifically designed to assess the reporting and methodological quality of falls prevention economic evaluations was applied after being adapted for use on decision models, as described and presented in Supplementary Materials, Table A2 [32].

#### Narrative synthesis of methodological features and methodological recommendations

The extracted methodological features under categories (B), (C) and (D) in Table 1 were narratively synthesised, mainly using tabular formats. The synthesised features were selected based on their potential to affect model credibility and evaluation results as noted by guidelines on conducting and reporting falls prevention economic evaluation [32], wider falls prevention literature including the NICE clinical guideline CG161 [4], and the health technology assessment (HTA) checklist for quality assessment of decision models [45]. Critical appraisal identified between-study variation in the methods used to characterise the features and their respective strengths and limitations (including those mentioned by the model’s developers). Methodological recommendations for future model development were subsequently formulated by this systematic review.

Features under category (E) were informed by the systematic methodological review of key methodological challenges to public health economic modelling [46]; these features are synthesised and appraised (with associated methodological recommendations) in a future publication. Nevertheless, features that potentially affected the model outcomes significantly are discussed in this article whilst formulating commissioning recommendations.

#### Developing commissioning recommendations by this systematic review

Extracted under category (D) in Table 1, commissioning recommendations from model evaluation results are based primarily on a subset of models that targeted general older populations – as opposed to specific patient groups – and conducted analyses over a lifetime horizon. Prioritising this subset addresses the information needs of decision-makers overseeing geographically defined jurisdictions (e.g., national) [28]. The evaluation over a lifetime horizon is recommended by the expert guideline on falls prevention economic evaluation [32].

The recommendations considered all available evaluation outcomes – including not only cost-per-unit ratios but also aggregate, population-level impact and wider decisional outcomes (e.g., impact on social inequities of health) – and methodological caveats potentially affecting credibility and outcomes. Monetary outcomes were converted to US$ in 2021 using the consumer price index (CPI) in the country of study to account for inflation up to 2021 [47] and the most recent purchasing power parity (PPP) exchange ratio between US$ and the original currency [48]. For cost-utility analysis (CUA), an ICER less than US$41,900 (£30,000) per quality-adjusted life year (QALY) gain was deemed cost-effective according to the threshold recommended by the NICE HTA guideline [49].

## Results

### Search results

**Fig. 1:**
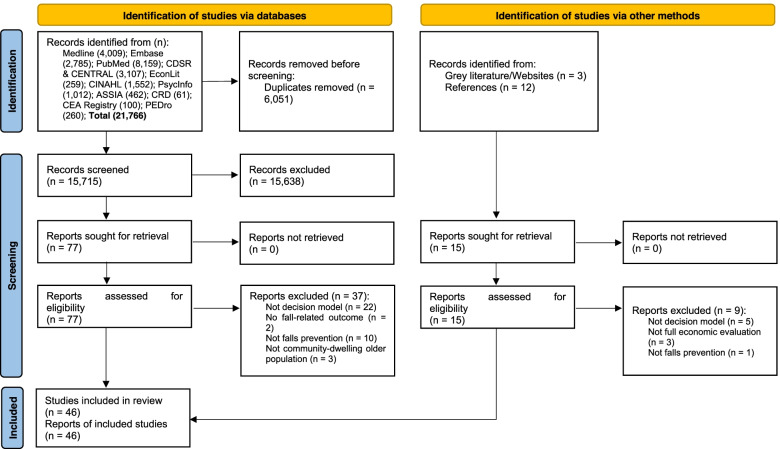
Preferred Reporting Items for Systematic Reviews and Meta-Analyses flow diagram

Figure 1 presents the PRISMA flow diagram. In total, 15,730 titles and abstracts were screened. Ninety-two full texts were screened from which 46 decision models were identified. Six studies were identified from the grey literature and references of other studies. The main reason for exclusion at the full text screening stage was not conducting economic evaluation via decision modelling. The titles of the excluded studies are given in Table A3 in the Supplementary Materials.

### Overview of included decision models

Table 2 provides an overview of the 46 included models. Apart from Agartioglu [50] set in Turkey, all models were set in developed countries: 14 from the US and Canada (30.4%); 12 Australia and New Zealand (26.1%); 11 UK (23.9%); and eight Europe (17.4%). Twenty-four (52.2%) models aimed to inform decision-making at the national level, while the rest adopted more local application levels including state, city, and clinical commissioning groups in the UK.

**Table 2:** Overview and quality score of included falls prevention decision models

#	Reference	Setting	Target population	Type of analysis	Perspective	Intervention type	Comparator	Model type	Time horizon
1	Agartioglu (2020) [50]	Turkey, Izmir	CD adults aged 65+	CEA	Public sector	HAM	UC	DT	1 year
2	Albert (2016) [51]	US, Pennsylvania	CD adults aged 50+ (mean age 75.5)	CUA	Public sector	MF int.	UC	DT	1 year
3	Alhambra-Borras (2019) [52]	Spain, Valencia, hospital level	CD adults aged 65+ at high falls risk or frail with no severe physical or cognitive limitation	CUA	Public sector	Exercise	UC	Markov cohort^a^	Lifetime
4	Beard (2006) [53]	Australia, NSW	CD adults aged 60+	CBA; ROI	Public sector; Societal	MC (intersectoral) int.^b^	UC	Binary decision^c^	5 years
5	Boyd (2020) [54]	New Zealand	Adults aged 65+	CUA	Public sector	Cataract surgery (expedited, routine)	NR	Markov cohort	Lifetime
6	Carande-Kulis (2015) [55]	US, private insurers	CD adults aged 65+	ROI	US health insurance payer	Exercise (2 forms); MC int. (Stepping On)	NR	Binary decision	1 year
7	CSP (2016) [56]	UK, varying regions	CD adults aged 65+	ROI	Public sector	FRS + Exercise (physiotherapy)	NR	DT	1 year
8	Church (2011) [57]	Australia, NSW	CD adults aged 65+ (separate model for residential care)	CEA; CUA	Public sector	Exercise (3 forms); MC int.; MF int.; MRA; Exp. cataract surgery; Med. modification; Cardiac pacing	NR	Markov cohort	10 years
9	Church (2012) [58]	Australia, NSW	CD adults aged 65+	CEA; CUA	Public sector	Exercise (4 forms); MC int.; MF int. (2 forms); MRA; HAM; Exp. cataract surgery; Cardiac pacing; Med. modification	NR; Cross-comparison	Markov cohort	Lifetime
10	Comans (2009) [59]	Australia, Brisbane	CD adults aged 65+, falls history in past 6 months or gait/functional decline and cognitively intact	ROI	Societal	MF int. (2 forms)	NR	Binary decision	1 year
11	Day (2009) [60]	Australia, varying regions	CD adults aged 50+ (age and characteristics differ by intervention type)^d^	CEA	Public sector; Societal	Exercise (2 forms); HAM; MF int.; Med. modification; Cardiac pacing	NR	DT	1 year
12	Day (2010) [61]	Australia	CD adults aged 70+	CEA	Public sector; Societal	Exercise (Tai Chi)	NR	DT	1 year
13	Deverall (2018) [62]	New Zealand	CD adults aged 65+	CUA	Public sector; Societal	Exercise (3 forms)	NR	Markov cohort	25 years
14	Eldridge (2005) [63]	UK, primary care trust	Adults aged 65+ in community or nursing home	CUA	Public sector	FRS + MF int. or Exercise	UC	DT + Markov cohort	Lifetime
15	Farag (2015) [64]	Australia	CD adults aged 65+ without falls history	CUA	Public sector	Non-specific intervention	NR	Markov cohort	Lifetime
16	Franklin (2019) [65]	UK, city level	CD adults aged 65+	CUA	Public sector (2 types)	FRS + Exercise (3 forms) or HAM	NR; Cross-comparison	DT + Markov cohort	2 years
17	Frick (2010) [66]	US	CD adults aged 65+	CUA	US healthcare payer^e^	Exercise (2 forms); HAM; MF int. (2 forms); Vit. D; Med. modification	Cross-comparison	Binary decision	1 year^f^
18	Hektoen (2009) [67]	Norway	CD women aged 80+	CEA	Societal	Exercise	NR	Binary decision	1 year
19	Hiligsmann (2014) [68]	Belgium	Adults aged 60+ with osteoporosis	CUA	Societal	Vit. D and calcium	NR	Markov patient^a^	Lifetime
20	Hirst (2016) [69]	UK	Women aged 75+ on chronic pain medication	CUA	Public sector	Med. modification (Transdermal Buprenorphine)	Tramadol	DT^g^	1 year
21	Honkanen (2006) [70]	US, Medicare/aid	Adults aged 65+ living in community at baseline	CUA; ROI	Societal	Hip protectors	NR	Markov cohort	Lifetime
22	Howland (2015) [71]	US, Massachusetts	CD adults aged 65+ admitted to A&E due to fall	ROI	US healthcare payer^e^	MC int. (MoB/VLL)	NR	Binary decision	1 year
23	Ippoliti (2018) [72]	Italy, Piedmont	CD adults aged 65+ living in mountainous areas	ROI	Public sector	MF int.	NR	Binary decision	3 years
24	Johansson (2008) [73]	Sweden, Stockholm	CD adults aged 65+	CUA	Societal	MC (intersectoral) int.^h^	UC	Markov cohort	Lifetime
25	Lee (2013) [74]	US, Medicare/aid	CD adults aged 65–80 without falls history	CBA	Public sector	Vit. D (targeted, universal)	NR	DT + Markov cohort	3 years
26	Ling (2008) [75]	US, Hawaii	CD adults aged 65+ with falls history or other risk factors	ROI	US healthcare payer^e^	HAM	NR	Binary decision	1 year
27	McLean (2015) [76]	Australia, Melbourne	CD adults aged 70+	CEA; CUA	Public sector	Exercise	UC	DT	18 months
28	Miller (2011) [77]	US, Texas	CD adults aged 50+ at high falls risk	ROI	US healthcare;^e^ Societal	MC int. (MoB/VLL)	NR	Binary decision	2 years
29	Mori (2017) [78]	US	CD women aged 65+ at osteoporosis risk without previous fracture	CUA	Societal	Exercise and bisphosphonate combined	Cross-comparison: single or no intervention	DT + Markov patient	Lifetime
30	Moriarty (2019) [79]	Ireland	CD adults aged 65, no current/previous adverse events for benzodiazepine/PPI	CUA	Public sector	Med. modification (Benzodiazepine, PPIn)	Inappropriate prescribing	DT + Markov patient	35 years
31	Nshimyu-mukiza (2013) [80]	Canada	Women aged 40+ (with subgroup aged 65+)	CEA; CUA	Public sector	Fracture risk screening + Physical activity, Vit. D and calcium, and/or Osteoporosis screen & treat	NR; Cross-comparison	DT + Markov patient	Lifetime
32	OMAS (2008) [81]	Canada, Ontario	CD adults aged 65+	CEA; ROI	Public sector	Exercise; HAM; Vit. D and calcium; Med. modification; gait-stabilizer	NR	Markov cohort	Lifetime
33	Pega (2016) [82]	New Zealand	CD adults aged 65+	CUA	Public sector	HAM	NR	Markov cohort	Lifetime
34	Poole (2014) [83]	UK	Adults aged 65+	ROI	Public sector	Vit. D	NR	Binary decision	1 year
35	Poole (2015) [84]	UK	CD adults aged 60+	CUA; ROI	Public sector	Vit. D	NR	Markov cohort	5 years
36	PHE (2018) [85]	England, varying regions	CD adults aged 65+	CUA; ROI	Public sector	Exercise (3 forms); HAM	NR	DT	2 years
37	RCN (2005) [34]	England & Wales	CD adults aged 60+	CUA	Public sector	Exercise; MF int.	NR	Markov cohort	Lifetime
38	Sach (2007) [86]	UK	Women aged 70+ with bilateral cataracts	CEA; CUA	Public sector; Societal	Exp. cataract surgery (first eye)	UC (routine surgery)	Binary decision	Lifetime extrapol.^i^
39	Sach (2010) [87]	UK	Women aged 70+ with second operable cataract	CUA	Public sector; Societal	Exp. cataract surgery (second eye)	UC (no surgery)	Binary decision	Lifetime extrapol.^i^
40	Smith (2016) [88]	UK, NW London	Adults aged 65+ covered by GP practice and hospital	ROI	Public sector	FRS + MF int.	Cross-comparison	Risk prediction	1 year
41	Tannenbaum (2015) [89]	US, Medicare/aid	CD adults aged 65+ with insomnia	CUA	Public sector	Med. modification; CBT	NR; Cross-comparison	Markov cohort	1 year
42	Turner (2020) [90]	Canada, Quebec	CD adults aged 65+ who are chronic users of sedatives for insomnia	CUA	Public sector	Med. modification	NR	DT + Markov cohort	1 year
43	Velde (2008) [91]	Netherlands	CD geriatric outpatient population with falls history (mean age 78)	CEA	Public sector	Med. modification	NR	Binary decision	1 year^f^
44	Wilson (2017) [92]	New Zealand, Manukau	CD adults aged 65+	CUA	Public sector	HAM	NR	Markov cohort	Lifetime
45	Wu (2010) [93]	US, Medicare/aid	CD Medicare beneficiaries aged 65+ with falls history	CEA; ROI	Public sector; Societal	MF int.	NR	Binary decision	1 year
46	Zarca (2014) [94]	France	Adults aged 65+ without previous hip fracture	CEA; CUA	Public sector	Vit. D (targeted (2), universal)	NR; Cross-comparison	DT + Markov patient	Lifetime

*Abbreviations*: *CBA* Cost-benefit analysis, *CBT* Cognitive behavioural therapy, *CD* Community-dwelling, *CEA* Cost-effectiveness analysis, *CSP* Chartered Society of Physiotherapy, *CUA* Cost-utility analysis, *DT* Decision tree, *Exp.* Expedited, *Extrapol.* Extrapolated, *FRS* Falls risk screening, *HAM* Home assessment and modification, *Int.* Intervention, *MC* Multiple-component, *Med.* Medication, *MF* Multifactorial, *MoB/VLL* Matter of Balance Lay-Led Version, *MRA* Multifactorial risk assessment only, *NR* Non-receipt of modelled intervention(s), *NSW* New South Wales, *OMAS* Ontario Medical Advisory Secretariat, *PHE* Public Health England, *PPIn* Proton pump inhibitor, *RCN* Royal College of Nursing, *ROI* Return on investment analysis, *UC* Usual care

^a^“Markov cohort” describes cohort-level Markov models that simulate the proportion of a population that experience an event (e.g., fall incidence) and progresses to a different model state. “Markov patient” describes patient- or individual-level Markov models that simulate the progression of individuals with unique set of characteristics [95]

^b^Intervention included individually tailored education, HAM and exercise and public space safety improvement

^c^Binary decision models include two scenarios, with or without intervention, and no time-based cycles or probability trees

^d^Cardiac pacing targeted population aged 50+ due to their high falls risk. Other interventions targeted populations aged 65+

^e^This would include Medicare/aid, private health insurance and patients

^f^One-year horizon with lifetime costs and health effects of falls

^g^Authors described the model as microsimulation; but there was only a single one-year cycle. Hence, the model is classified as a decision tree

^h^Intervention included individually tailored education, group balance exercises, Tai Chi, other physical activities and HAM, neighbourhood hazard removal and housing reconstruction

^i^One-year trial outcomes are extrapolated over lifetime horizon

Most models (*n* = 25; 52.2%) targeted a general population of community-dwelling adults aged 60+ or 65+; two targeted women only [67, 80]. Two models targeted general adult populations aged 65+ which would contain a minority of institutionalised adults [54, 88]; two incorporated institutionalisation as a non-final model state [63, 70]. Five targeted populations with falls history [59, 71, 75, 91, 93]; two populations at high falls risk without specifying cause [52, 77]. Eight targeted specific patient populations: osteoporosis or high osteoporosis risk [68, 78]; fall-risk-increasing drug (FRID) users [69, 79, 89, 90]; and cataracts [86, 87]. Nshimyumukiza [80] incorporated incoming cohorts, newly entering each year for 10 years.

There were four types of economic analysis: cost-effectiveness analysis (CEA), cost-benefit analysis, (CBA), return-on-investment analysis (ROI), and CUA. No further types, e.g., cost-consequence analysis (CCA), were identified. There were two costing perspectives: public sector and societal. Several models adopted multiple types of analysis and perspectives, resulting in 69 distinct analyses. Of these, CUA was most used (*n* = 32; 46.4%), followed by ROI and CEA (each *n* = 17; 24.6%), and then CBA (*n* = 3; 4.3%). Around a third of analyses (*n* = 22) adopted the societal perspective.

Exercise was the most evaluated intervention type with 17 models; eight evaluated multiple exercise forms. Multifactorial intervention was the second most evaluated type with 13 models: three evaluated multiple forms [58, 59, 66]; two combined multifactorial intervention with environmental modifications [53, 73]. Twelve evaluated multiple types of interventions: four compared multiple types directly [58, 66, 80, 89]. The most common comparator scenario was not receiving the modelled intervention(s). Eight models described the ‘usual care’ (without falls prevention properties) received in the comparator scenario, e.g., non-expedited cataract surgery compared to expedited [86]; but others (24 of 32 with non-receipt scenario) were vague in the description or used ‘no intervention’ and ‘usual care’ interchangeably [34, 60, 61, 85, 93].

There were four model type categories: (1) binary decision (*n* = 14); (2) static (*n* = 9); (3) cohort-level Markov (*n* = 19); and (4) patient- or individual-level Markov (*n* = 4). Binary decision models compared the state of the world with and without the intervention and did not incorporate transition probabilities or time cycles. All static models except Smith [88] were decision trees without time cycles; Smith [88] compared several falls risk cut-off levels without time cycles. Model time horizon varied between one year and lifetime. Seventeen of 23 Markov models adopted lifetime horizons.

### Checklist scores for methodological and reporting quality

Tables 3, 4 and 5 shows the item-specific checklist scores for models. The overall quality score ranged between 13.5 and 27 (average 21.2) of maximum 32. The lowest scored item across models was item 15, which recommends reporting total/all-cause health resource utilisation costs under base case analysis and fall-related costs under sensitivity analysis. For this, only four models (all using primary collection of cost data) incorporated all-cause healthcare costs as the main economic outcome [51, 52, 86, 87]; six incorporated comorbidity care costs, which together with fall-related costs constitute all-cause costs [54, 62, 70, 73, 82, 92]. The second lowest scored item was item 21, which recommends: (i) reporting intervention costs and all-cause/fall-related healthcare costs separately; and (ii) reporting both aggregate and mean costs. For this, eight followed both recommendations [59, 67, 69, 71–73, 85, 93], five followed (i) only [56, 75, 76, 83, 84], and four followed (ii) only [64, 80, 81, 94]. The third lowest scored item was item 8 for clearly stating and justifying the comparator which, as discussed above, was done by less than half (*n* = 22) of studies.

**Table 3:**
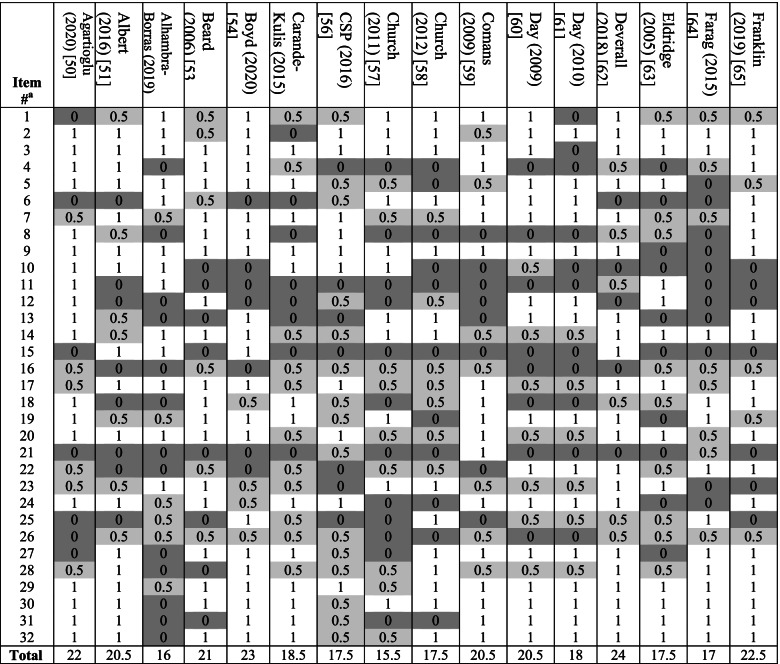
Results of methodological and reporting quality checklist application to included models

^a^See Table A2 in Supplementary Materials for item contents. Study is given a score of 1 if deemed to have followed the item recommendation fully, 0.5 if partially (light grey shading) and 0 (dark grey shading) if not followed

**Table 4:**
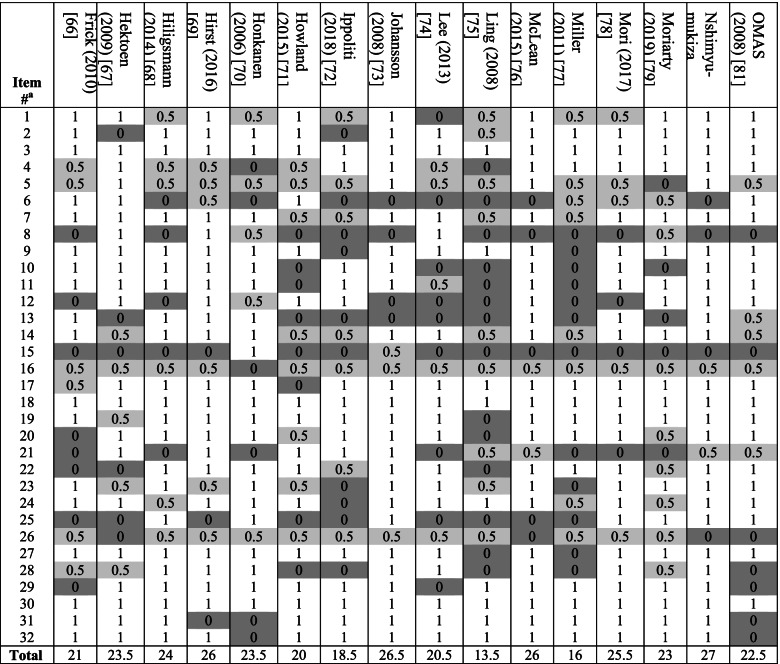
Results of checklist application to included studies

^a^See Table A2 in Supplementary Materials for item contents. Study is given a score of 1 if deemed to have followed the item recommendation fully, 0.5 if partially (light grey shading) and 0 (dark grey shading) if not followed

**Table 5:**
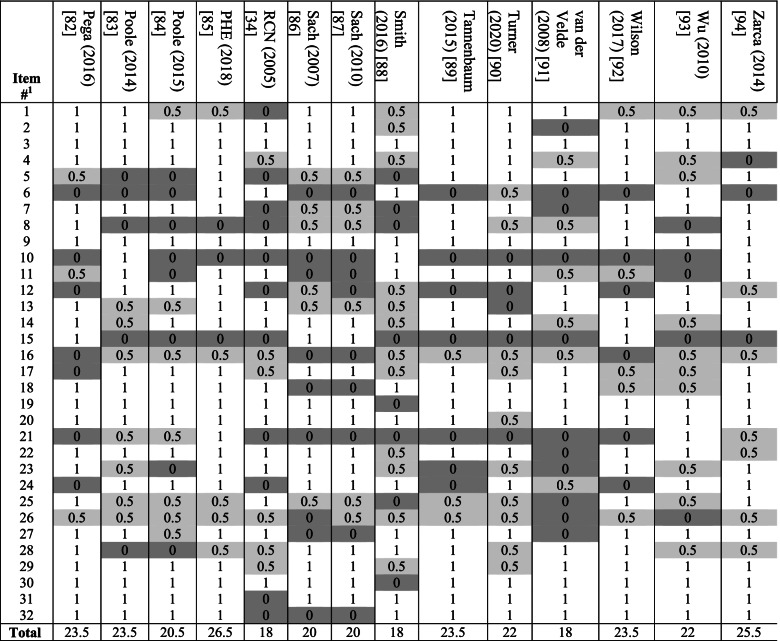
Results of checklist application to included studies (*n* = 46)

^a^See Table A2 in Supplementary Materials for item contents. Study is given a score of 1 if deemed to have followed the item recommendation fully, 0.5 if partially (light grey shading) and 0 (dark grey shading) if not followed

### Narrative synthesis: falls epidemiology features

As detailed in Table 1, falls epidemiology features are synthesised based on: (1) characterising baseline falls risk; (2) characterising recurrent falls; (3) range of falls risk factors; (4) range of falls health consequences; (5) health utilities for CUA; and (6) range of fall-related economic consequences.

#### Baseline falls risk

Table 6 shows four main approaches for characterising the baseline falls risk/rate of models: (1) analysis of individual-level epidemiological data; (2) use of published epidemiological data or expert/author opinion; (3) use of internal intervention study; and (4) use of falls risk/rate from RCT control group.

**Table 6:** Evidence sources for baseline falls risk/rate used by falls prevention decision models

Data source	N	Study label (***n*** = 46)^**a**^
(1) Individual-level epidemiological data	8	BODE3 models – Boyd (2020) [54], Deverall (2018) [62], Pega (2016) [82], Wilson (2017) [92]; Eldridge (2005) [63]; Ippoliti (2018) [72]; OMAS (2008) [81]; Smith (2016) [88]
(2) Published epidemiological data or expert/author opinion	25	Agartioglu (2020) [50]; Carande-Kulis (2015) [55]; CSP (2016) [56]; Church (2011) [57]; Church (2012) [58]; Farag (2015) [64]; Franklin (2019) [65]; Frick (2010) [66]; Hiligsmann (2014) [68]; Hirst (2016) [69]; Honkanen (2006) [70]; Howland (2015) [71]; Lee (2013) [74]; Ling (2008) [75]; Miller (2011) [77]; Mori (2017) [78]; Moriarty (2019) [79]; Nshimyumukiza (2013) [80]; Poole (2014) [83]; Poole (2015) [84]; RCN (2005) [34]; Tannenbaum (2015) [89]; Turner (2020) [90]; Wu (2010) [93]; Zarca (2014) [94]
(3) Internal intervention study^b^ evidence	9	Albert (2016) [51]; Alhambra-Borras (2019) [52]; Beard (2006) [53]; Comans (2009) [59]; Johansson (2008) [73]; McLean (2015) [76]; Sach (2007) [86]; Sach (2010) [87]; Velde (2008) [91]
(4) Risk/rate from external RCT control group	4	Day (2009) [60]; Day (2010) [61]; Hektoen (2009) [67]; PHE (2018) [85]

*Abbreviations*: *BODE3* Burden of Disease Epidemiology, Equity and Cost-Effectiveness, *CSP* Chartered Society of Physiotherapy, *OMAS* Ontario Medical Advisory Secretariat, *PHE* Public Health England, *RCN* Royal College of Nursing, *RCT* Randomised controlled trial

^a^See Table 2 for study references; parenthesised number refers to the number of models included in the table

^b^This may be randomised or non-randomised

Eight models employing (1) estimated the baseline falls risk/rate by analysing individual-level data relevant to the decision-making context. One used a local survey [63], but the other seven analysed administrative healthcare (‘routine’) datasets. For example, the four BODE3 models developed by the same research group analysed the insurance claims data at national and state levels to estimate the incidence rates of falls requiring medical attention (i.e., MA falls). A key strength of routine data is that falls incidence is linked to consequent care utilisation and cost; the latter can then be stratified by individual-level risk factors. The routine data should contain individual identifiers to distinguish between number of fallers and falls per faller. The BODE3 models did not make this distinction, counting multiple falls per person as multiple fallers and overestimating the baseline falls risk.

Twenty-five models used published epidemiological evidence (*n* = 22) or expert opinion (*n* = 3) [57, 58, 77]. Compared to approach (1), the use of published evidence restricted the range of falls risk factors and relevant population subgroups (see below). Nevertheless, published evidence allowed parameterisation of fall-related events that are not well-observed in routine data (e.g., non-MA falls).

Nine models sourced the baseline falls risk/rate and intervention effectiveness from the same internal intervention study. For example, Albert [51] developed a decision tree model using the baseline risk, effectiveness, and costs evidence from a quasi-experimental evaluation of multifactorial intervention. The reliance on a single intervention study makes these models similar to non-modelling evaluations alongside clinical studies. Nevertheless, the nine models: explicitly developed models using internal data [51, 52, 76]; extrapolated results over a longer time horizon [73, 86, 87]; extrapolated results to national population [91]; and extrapolated results to a wider societal perspective [53]. These models assumed that the internal intervention sample is representative of the target population; this assumption would not hold if there were sampling biases.

Four models used the falls risk/rate from the control group of an external RCT (or pool of RCTs). For example, Day [61] used the falls rate pooled from two Tai Chi RCTs to characterise the baseline rate, then applied the Tai Chi efficacy from a separate meta-analysis. Analysts can draw on diverse external RCTs to characterise the baseline risk; heterogeneous risks across subpopulations can be modelled by drawing on multiple sources simultaneously. However, this approach generally restricts the model time horizon to that of an external RCT and cannot model the long-term falls risk progression without being supplemented by longer-term observational data.

#### Recurrent falls

Table 7 lists the models by model type category and their features relevant to characterising recurrent falls. The first feature is the transition entity, which is either the fall event or individual. The individual-transitioning models, particularly those with cycle length of one year or longer, should ensure that recurrent falls could occur to individuals during each cycle. A qualifying factor is the type of main fall-related event: if the event is less likely to recur within a year (e.g., hip fracture), then the need to characterise recurrent falls is reduced.

**Table 7:** Modelling methods for characterising recurrent falls

Study label (***n*** = 46)^**a**^	Transition entity^b^	Cycle length	Main fall-related event	Possible to model recurrent falls
**Binary decision model**				
Beard (2006) [53]	Fall event	N/A^c^	MA fall	Yes
Carande-Kulis (2015) [55]	Individual	N/A	MA fall	No
Comans (2009) [59]	Individual	N/A	Any fall	Yes
Frick (2010) [66]	Individual	N/A	Hip fracture	No
Hektoen (2009) [67]	Fall event	N/A	Any fall	Yes
Howland (2015) [71]	Individual	N/A	MA fall	Yes: targeted recurrent fall
Ippoliti (2018) [72]	Fall event	N/A	Hip fracture	Yes
Ling (2008) [75]	Individual	N/A	Any fall	No
Miller (2011) [77]	Individual	N/A	MA fall	No
Poole (2014) [83]	Fall event	N/A	Hip fracture	Yes
Sach (2007); (2010) [86, 87]	Fall event	N/A	Any fall	Yes
Velde (2008) [91]	Fall event	N/A	Any fall	Yes
Wu (2010) [93]	Individual	N/A	Any fall	Yes: targeted recurrent fall
**Static model**^d^				
Agartioglu (2020) [50]	Individual	N/A	Any fall	No
Albert (2016) [51]	Individual	N/A	Any fall	Yes
CSP (2016) [56]	Individual	N/A	MA fall	Yes
Day (2009); (2010) [60, 61]	Fall event	N/A	Any fall	Yes
Hirst (2016) [69]	Individual	N/A	Fractures	No
McLean (2015) [76]	Individual	N/A	Any fall	Yes: Adjusted risk
PHE (2018) [85]	Fall event	N/A	Any fall	Yes
Smith et al. (2016) [88]	Individual	N/A	MA fall	No
**Cohort-level Markov model**				
Alhambra-Borras (2019) [52]	Individual	1 year	Composite^e^	Yes: Composite^e^
BODE3 models	Individual	1 year	MA fall	No
Church (2011); (2012) [57, 58]	Individual	1 year	Any fall	No
Eldridge (2005) [63]	Individual	1 year	Any fall	No
Farag (2015) [64]	Individual	1 year	Any fall	No
Franklin (2019) [65]	Individual	1 year	Any fall	Yes
Honkanen (2006) [70]	Individual	1 year	Hip fracture	No
Johansson (2008) [73]	Individual	1 year	Hip fracture	No
Lee (2013) [74]	Individual	1 month	Any fall	Yes
Moriarty (2019) [79]	Individual	1 year	MA fall/Hip fracture	No
OMAS (2008) [81]	Individual	1 year	MA fall	No
Poole (2015) [84]	Individual	1 year	MA fall	No
RCN (2005) [34]	Individual	1 year	MA fall	No
Tannenbaum (2015) [89]	Individual	6 months	Any fall	Yes
Turner (2020) [90]	Individual	1 month	MA fall/Hip fracture	Yes
**Individual-level Markov model (microsimulation)**				
Hiligsmann (2014) [68]	Individual	6 months	Fractures	Yes
Mori (2017) [78]	Individual	1 year	Fractures	No
Nshimyumukiza (2013) [80]	Individual	1 year	Fractures	No
Zarca (2014) [94]	Individual	3 months	Hip fracture	Yes

*Abbreviations*: *BODE3* Burden of Disease Epidemiology, Equity and Cost-Effectiveness Programme studies, including Boyd (2020) [54], Deverall (2018) [62], Pega (2016) [82] and Wilson (2017) [92], *CSP* Chartered Society of Physiotherapy, *Int.* Intervention, *MA fall* Fall requiring medical attention, *N/A* Not applicable, *OMAS* Ontario Medical Advisory Secretariat, *PHE* Public Health England, *RCN* Royal College of Nursing

^a^See Table 2 for study references; parenthesised number refers to the number of models included in the table

^b^All Markov models conceive individuals (or proportion of individuals within cohort) transitioning between model states. Some binary decision and static models have fall events transitioning through health and economic sequelae

^c^Cycle length was not relevant or applicable to non-cycle-based models such as the decision tree

^d^All studies under this category, except Smith (2016) [88], used a decision tree model

^e^This model used a composite measure of health consequences including recurrent falls, fear of falling and mobility and balance problems. Hence, recurrent falls were captured within the composite measure

There were 23 models incapable of characterising recurrent falls. Seven of the 23 had fracture as the main event which are less likely to recur within a year [66, 69, 70, 73, 78–80]; whilst 16 models with falls as the main event were incapable of characterising recurrent MA or non-MA falls. Of 13 individual-transitioning models that *were* capable of characterising recurrent falls, three methods were mainly used: (1) modelling separate health states for recurrent fallers; (2) assigning average number of falls per faller; and (3) incorporating cycle lengths shorter than one year. Three models used (1) [51, 52, 56]: e.g., CSP [56] incorporated age- and gender-specific risks of experiencing recurrent falls conditional on having fallen. Three used (2) [56, 59, 65]: e.g., Franklin [65] assigned 2.8 falls as the average number of falls experienced per faller. Five used (3), incorporating the following cycle lengths: one month [74, 90]; three months [94]; and six months [68, 89]. Hiligsmann [68] and Zarca [94] had fractures as the main event yet incorporated short cycles. Tannenbaum [89] modelled higher falls risk in the second of the two six-month cycles for those who experienced a fall in the first. Other methods included: applying a negative binomial regression on individual-level data to adjust the falls risk for the number of falls per faller [76]; and targeting those who have experienced a fall immediately prior to the model baseline (‘targeted recurrent fall’ in Table 7) [71, 93]. No study employed model types incorporating time-to-event data (e.g., discrete event simulation) to overcome the limitation of set cycle lengths.

#### Falls risk factors

Table A4 in Supplementary Materials summarises the range of risk factors for falls and fall-related events incorporated by models that conducted primary analysis of individual-level data or used published epidemiological evidence (i.e., the first two approaches for characterising baseline risk in Table 6). For the eight models that conducted primary analysis, the individual-level granulation offered greater scope for incorporating a wide range of risk factors. For example, the four BODE3 models incorporated age, sex, ethnicity, and MA falls history as risk factors for MA fall, hospitalised fall and fatal fall. Smith [88] constructed a *de novo* MA falls risk prediction tool using diverse variables observed in the primary and secondary care routine data including history of fall/fracture; chronic disease diagnoses and history of all-cause secondary care utilisation.

Twenty-five models that used published evidence were more restricted in their incorporation of risk factors. Ten incorporated a single baseline risk or included age and/or sex as the only non-exogenous (i.e., not given at model baseline) risk factors [34, 50, 55, 66, 68, 71, 77, 83, 84, 93]. Only four incorporated non-injurious or non-MA falls as a risk factor for further falls within model simulation [57, 58, 64, 75]. No model incorporated fear of falling as a risk factor. Only three incorporated chronic diseases: osteoporosis [78, 80]; and depression and cognitive impairment [75]. Physical impairments as risk factors included: vitamin D deficiency [74, 94]; low bone mass density [80]; impaired gait or balance, leg weakness and functional impairment [75]; and functional dependency [70].

Models using internal intervention study evidence or external RCT data to characterise the baseline falls risk/rate (i.e., the last two approaches in Table 6) took the risk factors as given from the internal or external studies. For example, Day [60] used the inclusion criteria of external RCTs to define the risk profiles of six model subgroups receiving different interventions. A representative population survey was then used to estimate the subgroup sizes.

#### Falls health consequences

Table 8 summarises the health consequences of falls explicitly incorporated by models: i.e., studies included separate model states and probabilities for the consequence.

**Table 8:** Summary of health consequences of falls included in decision models^a^

Study label (***n*** = 46)^**b**^	Non-MA or non-injurious fall	MA or injurious fall	Fracture	Fatal fall	Fear of falling	Fall-induced LTC admission	Excess mortality
Agartioglu (2020) [50]	˟	Injury	Mix^c^				
Albert (2016) [51]	˟	MA					
Alhambra-Borras (2019)^d^ [52]	Com	Com			Com		
Beard (2006) [53]		MA					
BODE3 models		MA		˟			
Carande-Kulis (2015) [55]		MA		˟			
CSP (2016) [56]	˟	MA				˟	
Church (2011); (2012) [57, 58]	˟	MA	Mix	˟	˟	˟	
Comans (2009) [59]	˟	MA					
Day (2009); (2010) [60, 61]	˟	MA					
Eldridge (2005) [63]	˟	MA	Hip	˟	˟	˟	˟
Farag (2015) [64]	˟	MA		˟		˟	
Franklin (2019) [65]	˟	MA		˟		˟	˟
Frick (2010) [66]			Hip	˟			˟
Hektoen (2009) [67]	˟	Injury	Mix				
Hiligsmann (2014) [68]			Mix				˟
Hirst (2016) [69]			Mix			˟	
Honkanen (2006) [70]			Hip			˟	˟
Howland (2015) [71]		MA					
Ippoliti (2018) [72]			Hip				
Johansson (2008) [73]			Hip				˟
Lee (2013) [74]	˟	MA			˟		
Ling (2008) [75]	˟	MA				˟	
McLean (2015) [76]	˟	Injury	Mix				
Miller (2011) [77]	˟	MA					
Mori (2017) [78]			Mix			˟	˟
Moriarty (2019) [79]		MA	Hip			˟	˟
Nshimyumukiza (2013) [80]			Mix			˟	˟
OMAS (2008) [81]		MA	Mix	˟		˟	
Poole (2014) [83]			Hip				˟
Poole (2015) [84]		MA		˟		˟	
PHE (2018) [85]	˟	MA	Mix	˟	˟	˟	
RCN (2005) [34]		MA	Hip				
Sach (2007); (2010) [86, 87]	˟	MA					
Smith (2016) [88]		MA	Mix				
Tannenbaum (2015) [89]	˟	MA	Mix	˟	˟		˟
Turner (2020) [90]		MA	Mix	˟			
Velde (2008) [91]	˟	MA					
Wu (2010) [93]	˟	MA					
Zarca (2014) [94]			Hip				˟

*Abbreviations*: *BODE3* Burden of Disease Epidemiology, Equity and Cost-Effectiveness Programme studies, including Boyd (2020) [54], Deverall (2018) [62], Pega (2016) [82] and Wilson (2017) [92], *Com* Composite, *LTC* Long-term care, *MA fall* Fall requiring medical attention

^a^Only the health consequences that are explicitly incorporated by models are catalogued: i.e., studies included separate model states and probabilities for each consequence

^b^See Table 2 for study references; parenthesised number refers to the number of models included in the table

^c^The model incorporated multiple specified fracture types (e.g., hip, vertebral, wrist) or a general category of fracture without specifying the component fracture types

^d^This model used a composite measure of health consequences including recurrent falls, fear of falling and mobility and balance problems. Thus, the fall types and fear of falling are marked as ‘Composite’ (Com). The model also included a multivariate frailty index capturing physical, psychological and social aspects of vulnerability

There was noticeable between-study variation in the range of health consequences: 21 (45.7%) models included non-injurious or non-MA falls; 10 (21.7%) considered only fractures, of which six considered only hip fracture; 16 (34.8%) included fatal falls; and six (13.0%) fear of falling. In Church [57, 58], and Tannenbaum [89], fear of falling was associated with non-MA and MA fall incidence; in Lee [74] and PHE [85] only with MA fall; in Eldridge [63] fear could occur independently of falls. Fifteen (32.6%) incorporated fall-induced long-term care (LTC) admission; 12 (26.1%) incorporated excess mortality associated with major injuries.

Since a narrower range of health consequences would underestimate the cost-effectiveness of falls prevention, several models highlighted the exclusion of specific health consequences as a limitation: fear of falling [62, 82, 92]; fatal falls [76]; and non-fracture injuries [73, 78, 94]. Yet others advocated a narrower range to focus on falls with discernible health consequences [88] and generate conservative results [56]. Regardless, the between-study variation impairs outcome comparisons.

#### Health utilities

Table A5 in Supplementary Materials summarises the health utilities data used for CUA, the health states to which they are applied, and their sources. Twenty-nine models incorporated health utilities; 25 sourced them from external literature. EQ-5D was the most widely used instrument by 17 models; other instruments included HUI2, HUI3, and SF-6D. Four models concurrently used multiple instruments [70, 79, 89, 90]; two used values directly elicited from TTO exercises [63, 70].

The effect of an adverse event on health utility was depicted in three main approaches: (i) assigning an absolute decrement/loss to pre-event utility level; (ii) assigning proportional (i.e., multiplier) decrement to pre-event level; and (iii) assigning a specific health utility level to post-event state. An example of each are: EQ-5D loss of 0.200 for hip fracture in the 1st year, followed by loss of 0.060 for subsequent years [66]; multiplier 0.79 for hip fracture to pre-fracture level for 1st year, followed by multiplier 0.90 for subsequent years [94]; utility level of 0.050 for bad hip fracture requiring LTC admission [63]. These illustrate the significant between-model variation in the applied utility data reducing the comparability of CUA results.

#### Fall-related economic consequences

Table A6 summarises the economic consequences of falls from the health and social care perspective. The economic consequences were marked even if only their costs were considered without separate model states (unlike health consequences in Table 8). Care consequences directly attributed to falls are divided into six categories: (i) ambulatory care excluding emergency department (ED), e.g., GP visit and ambulance call-out; (ii) ED visit/admission; (iii) hospitalisation; (iv) rehabilitation, e.g., outpatient; (v) short-term social care, e.g., meal-on-wheels; and (vi) LTC. The cost of LTC admission was incorporated by 26 (56.5%) models. Studies noted the technical difficulty in costing LTC admission, particularly in identifying admissions directly attributable to falls and in stratifying costs by age and life expectancy at admission [56, 59, 62].

Four models incorporated all-cause (‘AC’), rather than fall-specific, care consequences using primary data from intervention studies [51, 52, 86, 87]. Six models incorporated comorbidity care costs [54, 62, 70, 73, 82, 92]. The four BODE3 models incorporated annual (all-cause) healthcare cost and cost of dying that varied by age and sex; falls prevention indirectly affected these costs by changing the life expectancy and age at death via fatal fall prevention. Johansson [73] incorporated age-stratified societal costs of added life-years measured in net consumption (production value minus consumption and care costs) but not cost of dying. In Honkanen [70], the annual healthcare cost and cost of dying were stratified by functional dependency and residence (community vs. nursing home); fracture prevention indirectly affected these by lowering the risks of functional dependency and nursing home admission. Comorbidity care costs are hence relevant to models that incorporate fatal falls, excess mortality and serious injuries that contribute to increased frailty and care dependency. Yet these costs were included in only six (listed above) of 24 models that incorporated fatal falls and/or excess mortality.

### Narrative synthesis: falls prevention intervention features

As detailed in Table 1, falls prevention intervention features are synthesised based on: (1) intervention access pathways; (2) falls risk identification methods; (3) intervention resource-use and cost; (4) intervention efficacy; and (5) wider health effects of interventions beyond falls prevention. Table A7 in Supplementary Materials provides additional detail on intervention components by study.

#### Intervention access pathway

Table 9 categorises all model-evaluated interventions by access pathway – reactive, proactive, self-referred or unclear – and intervention type. Of 101 interventions in total – counting multiple forms per study separately – nearly half (49) had unclear pathway descriptions. The most common pathway was proactive with 29 interventions, followed by self-referred (16) and reactive (7).

**Table 9:** Intervention access pathways by intervention type [number of intervention forms]^a^

Intervention type (total N)	Reactive	N	Proactive	N	Self-referred	N	Unclear	N
Exercise (33)		0	Alhambra-Borras (2019) [52]; CSP (2016) [56]; Day (2009) [60]; Eldridge (2005) [63]; Franklin (2019) [65] [3]; Nshimyumukiza (2013) [80]; RCN (2006)	9	Carande-Kulis (2015) [55] [2]; Day (2009) [60]; Day (2010) [61]; Deverall (2018) [62] [3]; Eldridge (2005) [63]; Hektoen (2009) [67]; McLean (2015) [76]	10	Church (2011) [57] [3]; Church (2012) [58] [4]; Frick (2010) [66] [2]; Mori (2017) [78]; OMAS (2008) [81]; PHE (2018) [85] [3]	14
HAM (11)	Day (2009) [60];^b^ PHE (2018) [85]	2	Franklin (2019) [65]; Wilson (2017)^c^ [92]	2	Wilson (2017) [92]	1	Agartioglu (2020) [50]; Church (2012) [58]; Ling (2008) [75]; OMAS (2008) [81]; Pega (2016) [82]; Frick (2010) [66]	6
Medication review and modification (10)		0	Day (2009) [60]; Tannenbaum (2015) [89]; Turner (2020) [90]; Velde (2008) [91]	4		0	Church (2011) [57]; Church (2012) [58]; Frick (2010) [66]; Hirst (2016) [69]; Moriarty (2019) [79]; OMAS (2008) [81]	6
Cataract surgery (5)		0	Sach (2007) [86]; Sach (2010) [87]	2		0	Boyd (2020) [54]; Church (2011) [57]; Church (2012) [58]	3
Vitamin D supplement (11)	Nshimyumukiza (2013) [80]	1	Hiligsmann (2014) [68]; Lee (2013) [74] [2]; Nshimyumukiza (2013) [80]; Zarca (2014) [94] [3]	7		0	Poole (2014) [83]; Poole (2015) [84]; Frick (2010) [66]; OMAS (2008) [81]	4
Other single-component (6)	Day (2009) [60] – cardiac pacing	1		0	Farag (2015) [64] – non-specific intervention	1	Church (2011) [57] – cardiac pacing; Church (2012) [58] – cardiac pacing; Honkanen (2006) [70] – hip protector; OMAS (2008) [81] – gait stabiliser	4
MF int. and MRA (17)	Day (2009) [60]; Eldridge (2005) [63]	2	Eldridge (2005) [63]; Ippoliti (2018) [72]; RCN (2005) [34]; Smith (2016) [88]; Wu (2010) [93]	5	Albert (2016) [51]	1	Church (2011) [57] [2]; Church (2012) [58] [3]; Comans (2009) [59] [2]; Frick (2010) [66] [2]	9
MC int. (7)	Howland (2015) [71]	1		0	Beard (2006) [53]; Carande-Kulis (2015) [55]; Johansson (2008) [73]	3	Church (2011) [57]; Church (2012) [58]; Miller (2011) [77]	3
**Total (101)**		7		29		16		49

*Abbreviations*: *CSP* Chartered Society of Physiotherapy, *HAM* Home assessment and modification, *MC int.* Multiple-component intervention, *MF int.* Multifactorial intervention, *MRA* Multifactorial risk assessment only, *OMAS* Ontario Medical Advisory Secretariat, *PHE* Public Health England, *RCN* Royal College of Nursing

^a^See Table 2 for study references; all 46 models are included. Number of intervention form is one unless specified

^b^For all-cause, not fall-related, hospital inpatients

^c^In alternative intervention scenario

Models with unclear access pathways frequently failed to mention how specific groups eligible for intervention were identified and recruited. For example, Church [58] evaluated group exercise, HAM, and multifactorial intervention given to the high falls risk subgroup within the target population but didn’t mention how this subgroup would be identified; it similarly failed to mention how specific patient groups for cataract surgery, psychotropic medication withdrawal, and cardiac pacing would be identified.

Three models considered multiple pathways for the same intervention. Eldridge [63] evaluated a falls risk screening and referral programme that encompassed all three pathways operating in tandem: falls patients at A&E and hospital would be screened by the falls risk assessment tool (FRAT) and referred to a multidisciplinary falls clinic (reactive pathway); primary care professionals would screen and refer high-risk individuals to the falls clinic or bi-disciplinary treatment (proactive); the low-risk individuals not referred could still self-refer to the bi-disciplinary treatment (self-referred). In Nshimyumukiza [80], vitamin D and calcium supplementation could be initiated proactively after fracture risk screening or reactively after fracture incidence. Wilson [92] evaluated a self-referred HAM in the base case and a proactive HAM (targeted at those with MA falls history) as an alternative scenario.

#### Falls risk screening

Falls risk screening is required to identify subgroups within target population eligible for intervention or specific risk/patient groups serving as the target population itself. Four methods were used to model the screening process: (i) using primary data to assign individual-level distribution of falls risk factors; (ii) using external data to assign cohort-level distribution of falls risk factors; (iii) using external data on screening efficacy (i.e., sensitivity and specificity) without assigning distributions; and (iv) incorporating screening cost only. Two models used (i): Eldridge [63] used primary survey data to estimate falls risk according to FRAT; Smith [88] used routine data to predict falls risk. Three used (ii): Lee [74] assigned age- and sex-stratified prevalence of vitamin D insufficiency; Zarca [94] a lognormal distribution of vitamin D level; and Nshimyumukiza [80] a distribution of BMD level. Screening detected (with perfect precision) vitamin D or BMD insufficiency for intervention referrals.

Two used (iii): CSP [56] assumed that the sensitivity and specificity of timed-up-and-go (TUG) test were both 87% regardless of the underlying distribution of gait/balance impairment; following screening, the 11% highest risk individuals from each five-year age group were referred to physiotherapy. The latter assumption is problematic given that older age groups likely have higher proportions of high-risk individuals (unless the test cut-off levels varied across age groups). Franklin [65] similarly incorporated fixed efficacies for TUG and quantitative TUG (QTUG) without modelling the underlying gait/balance distribution. A disadvantage of this approach is that subgroup variation in the joint distributions of diverse falls risk factors would introduce subgroup differences in the screening efficacy not explored by Franklin [65]. Seven used (iv) [34, 60, 68, 86, 87, 89, 90]: e.g., RCN [34] included the cost of identifying eligible high-risk individuals.

#### Intervention resource-use and cost

Table A8 in Supplementary Materials summarises the intervention resource-use and cost from the public sector perspective (the societal intervention costs will be presented in a future publication). The resources are divided into auxiliary resources facilitating implementation (access, compliance and long-term sustainability) and resources generating therapeutic effects. Exercise and multiple-component interventions were most likely to incorporate these auxiliary resources: e.g., marketing to assist exercise uptake [55]. Falls risk screening resources were likewise auxiliary. Two models failed to cost their screening tools [56, 88]. Three models included set-up costs [63, 65, 77]. There were noticeable between-study variations in resource incorporation for each intervention type.

Therapeutic resources included labour, training, transport, venue and overheads, and health technology and equipment. Labour was the most widely costed resource, including labour performed by nonprofessional volunteers and reimbursed by the public sector [51, 62, 71, 77]. Models evaluating technology-based interventions such as hip protector and gait stabiliser tended to neglect the cost of contributory labour [69, 70, 74, 80, 81, 83, 84, 89]. Training costs were concentrated in exercise interventions; only three non-exercise evaluations incorporated them [51, 55, 77]. Staff transport costs were concentrated in models evaluating exercise, HAM, and multifactorial intervention. Venue costs and overheads were generally included as simple supplements to per-participant labour cost: e.g., Frick [66] increased the labour cost by 50% to account for overheads; Velde [91] by 72%. All intervention types required some technology and equipment; yet not all models detailed or costed them. For example, Frick [66] costed the labour but not the equipment for HAM.

In costing the interventions, preserving the distinction between fixed and variable (i.e., per-participant) costs had a significant impact on results. For example, Eldridge [63] incorporated the fixed cost in running the falls clinic which, under a low uptake rate (6.5% of eligible population), increased the per-participant cost and reduced the cost-effectiveness. Likewise, Comans [59] included annual fixed cost of multifactorial intervention, which determined the uptake rate required to break-even financially. Despite this, 36 (78.3%) models only incorporated per-participant costs, some deliberately translating fixed costs into per-participant rates [60, 61, 77, 92].

#### Intervention efficacy

Table 10 specifies the fall-related event used for the intervention efficacy and, in parenthesis, the main fall-related event used to characterise falls risk/rate. Twelve (26.1%) models did not incorporate matching events (highlighted in bold). Thirty-six (78.3%) sourced efficacy data from internal or external RCTs and meta-analyses, while three used observational studies [69, 80, 89]. On using external RCT data, several models questioned whether it can be generalised to routine practice [55, 60, 61, 71, 83, 85, 93]; Mori [78] down-adjusted the RCT-based efficacy by 40% for generalisation. The fifth column details the efficacy and, in parenthesis, incidence metrics. The metrics did not match in 12 (26.1%) models: e.g., Deverall [62] applied RaR on individual falls risk.

**Table 10:** Summary of intervention efficacy data used by decision models

Study label (***n*** = 46)^**a**^	Intervention type	Efficacy (main model) fall-related event	Data source type	Efficacy (incidence) metric	Effectiveness period^b^ (model time horizon)
Agartioglu (2020) [50]	HAM	Any fall (any fall)	External meta-an. and internal RCT	RR (risk)	1 year (1 year)
Albert (2016) [51]	Multifactorial int.	Any fall (any fall)	Internal non-randomised	RR (risk)	1 year (1 year)
Alhambra-Borras (2019) [52]	Group exercise	Composite^c^ (composite)	Internal quasi-experiment	RR (risk)	1 year (lifetime)
Beard (2006) [53]	Multifactorial int.	Hospital fall (hospital fall)	Internal quasi-experiment	RaR (rate)	5-year sustainability (5 years)
Boyd (2020) [54]	Expedited cataract surgery	Any fall (**MA fall**)	External RCT	RR (risk)	1 year^d^ (lifetime)
Carande-Kulis (2015) [55]	Multiple types	Any fall (**MA fall**)	External RCTs	RR or RaR (**risk**)	1 year (1 year)
CSP (2016) [56]	Physiotherapy	Any fall (**MA fall**)	External meta-an.	RaR (**risk**)	1 year (1 year)
Church (2011) [57]	Multiple types	Any fall (any fall)	External meta-an.	RaR (**risk**)	Efficacy durability differ by int. type (10 years)
Church (2012) [58]	Multiple types	Any fall (any fall)	External meta-an.	RaR (**risk**)	Efficacy durability differ by int. type (lifetime)
Comans (2009) [59]	Multifactorial int.	Any fall (any fall)	External RCT	RaR (**risk & rate**)	1 year (1 year)
Day (2009) [60]	Multiple types	Any fall (any fall)	External RCTs	RaR (rate)	Efficacy durability same as model time (1, 2 or 5 years)
Day (2010) [61]	Tai Chi	Any fall (any fall)	External meta-an.	RaR (rate)	1 year (1 year)
Deverall (2018) [62]	Multiple exercise types	Any fall (**MA fall**)	External meta-an.	RaR (**risk**)	Varying persistence (25 years)
Eldridge (2005) [63]	FRAT; balance and gait int.	Any fall (any fall)	External meta-an.	RR (risk)	Not specified (lifetime)
Farag (2015) [64]	Unspecified	Any fall (any fall)	Assumption	RR (risk)	Not specified (lifetime)
Franklin (2019) [65]	Multiple types	Any fall (any fall)	External meta-an. and RCTs	RR and RaR (risk & rate)	1 year (2 years)
Frick (2010) [66]	Multiple types	Any fall (**hip fracture**)	External meta-an.	RR (risk)	1 year (1 year^e^)
Hektoen (2009) [67]	Home exercise	Any fall (any fall)	External RCT	RaR (rate)	1 year (1 year)
Hiligsmann (2014) [68]	Vit. D + calcium supplement	Mix fracture; (mix fracture)	External meta-an.	RR (risk)	6 years^f^ (lifetime)
Hirst (2016) [69]	Buprenorphine vs. Tramadol	Mix fracture (mix fracture)	External surveys	OR (risk)	1 year (1 year)
Honkanen (2006) [70]	Hip protector	Hip fracture (hip fracture)	External RCT	RR (risk)	Varying persistence (20 years)
Howland (2015) [71]	Matter of Balance lay-led	MA fall (MA fall)	External RCT	RR (risk)	1 year (1 year)
Ippoliti (2018) [72]	Multifactorial int.	Hip fracture (hip fracture)	Policy variable	RaR (rate)	3 years (3 years)
Johansson (2008) [73]	Multifactorial int.	Hip fracture (hip fracture)	Internal quasi-experiment	RaR (**risk**)	1 year (lifetime)
Lee (2013) [74]	Vit. D screening & supplement	Any fall (any fall)	External meta-an.	RR (risk)	2.5 years (3 years)
Ling (2008) [75]	HAM	Any fall (any fall)	External RCT	RR (risk)	1 year (1 year)
McLean (2015) [76]	Exercise	Any fall (any fall)	Internal RCT	RR (risk)	1.5 years (1.5 years)
Miller (2011) [77]	Matter of Balance lay-led	Any fall (any fall)	Policy variable	RR (risk)	2 years (2 years)
Mori (2017) [78]	Exercise & bisphosphonate	Mix fracture (mix fracture)	External meta-analyses	RR or RaR (**risk**)	1/2 year maintenance (lifetime)
Moriarty (2019) [79]	Withdrawal of PIP mediations	MA fall/Hip fracture (MA fall/hip fracture)	External RCTs	RR (risk)	Lifetime persistence (35 years)
Nshimyumukiza (2013) [80]	Exercise, Vit. D + calcium & osteoporosis int.	Mix fracture (mix fracture)	External meta-an. & surveys	RR (risk)	Lifetime sustainability (lifetime)
OMAS (2008) [81]	Multiple types	Any fall (**MA fall**)	Internal meta-an.	RR (risk)	Lifetime persistence for 1st year adherers (lifetime)
Pega (2016) [82]	HAM	Any fall (**MA fall**)	External meta-an.	RaR (**risk**)	Lifetime or 10-year efficacy (lifetime)
Poole (2014) [83]	Vit. D supplement	Hip fracture (hip fracture)	External meta-an.	HR (rate)	1 year (1 year)
Poole (2015) [84]	Vit. D supplement	Any fall (**MA fall**)	External meta-an.	RR (risk)	5 years maintenance (5 years)
PHE (2018) [85]	Multiple types	Any fall (any fall)	External meta-an. and RCTs	RaR (rate)	2 years (2 years)
RCN (2005) [34]	Multiple types	Any fall (**MA fall**)	External meta-an.	RR (risk)	Not specified (lifetime)
Sach (2007); (2010) [86, 87]	Expedited cataract surgery	Any fall (any fall)	Internal RCT	RaR (rate)	Lifetime efficacy durability (lifetime)
Smith (2016) [88]	Risk prediction; Multifactorial int.	Any fall (**MA fall**)	External meta-an.	RaR (**risk**)	1 year (1 year)
Tannenbaum (2015) [89]	Insomnia treatments	Any fall (any fall)	External surveys	OR (risk)	Not specified (1 or 5 years)
Turner (2020) [90]	Sedative withdrawal	Hip/non-hip fracture (**MA fall, hip/non-hip fracture**)	External RCT	RaR (**risk**)	1 year (1 year)
Velde (2008) [91]	FRID withdrawal	Any fall (any fall)	Internal non-randomised	RaR (rate)	1 year (1 year^e^)
Wilson (2017) [92]	HAM	Any fall (**MA fall**)	External meta-an.	RaR (**risk**)	Lifetime or 10-year efficacy (lifetime)
Wu (2010) [93]	Multifactorial int.	Any fall (any fall)	External meta-an.	RR (risk)	1 year (1 year)
Zarca (2014) [94]	Vit. D screening & supplement	Vit. D level (vit. D level)	External meta-an. and RCT	Other^g^	Varying persistence (lifetime)

*Abbreviations*: *CSP* Chartered Society of Physiotherapy, *FRID* Fall-risk-increasing drug, *HAM* Home assessment and modification, *MA fall* Fall requiring medical attention, *Met-An.* Meta-analysis, *OMAS* Ontario Medical Advisory Secretariat, *OR* Odds ratio, *PHE* Public Health England, *PIP* Potentially inappropriately prescribed, *RaR* Rate ratio, *RCN* Royal College of Nursing, *RCT* Randomised controlled trial, *RR* Relative risk, *Vit. D* Vitamin D

^a^See Table 2 for study references; parenthesised number refers to the number of models included in the table

^b^The effectiveness period is a function of efficacy durability and implementation sustainability. Key determinants of sustainability are demand-side *persistence* and supply-side *maintenance*; not all studies made this distinction

^c^This model used a composite outcome including fall-related consequences – recurrent falls, fear of falling and mobility and balance problems – and multivariate frailty index – physical, psychological and social aspects of vulnerability

^d^Also includes benefit of cataract surgery on vision: permanent increase of 0.0565 quality-adjusted life year per person

^e^The study contained a single one-year cycle but included lifetime healthcare costs and effects of hip fracture

^f^After three years of vitamin D and calcium supplementation, the efficacy would remain for further three years, though declining linearly over that period

^g^Supplementation increased the vitamin D level which in turn reduced hip fracture risk

Table 10 also compares the model horizon with the ‘effectiveness period’; i.e., a function of efficacy durability and implementation sustainability. Several studies contained significant disparities between the model horizon and the effectiveness period. For example, Johansson (2017) restricted the effectiveness period to one year within lifetime horizon to produce conservative outcomes. Several lifetime models incorporated long-term effectiveness for individuals who persisted in intervention uptake [62, 70, 79, 81, 94]. Models made diverse assumptions on post-implementation efficacy often without justification [57, 58, 68, 77, 85]. For example, Church [57, 58] incorporated lifetime efficacy for expedited cataract surgery and cardiac pacing but one-year efficacy for other interventions; unsurprisingly, the latter were significantly less cost-effective. Some deliberately curtailed the model horizon to reduce the discrepancy with the effectiveness period [60, 61, 84, 85].

#### Wider health effects of interventions

Few models incorporated wider health effects of interventions beyond falls prevention. Hiligsmann [68] evaluated a scenario where vitamin D and calcium supplementation reduced the background mortality risk. Alhambra-Borras [52] incorporated the effect of falls prevention exercise on frailty reduction. Boyd [54] allowed cataract surgery to generate QALY gain through vision improvement. Models that incorporated all-cause care costs captured wider health effects without specifying the mechanism [51, 86, 87]. Other models mentioned their non-incorporation as a limitation [55, 56, 60–62, 71, 73, 77, 78, 80, 94]. Deverall [62], for example, stated that the non-incorporation of exercise benefit on cardiovascular disease (CVD) risk reduction potentially biased the evaluation against the ethnic Maori subgroup who have greater CVD risk.

Two models incorporated *adverse* health effects and process costs of interventions. Hirst [69] considered the side-effect of transdermal buprenorphine as a replacement for (more fall-risk-inducing) tramadol in chronic pain management. Honkanen [70] expressed the process cost of hip protector use through a health utility decrement of 0.010 for each year of use. Due to the decrement, younger groups aged 65 and 70 experienced overall QALY *loss* from hip protector use despite fractures being prevented.

### Narrative synthesis: evaluation methods

As detailed in Table 1, evaluation methods are synthesised based on three specific aspects: (i) model validation methods and results; (ii) methods for assessing parameter uncertainty; and (iii) alternative scenarios evaluated. Additionally, we focus on how different evaluation methods could lead to alternative commissioning recommendations.

#### Model validity

Four validity types influence the credibility of model results: structural/face; internal; external; and cross [44]. Seven models involved experts and stakeholders in model development to achieve structural validity prospectively [60, 71, 79, 80, 85, 90, 94]. For example, PHE [85] engaged two groups of stakeholders: a Steering Group of national falls prevention experts informing the model structure, and a User Group of local commissioners advising on model usability. Hirst [69] explicitly stated the purpose of alternative scenario analyses as retrospectively validating the model structure.

Six studies assessed the external model validity [68, 73, 78, 80, 83, 94]. For example, Nshimyumukiza [80] compared the predicted fracture incidence and age-specific mortality rates to those reported in published literature and found less than 5% divergence. Only four studies reported conducting verification steps or sensitivity analyses to ensure internal validity [68, 73, 79, 94]. Cross validity assessment by comparing the model results with those of previous models was the most common form of validation; yet 13 (28.3%) did not report having conducted it [55–57, 60, 61, 63, 65, 69, 72, 77, 81, 83, 85]. Only Zarca [94] conducted all four validations; four conducted three [68, 73, 79, 80]. Overall, model validation is not yet a common methodological and reporting practice in this field.

#### Assessing parameter uncertainty

Table A9 in Supplementary Materials summarises the parameters unilaterally varied in deterministic sensitivity analysis (DSA) to assess their impact on outcomes. It also summarises the methods used to conduct probabilistic sensitivity analysis (PSA) assessing the impact of joint parameter uncertainty. The DSA parameters are divided into falls epidemiology and falls prevention intervention parameters. A distinction was made between parameter variations to assess parameter uncertainty and those depicting alternative scenarios based on studies’ descriptions of the purpose of the variations.

Twelve (26.1%) models conducted no assessment of parameter uncertainty. Of 21 models that conducted DSA, there was a wide between-study variation in the number of parameters assessed, ranging from two to 12. Twenty-eight (60.9%) conducted PSA. The cost-effectiveness acceptability curve (CEAC) which plots the probability of each intervention being the most cost-effective option at each cost-effectiveness threshold was the most frequently used presentation method (*n* = 18). Only Agartioglu [50] plotted the cost-effectiveness acceptability frontier (CEAF) which marks the threshold at which an intervention produces the highest expected value relative to alternatives across simulated runs. Only Albert [51] conducted value of information analysis, estimating that the cost-effectiveness of multifactorial intervention would improve under simulation runs that excluded uncertainty over health utility decrement parameters.

#### Scenario analyses

Table A10 in Supplementary Materials summarises the scenarios that were evaluated by the studies, categorised into areas of falls epidemiology, falls prevention intervention and evaluation framework. Most (*n* = 38; 82.6%) models evaluated at least one alternative scenario. Of these, there was a wide variation in the number of scenarios, ranging from one to 10. With some exceptions [73, 80, 93], there was a lack of clarity on how the scenarios were chosen among the range of possible options.

### Narrative synthesis: evaluation outcomes

Table 11 summarises the evaluation outcomes of 12 general population, lifetime models. See Table A11 in Supplementary Materials for outcome summaries of non-general population and/or non-lifetime models. All models except OMAS [81] conducted CUA; hence their ICERs (converted to 2021 US$) are compared to the NICE cost-effectiveness threshold of US$41900 (£30,000) per QALY. All models targeted those aged 65+ except RCN [34] which targeted 60+. Models incorporated diverse fall-related health and economic consequences (see Tables 8 and A6) which hamper between-study comparison. All models except Nshimyumukiza [80] and Zarca [94] were Markov cohort models but mentioned no tunnel states for age-related progression in falls risk. This likely disadvantages the outcomes for younger subgroups at baseline whose stymied age-related risk progression means smaller lifetime intervention benefit derived in terms of proportional reduction in falls risk. Only three models were validated (other than cross-validation): Johansson [73] internally and externally; Nshimyumukiza [80] structurally and externally; and Zarca [94] structurally, externally and internally. The last column of Table 11 notes the main methodological caveats for each model that are relevant for commissioning. The methodological quality checklist scores in Tables 3, 4 and 5 should also be noted.

**Table 11:** Features and evaluation outcomes of general population, lifetime horizon decision models

Study label (***n*** = 12)^**a**^	Target population; Analysis; Perspective	Intervention [Comparator]	Evaluation outcomes^**b**^	Methodological caveats
Church (2012) [58]	CD adults aged 65+; CUA/CEA; Public sector	(a) General population – Group exercise; Home exercise; Tai Chi; Multi-component int.; Multifactorial int.; Multifactorial risk assessment; (b) High-risk population – Group exercise; HAM; Multifactorial int. [NR; Cross comparisons]	***Ratio*:** (a) General – Tai Chi ICER US$38,735 per QALY vs. NR; other interventions dominated; (b) High-risk – Group exercise ICER US$44,633 per QALY vs. NR; HAM ICER US$50,696 per QALY vs. NR; Multifactorial int. dominated. ***Aggregate*:** Reports incremental cost, no. of falls avoided and QALY gain per intervention, but all interventions have same reach^c^ (including those targeting high-risk and specific subgroups), and hence cannot compare aggregate impacts. ***Parameter uncertainty*:** DSA – int. cost and efficacy had largest impact on group exercise ICER. PSA – CEAC. ***Scenarios*:** No fear of falling had the largest impact on group exercise ICER among parameter changes.	Recurrent falls not characterised; Unclear falls risk progression;^d^ Unclear intervention reach;^e^ Unclear how high-risk subgroup identified; Mismatch between falls incidence and efficacy metrics
Deverall (2018) [62]	CD adults aged 65+; CUA; Public sector, Societal	Exercise – (i) Peer-led group exercise; (ii) Home exercise; (iii) Commercial exercise [NR]	***Ratio*:** (i) Peer-led group exercise ICER US$6323 per QALY vs. NR; (ii) Home exercise ICER US$5486 per QALY vs. NR; (iii) Commercial exercise ICER US$46,733 per QALY vs. NR. ***Aggregate*:** For base case, home exercise generated 47,100 additional QALYs at incremental cost of US$225 m relative to NR; this compares to 42,000 and 42,300 QALYs for group exercise and commercial exercise at US$426 m and US$1550 m incremental cost relative to NR, respectively. Hence, home exercise dominated group and commercial exercises. ***Parameter uncertainty***: DSA – efficacy and utility decrement had largest impact on ICER. PSA – 95% UI; CEAC. ***Scenarios***: Subgroup analyses showed higher ICERs for Maori and men; equity analyses showed higher ICERs can be mainly attributed to their shorter life expectancies.^e^	Routine data lacks individual identifiers;^f^ Recurrent falls not characterised; Unclear falls risk progression;^d^ No background transition in health utilities;^g^ Includes comorbidity care costs; Mismatch between falls incidence and efficacy metrics; No tiered threshold for evaluating societal outcomes;^h^ No scenario estimating equity-efficiency trade-off.^e^
Eldridge (2005) [63]	Adults aged 65+ in community or nursing home; CUA; Public sector	Falls risk screening + multifactorial int. or exercise [UC]	***Ratio***: Not reported. ***Aggregate***: Intervention reduced the number of fallers by 2.8% over one year under base case (6.5% uptake of screening). ***Parameter uncertainty***: PSA – 40% probability intervention is cost-effective at US$41,900 (£30,000) per QALY threshold. ***Scenarios***: 100% screening uptake would reduce number of fallers by 11.3% over one year; 100% screening uptake and 100% self-referred exercise uptake would reduce number of fallers by 15.0%; impact of uptake increase on ICER not reported.	Recurrent falls not characterised; Unclear falls risk progression;^d^ No background transition in health utilities;^g^ Incorporated fixed intervention costs
Farag (2015) [64]	CD adults aged 65+ without prior fall; CUA; Public sector	Non-specific falls prevention int. with relative risk of 0.75 and per-participant cost of US$587 [NR]	***Ratio*:** ICER of US$24,190 per QALY vs. NR ***Aggregate*:** Incremental cost and QALY gain outcomes per person can be scaled up but unclear to what extent. ***Parameter uncertainty*:** DSA – falls risk and LTC cost had largest impact on ICER. PSA – CEAC; 57% probability of being cost-effective at AUS$50,000 (US$41,809) threshold. ***Scenarios*:** e.g., variation in uptake rate had little impact on ICER	Recurrent falls not characterised; Unclear falls risk progression;^d^ No discounting
Honkanen (2006) [70]	Adults aged 65+ living in community at baseline; CUA/ROI; Public sector	Hip protector [NR]	***Ratio***: Women, baseline age 65 – intervention dominated by NR; Women, age 70 – intervention dominated by NR; Women, age 75 – ICER of US$27,006 per QALY; Women, age 80 – intervention dominates NR; Women, age 85 – intervention dominates NR; Men, age 65 – intervention dominated by NR; Men, age 70 – intervention dominated by NR; Men, age 75 – intervention dominated by NR; Men, age 80 – ICER of US$184,609 per QALY; Men, age 85 – intervention dominates NR. ***Aggregate***: Prevented fractures, incremental cost and QALY gain outcomes per person can be scaled up but unclear to what extent. ***Parameter uncertainty***: DSA – base case results robust. PSA – 68% probability of being cost-effective at US$50,000 (US$710042021 price) threshold for women age 75; 61% for men age 85. ***Scenarios***: Intervention is less cost-effective for functionally dependent subgroup – e.g., intervention no longer dominant for women age 80 and 85, though still cost-effective at US$71,004 threshold (point estimates not reported)	Unclear falls risk progression;^d^ Includes comorbidity care costs.
Johansson (2008) [73]	CD adults aged 65+ (*n* = 5500); CUA; Societal	Multifactorial and environmental int.^i^ [UC]	***Ratio*:** Intervention dominates comparator ***Aggregate*:** Total int. cost of US$895,137; total costs savings of US$904,986; total QALY gain of 35.16 ***Parameter uncertainty*:** No DSA. PSA – scatter plot ***Scenarios*:** Scenarios that made intervention no longer dominant – doubled fracture risk; lower fracture cost; inclusion of net consumption care cost;^j^ higher discount rate; no health/cost consequences of fracture beyond 1st year; 25% rise in int. cost	Unclear falls risk progression;^d^ Includes comorbidity care costs (net consumption); Quasi-experimental study for effectiveness evidence; No tiered threshold for evaluating societal outcomes;^h^ Internal and external validities assessed
Nshimyu-mukiza (2013) [80]	Women aged 65+ (subgroup within women aged 40+); CUA/CEA; Public sector	Fracture risk screening + Physical activity (PA), Vitamin D & calcium and/or Osteoporosis screening & treatment [NR; Cross comparisons]	***Ratio***: No screening + PA dominates NR; BMD/CAROC screening + PA + Vit D & calcium produces ICER of US$57,279 relative to No screening + PA and dominates all other strategies. ***Aggregate***: Incremental cost and QALY gain per person can be scaled up (total population reported). ***Parameter uncertainty***: No DSA. PSA – CEAC; 75% probability that BMD/CAROC +PA + Vit D & calcium is cost-effective to No screening + PA under threshold of CAD$50,000 (US$517892021 price). ***Scenarios***: Rankings of strategies under CUA and CEA robust under variations in single or multiple parameters.	Incorporates incoming cohorts; No background transition in health utilities;^g^ Structural and external validities assessed
OMAS (2008) [81]	CD adults aged 65+; CEA/ROI; Public sector	(i) Exercise; (ii) HAM; (iii) Vit D & calcium; (iv) Gait stabiliser; (v) Psychotropics withdrawal.^k^ [NR]	***Ratio*:** All interventions dominate NR for men and women ***Aggregate*:** Reports net cost saving per person which can be scaled up to total for each intervention subgroup at regional level ***Parameter uncertainty*:** No analysis ***Scenarios*:** No analysis	Recurrent falls not characterised; Unclear falls risk progression;^d^ Mismatch between intervention need and falls risk;^k^ Parameter uncertainty not assessed
Pega (2016) [82]	CD adults aged 65+; CUA; Public sector	HAM [NR]	***Ratio*:** HAM produces ICER of US$7155 per QALY vs. NR. ***Aggregate*:** For base case, total int. cost was US$115.2 m, total net cost vs. NR US$87.4 m and total QALY gain 34,000. ***Parameter uncertainty*:** DSA – impact on ICER not assessed, fatal falls risk and falls risk most impactful for incremental cost and QALY, respectively. PSA – 95% UI for base case ICER between below zero to US$15,901 per QALY. ***Scenarios*:** For secondary prevention scenario,^l^ ICER was US$1591 per QALY, total int. cost US$14.2 m, total net cost vs. NR, US$4.9 m, and total QALY gain 20,100. Subgroup analyses showed higher ICERs for Maori and men; equity analyses showed higher ICERs can be mainly attributed to their shorter life expectancies.^e^	Routine data lacks individual identifier;^f^ Recurrent falls not characterised; Unclear falls risk progression;^d^ No background transition in health utilities;^g^ Includes comorbidity care costs; Mismatch between falls incidence and efficacy metrics; Unrealistic efficacy duration; Joint parameter uncertainty not assessed; No scenario estimating equity-efficiency trade-off.^e^
RCN (2005) [34]	CD adults aged 60+; CUA; Public sector	Exercise; Multifactorial intervention [NR]	***Ratio***: Multifactorial intervention for high-risk group dominates NR; Exercise for high-risk group produces ICER of US$18,425 per QALY relative to NR. ***Aggregate***: Not reported. ***Parameter uncertainty***: No DSA. PSA – scatter plot ***Scenarios***: No analysis	Recurrent falls not characterised; Unclear falls risk progression;^d^ Unclear intervention reach.^c^
Wilson [92]	CD adults aged 65+; CUA; Public sector	HAM [NR]	***Ratio***: HAM produces ICER of US$4358 per QALY vs. NR. ***Aggregate***: For base case, total int. cost was US$7.7 m, total net cost vs. NR US$6.7 m and total QALY gain 2800. ***Parameter uncertainty***: DSA – efficacy had largest impact on base case ICER. PSA – 95% UI for base case ICER between below zero to US$12,165 per QALY. ***Scenarios***: For secondary prevention scenario,^l^ ICER was US$557 per QALY, total int. cost US$687,151, total net cost vs. NR, US$72,626, and total QALY gain 1420. For primary prevention scenario,^m^ ICER was US$7633 per QALY, total int. cost US$7.0 m, total net cost US$6.6 m and total QALY gain 1520. Subgroup analyses showed higher ICER for Maori; equity analyses showed higher ICER can be mainly attributed to Maori’s shorter life expectancy.^e^	Routine data lacks individual identifiers;^f^ Recurrent falls not characterised; Unclear falls risk progression;^d^ No background transition in health utilities;^g^ Includes comorbidity care costs; Unclear intervention reach;^c^ Mismatch between falls incidence and efficacy metrics; Unrealistic efficacy duration; Joint parameter uncertainty not assessed; No scenario estimating equity-efficiency trade-off.^e^
Zarca (2014) [94]	Adults aged 65+ without previous hip fracture; CUA/CEA; Public sector	Vitamin D – (i) Universal supplementation; (ii) Supplement then screen for calibration; (iii) Screen then supplement [NR; Cross comparisons]	***Ratio***: Universal supplementation was dominated by other strategies; Supplement then screen strategy produces ICER of US$7758 per QALY vs. NR; Screen then supplement strategy produces ICER of US$7307 per QALY vs. Supplement then screen and US$7605 per QALY vs. NR. ***Aggregate***: Difficult to compare strategies without data on intervention reach.^c^ Possible that Screen then supplement strategy has smallest reach. Estimating total cost of Screen then supplement to be US$111.7 m for 800,000 persons. ***Parameter uncertainty***: DSA – int. cost had largest impact on ICER of Screen then supplement vs. NR. PSA – 100% probability of Screen then supplement being most cost-effective strategy at threshold of €20,000 (US$297292021 price). ***Scenarios***: Results robust to discount rates rising from 3 to 6%.	Hospitalisation cost only; Unclear intervention reach;^c^ Structural, external and internal validities assessed

*Abbreviations*: *CEA* Cost-effectiveness analysis, *CEAC* Cost-effectiveness acceptability curve, *CD* Community-dwelling, *CUA* Cost-utility analysis, *DSA* Deterministic sensitivity analysis, *ED* Emergency department, *HAM* Home assessment and modification, *ICER* Incremental cost-effectiveness ratio, *int.* Intervention, *LTC* Long-term care admission, *MA fall* Fall requiring medical attention, *NR* Non-receipt of modelled intervention(s), *OMAS* Ontario Medical Advisory Secretariat, *pharma.* Pharmaceuticals, *PSA* Probabilistic sensitivity analysis, *QALY* Quality-adjusted life year, *rehab.* Rehabilitation, *RCN* Royal College of Nursing, *ROI* Return on investment, *UC* Usual care, *UI* Uncertainty interval

^a^See Table 2 for study references; parenthesised number refers to the number of models included in the table

^b^All monetary units are converted to US$ in year 2021 using the average consumer price index (CPI) between the original year of reported currency to 2019 (most recent year for CPI data) [47] in the country of study and purchasing power parity (PPP) rate between the original currency and US$ in year 2020 (most recent PPP data) [48]

^c^Intervention reach refers to the number/proportion of persons receiving the intervention. It is a function of intervention’s *normative* reach defined by its eligibility criteria and targeting strategy and its *implementation* reach determined by the level of implementation (e.g., uptake and adherence) within the eligible population

^d^The study does not mention how falls risk progressed with age in the absence of falls incidence (which has a separate model state). Markov model should incorporate tunnel states to allow for secular risk progression, but this is not stated or graphically illustrated

^e^The study evaluated counterfactual scenarios where Maori/men had equal life expectancy as non-Maori/women and found that subgroup ICERs became similar (Maori/non-Maori only in Wilson (2017) [92]). This does not estimate the equity-efficiency trade-off (efficiency cost) from Maori/men being prioritised for intervention under the actual circumstance of lower life expectancy

^f^Without individual identifiers, multiple falls experienced by the same person are counted as multiple fallers

^g^Background health utility level should vary in line with changes to underlying health status which are influenced by age and changes in comorbidities and frailty affected by falls

^h^Societal costs incur different opportunity cost to public sector costs. The cost-effectiveness threshold should be tiered or weighted to capture the differing opportunity costs across sectors

^i^Multifactorial intervention included tailored education, group balance exercises, Tai Chi, other physical activities and HAM. Environmental intervention included neighbourhood hazard removal and housing reconstruction

^j^The study incorporated cost of added life-years which was estimated as the consumption minus production level (i.e., net consumption) that varied by age group. The outcome changed from dominance to ICER of US$23,715 per QALY

^k^The study estimated the proportion of target population who would be eligible for each of the interventions according to the prevalence of falls risk factors that defined eligibility: exercise for mobile older without disability (65.8%); HAM for frail older with disability (16.9%); vitamin D for women with fracture risk factors (52.9% of female); psychotropics withdrawal for psychotropic users (11.8%); and gait stabilizers for mobile seniors without disability (65.8%). However, the falls risk in the model was determined only by age, sex and MA falls history. Hence, different intervention subgroups had similar falls risk despite contrasting risk factor profiles

^l^HAM targeting subgroup with history of MA fall

^m^HAM targeting subgroup without history of MA fall

All models that conducted CUA except Eldridge [63] produced ICER for at least one intervention relative to no intervention or usual care that can be deemed cost-effective under the NICE threshold. In the order of increasing ICER values, these interventions were:

- Johansson [73]: Combined multifactorial and environmental intervention for age 65+
- RCN [34]: Multifactorial intervention for high-risk group aged 60+
- Nshimyumukiza [80]: General physical activity promotion among women (without population-level fracture risk screening) aged 65+
- Honkanen [70]: Hip protector use for women aged 80 or 85 at baseline and men aged 85
- Wilson [92]: HAM for state-level population with or without MA falls history aged 65+
- Deverall [62]: Home exercise and peer-led group exercise for age 65+
- Pega [82]: HAM for national population with or without MA falls history aged 65+
- Zarca [94]: Vitamin D screening followed by supplementation for age 65+
- RCN [34]: Exercise for high-risk group aged 60+
- Farag [64]: Non-specific intervention of US$587 per-participant cost and 25% reduction in risk for age 65+
- Church [58]: Tai Chi for age 65+

Given these interventions, a key decisional factor is their aggregate impacts determined by their reaches. The combined intervention in Johansson [73] arguably has the greatest reach since it sets no risk-based eligibility criteria for multifactorial intervention, and its environmental components reduce risk factors independently of older people’s demand. Therefore, the decision-maker should consult stakeholders to determine the local scalability of the combined intervention.

Consideration of aggregate impacts likewise shows that HAM in Pega [82] and Wilson [92] should not be targeted at those with MA falls history unless there are significant budget or capacity constraints: the universal approach remains highly cost-effective and produces greater aggregate impact than the targeted approach. In Honkanen [70], the sharp disparity in cost-per-unit ratios across baseline age subgroups justifies the age-based targeting of hip protector use, but the lack of age-related risk progression in the Markov cohort model may have disadvantaged the younger groups. In Zarca [94], the different reaches of alternative strategies were not clearly specified, with outcomes (incremental costs and QALYs) being reported at per-participant rates only. Universal vitamin D supplementation generated less favourable per-participant outcomes than targeted supplementation but may have produced greater aggregate benefits, especially when the model allows individuals with sufficient baseline vitamin D level (75 nmol/L) to derive fracture risk reductions from further supplementation (up to 105 nmol/L). The study’s conclusion that targeted strategies are preferable to universal supplementation would be misleading if only per-participant outcomes were compared.

Eldridge [63] demonstrated how considerations of cost-per-unit ratio and aggregate impact are in reality closely linked. The cost-effectiveness of the multi-pathway intervention was poor with 40% probability of it being cost-effective vs. usual care under the threshold of US$41,900 (£30,000) per QALY (ICER point estimate was not reported). The study attributed this to low intervention uptake (6.5% among eligible population) which interacted with the substantial fixed intervention costs to worsen the cost-effectiveness. Hence, the uptake rate was the key policy variable: the model estimated that 100% screening uptake would reduce the number of fallers by 11.3% over one year compared to 2.8% under the base case. The potential impact on the ICER was not reported but can be anticipated to be highly positive. The study recommended a health promotion campaign to increase the uptake; the decision-maker should likewise consider investments in auxiliary implementation strategies.

Regarding intervention impact on social inequities of health, Deverall [62], Pega [82], and Wilson [92] – presented subgroup results across ethnicity (Maori vs. non-Maori) and found higher ICERs and lower health gains for Maori. The HAM and exercise interventions hence *worsened* the health inequity between ethnic groups relative to usual care, and this finding may generalise to other settings with similar social disparities in health opportunities. The decision-maker could choose to permit this increase in health inequity or design an alternative strategy that generates an equal or greater health gain for the socially deprived group. The latter would likely introduce an equity-efficiency trade-off relative to the base case strategy which may be accepted or rejected by stakeholders based on their inequity aversion [96]. Such scenarios were not explored by the models. Yet they identified pre-existing life expectancy differentials between ethnic subgroups as the main cause of the inequitable impact: assigning non-Maori life expectancy on the Maori subgroup nearly eliminated the health gain differentials. This presents a rationale for commissioning interventions at earlier life stages to reduce the life expectancy differential.

### Methodological recommendations

Methodological recommendations are made based on accounting for falls epidemiology features, falls prevention intervention features, evaluation methods, and how evaluation outcomes are used to formulate commissioning recommendations.

#### Falls epidemiology features

1. Clearly state the type and source of data used to characterise the baseline falls risk and discuss the strengths and limitations of choice.
2. Use appropriate methods to characterise recurrent falls, particularly for individual-transitioning models with annual cycles.
3. Maximise the range of falls risk factors modelled including those highlighted by NICE CG161 (falls history, fear of falling, home hazards, gait deficit, balance deficit, mobility impairment, visual impairment, cognitive impairment, urinary incontinence) [4] and multivariate frailty [97]. Use individual-level data where available.
4. Maximise the range of falls health consequences modelled including the long-term impact on risks of mortality and health/functional decline.
5. For CUA, distinguish between acute and long-term impacts of fall-related events on health utility and discern whether assigning utility decrement (absolute or proportional) or level is more appropriate for each impact.
6. Maximise the range of fall-related economic consequences modelled including comorbidity care costs associated with the long-term mortality and morbidity impacts of falls. Where data permit, incorporate all-cause care costs which capture the full care consequences of falls, while also reporting fall-related care costs [32].

#### Falls prevention intervention features

1. Clearly describe the comparator(s); refrain from using the terms ‘usual care’ and ‘no intervention’ interchangeably and describe the usual care received [32].
2. Clearly state the access pathway(s) – reactive, proactive or self-referred – for intervention(s) and describe the mechanisms facilitating access (e.g., marketing for self-referred pathway).
3. Use appropriate methods for modelling the falls risk screening process to identify subgroups within target populations or specific patient groups serving as target populations. Resource-use associated with screening should be appropriately characterised and costed.
4. Maximise the granularity of intervention resources incorporated and costed, including auxiliary implementation resources (see expert guideline for resource types [32]). Refrain from translating fixed costs into per-participant rates to capture interaction with implementation level.
5. Ensure that the efficacy metric (i.e., RR or RaR) and fall type match the falls incidence metric (falls risk or rate) and type. Refrain from making assumptions on long-term efficacy duration without adequate evidence [32].
6. Where evidence is available, maximise the range of health effects of interventions modelled beyond falls prevention. These effects include intervention benefits on mortality and comorbidity reduction and intervention side-effects.

#### Evaluation methods

1. Assess and report the model’s structural, internal and external validities. Reduce the structural uncertainty prospectively by involving stakeholder and expert group in model development and retrospectively by evaluating scenarios associated with key structural assumptions.
2. Clearly state whether parameter variation represents DSA or scenario analysis. PSA should be conducted to assess the joint parameter uncertainty.

#### Evaluation outcomes to formulate commissioning recommendations

1. Report per-unit (e.g., ICERs) and aggregate (e.g., total incremental net monetary benefit) outcomes separately [32]. Use aggregate outcomes to compare cost-effective interventions (or combinations of interventions) of different target population sizes (normative reaches), and to evaluate implementation strategies (altering implementation reaches).
2. Evaluate the intervention impact on social inequities of health and use evaluative frameworks – such as distributional cost-effectiveness analysis (DCEA) [96] – that can incorporate the strength of decision-maker’s inequity aversion when comparing alternative intervention strategies with differing impacts on total health gain and social inequities of health.

### Commissioning recommendations

Our commissioning recommendations for the general older population over the lifetime horizon are:

1. Decision-makers should examine the transferability and feasible reach of the following seven interventions in local settings within their budget and capacity constraints: (i) combined multifactorial and environmental intervention for age 65+; (ii) general physical activity promotion for women aged 65+; (iii) hip protectors for women aged 80+ and men aged 85+; (iv) home or peer-led group exercise for age 65+; (v) HAM for persons with or without MA falls history aged 65+; (vi) targeted vitamin D supplementation for age 65+; and (vii) Tai Chi for age 65 + .
2. Where significant fixed cost investments are required, auxiliary implementation strategies should be planned to achieve adequate cost-effectiveness and aggregate impact.
3. There is some evidence that exercise and HAM exacerbate existing health inequity across social subgroups. The decision-maker should consider supplementary strategies that prioritise intervention access for the local socially marginalised groups and/or increase their upstream health opportunities. The potential equity-efficiency trade-off should be quantified.
4. Results for interventions (i), (ii) and (vi) are the most credible since they are produced by validated models; (ii) and (vi) are also from individual-level simulations that incorporated age-related progression in fracture risk. The decision-maker could also commission a *de novo*, validated model that addresses the methodological challenges and is suited to the local context.

## Discussion

This systematic review identified 46 decision models of community-based falls prevention interventions, applied a checklist specifically designed for falls prevention economic evaluations, and synthesised the modelling methods for key features of falls epidemiology, falls prevention intervention and evaluation. It also formulated (i) 16 methodological recommendations for future model development and (ii) four commissioning recommendations around seven interventions found to be cost-effective in general population, lifetime models.

A key issue in the use of reviewed model outcomes for commissioning is the generalisability or transferability of the said outcomes to the local decision-making context. The commissioning recommendations in this review adopted the cost-effectiveness threshold recommended by NICE for England and Wales [49]; decision-makers in other national settings should follow the recommendations of their respective HTA guiding bodies, such as the Pharmaceutical Benefits Advisory Committee (PBAC) in Australia [98]. As noted in the first commissioning recommendation, commissioners should actively verify the transferability and the local feasible reach of the interventions being considered. This should involve active collaboration with local stakeholders in interpreting the model evidence and even adapting an existing model to maximise its local relevance [31]. Frameworks such as the Context and Implementation of Complex Interventions (CICI) can systematically examine the influence of local context (e.g., regulation/policy on age-based targeting [99]) and supply conditions (e.g., capacity constraints) on HTA outcomes and thereby assist the assessment of transferability [100, 101]. The subgroup delineator of equity relevance would also be locally specific, thus affecting the generalisability of the health equity impacts of previously modelled interventions. Further methodological features influence the generalisability, such as the evidence sources for baseline falls risk and intervention efficacy. This strengthens the rationale for the review to conduct a thorough methodological appraisal of models before formulating commissioning recommendations.

For decision-makers in England and Wales, the commissioning recommendations can be compared to those made by the existing falls prevention clinical guideline, CG161 [4]. The guideline prioritises the proactive pathway involving falls risk screening followed by multifactorial intervention. This is supported by RCN [34] findings (which informed CG161) that multifactorial intervention for high-risk individuals dominates no intervention. By contrast, Eldridge [63] found proactive multifactorial intervention to generate an unfavourable cost-effectiveness profile, likely due to the low pathway uptake that increased the per-participant cost. Hence, the simplistic costing assumptions in RCN [34] may have overestimated the cost-effectiveness of proactive multifactorial intervention. Meanwhile, the consultation of local services in Eldridge [63] to understand uptake and costs makes their result more credible. Yet multifactorial intervention for all risk groups combined with environmental modification (costed using primary data) yielded positive results in Johansson [73]. A similar intersectoral intervention was found to be cost-effective over a five-year horizon in Beard [53]. Both models do not explore to what extent the positive results can be attributed to the multifactorial rather than the environmental component. This warrants further modelling work that incorporates both components and yet isolates their respective impacts. Until then, the decision-maker should commission the CG161-recommended multifactorial intervention but supplement this with environmental modifications. Specifically, local stakeholders should be consulted to verify whether the intersectoral initiatives in Johansson [73] and Beard [53] can be replicated in their local context. In addition, auxiliary implementation strategies should be planned with stakeholders to avoid the unfavourable outcomes seen in Eldridge [63]; this would involve understanding the facilitators and barriers to implementation from older persons’ and professionals’ perspectives [102–104].

Interestingly, there were several positive cost-effectiveness outcomes for interventions that had *not* been recommended by CG161. First, CG161 does not recommend unsupervised brisk walking for women and untargeted group exercise; although the 2019 surveillance for CG161 update [105] recommends physical activity promotion in line with the 2019 UK Chief Medical Officers’ physical activities guidelines [25]. By contrast, Nshimyumukiza [80] found general physical activity (including daily walking) for inactive older women to dominate no intervention. Second, CG161 does not recommend vitamin D supplementation even for those with vitamin D insufficiency or deficiency due to insufficient clinical evidence; by contrast, Zarca [94] found targeted vitamin D supplementation to be highly cost-effective relative to no intervention. Likewise, hip protectors and CBT were not recommended by CG161 but found to be cost-effective in Honkanen [70] and Tannenbaum [89], respectively. These divergences reflect the difference in the underlying approach to statistics and probability. For example, CG161 is primarily informed by RCT evidence that takes the frequentist approach of drawing random samples to test the likelihoods of alternative hypotheses representing the true (fixed) state of the world; while decision models take the Bayesian approach of estimating the expected state of the world based on prior beliefs and diverse types of data (p. 323) [26]. The latter arguably better reflects the type of uncertainty faced by decision-makers and should be prioritised in commissioning considerations over clinical evidence alone, provided that the models are methodologically robust, validated and assessed for the impact of parameter uncertainty on expected outcomes [106].

This imbues additional importance to thorough methodological appraisal of models – conducted in this review using two complementary approaches: checklist application and narrative synthesis. The falls-specific rather than generic checklist helped identify features unique to falls and falls prevention [32], including whether the study gave the definition of a fall – only 19 (41.3%) fully did – and whether the intervention(s) was classified as single, multiple-component or multifactorial – only 15 (32.6%) did. However, the checklist – designed for both models and non-modelling evaluations – did not consider important modelling features such as baseline risk characterisation and model validation which are included in the HTA model quality checklist [45]. Moreover, modelling features typically involve methodological nuances that cannot be summarised in ordinal scores. It is also unclear whether the unweighted sum of item scores accurately captures the methodological quality of models given the study-specific combination of methodological caveats; although of the 12 general population, lifetime models used to inform commissioning, the three that had been most thoroughly validated also had the highest checklist scores [73, 80, 94]. This illustrates the importance of supplementing the checklist application with narrative synthesis. The latter was more comprehensive in this systematic review than those conducted by previous systematic reviews in this topic area [33].

The checklist application nevertheless identified the most prevalent reporting and methodological limitations across models. The most prevalent issue was the non-incorporation of all-cause care costs as the main analysis cost outcome, with fall-related costs being reported in sensitivity analysis [32]. Older persons typically occupy a position on a continuous spectrum of frailty rather than one of binary healthy vs. diseased states [107–109]. A disease or fall incidence would shift the position on the spectrum and thereby incur myriad care costs only indirectly associated with the initial event [10, 110]; incorporating all-cause care costs helps capture these impacts as well as the wider benefits of interventions beyond falls prevention. It is also consistent with the aim of wider geriatric health policies such as person-centred integrated care that emphasise holistic outcomes [41, 111, 112]. Yet only four models incorporated all-cause costs [51, 52, 86, 87]; one of them even perceived all-cause costing as a limitation, compelled by lack of condition delineators in the routine data used [51]. The four also did not separately report fall-related costs, introducing difficulties in determining whether the cost reduction can be attributed to falls prevention *per se* rather than to wider intervention benefits. A major barrier is the lack of data on all-cause care consequences of falls, with costing studies focusing on fall-specific costs [17, 18]. Indeed, all four models that incorporated all-cause costs relied on primary data.

An alternative, more feasible approach is to incorporate comorbidity care costs (e.g., costs of added life-years and costs of dying) associated with background health status and life expectancy. The proximity of these costs to intervention effect makes their inclusion particularly important for geriatric populations [39]. The inclusion of health utilities to depict the transition in background health status also demands the inclusion of matching costs [26]. The higher cost of dying for younger age at death [113, 114] – as incorporated in the four BODE3 models – would improve the cost-effectiveness of interventions preventing premature mortality (e.g., of those below the average life expectancy). Yet comorbidity care costs were included by only six models (see Table A6); data availability may again be the barrier. The six models also included *all-cause* background costs and did not subtract fall-related costs, meaning that the latter are double-counted. Moreover, all except Honkanen [70] stratified the background costs by age and sex alone, meaning that the costs are influenced only by falls affecting mortality and not by those affecting morbidity and functional status. In all, further research is warranted to incorporate comorbidity costs in falls prevention modelling. A potential approach is to estimate the association between falls and multivariate frailty index, which in turn would determine all-cause care costs and subsequent falls risk [97, 107, 115].

The next two prevalent issues were reporting aggregate outcomes and detailing the comparator scenario. The importance of comparing aggregate outcomes was discussed under section 4.4. In brief, models should assist decision-makers in estimating the aggregate, population-level impact of interventions as recommended by the NICE HTA guidelines (see points 5.12.3 to 5.12.7) [49]. This is also highlighted in the NICE guideline for local public health service commissioning which shows how intervention rankings change by the metric chosen, including incremental cost per QALY, intervention reach (i.e., aggregate impact) and inequality impact (p. 118–122) [116]. Regarding the comparator scenario, this should closely resemble current practice in the local setting [32, 117]. To this end, it should be noted that current practice in most settings is perhaps not the total absence of falls prevention, compared to under-implementation of existing clinical guidelines [102]. Likewise, the most relevant intervention scenario is perhaps not the provision of new interventions, compared to upscaling of existing capacity and improving fidelity to recommended practice. With assistance from local stakeholders, future models should pay greater attention to the features of current practice in the decision-making setting and be more specific in the causal mechanisms being altered under intervention scenarios. As done in Eldridge [63], consultation of local services can assess current referral pathways and demand levels, and detail component-specific strategies (e.g., health promotion campaign to increase screening uptake). The greater attention would also facilitate the assessment of the transferability of specific model outcomes to different decision-making settings.

### Future research: systematic reviews and decision modelling

The results of this review offer research directions for both future systematic reviews and models. First, the methodological challenges associated with falls prevention modelling are generalisable to other geriatric syndromes including delirium, frailty and urinary incontinence [3] and other geriatric public health interventions [36, 39, 109]. Commissioners and modellers interested in these areas would benefit from a systematic review using similar review methodology, namely detailed methodological appraisal around epidemiology, intervention, evaluation methods and consideration of evaluation outcomes beyond cost-per-unit ratios. Secondly, there is an acute scarcity of models set in the developing country context [40]. This context is likely characterised by more pronounced capacity constraints which in turn requires modelling techniques that can account for them including discrete event simulation [95] and constrained optimisation [118].

### Strengths and limitations

This systematic review is a comprehensive review of community-based falls prevention models. It includes 26 models unidentified by previous systematic reviews in this area [33]. It also provides a more detailed methodological appraisal than previous systematic reviews using both checklist application and narrative synthesis. The appraisal results arranged by topic areas should facilitate the conceptualisation and cross-validation of future models. Another strength is the consideration of a broad range of outcomes for commissioning recommendations: previous reviews focused primarily on cost-per-unit ratios [33].

This review nevertheless has several limitations. First, appraisal of previous models was limited to what the studies reported. This presented difficulties in certain areas (e.g., whether Markov model incorporated tunnel states); contacting the study authors for enquiry would have reduced ambiguity. Secondly, in several ROI analyses (e.g., [56, 59, 72]), it was unclear whether the analyses constituted full comparative economic evaluations or non-comparative service evaluations (i.e., a partial economic evaluation) [119]. Clearer description of the evaluation aim and detailed parameterisation of the comparator scenario would facilitate future distinctions. Thirdly, unlike previous reviews, this review excluded non-modelling evaluations which still offer useful information for commissioning despite their short time horizons [40]; however, their incommensurable methodological features relative to models would have over-extended the boundary of appraisal. Fourthly, the review did not test for possible publication bias; there is hence a risk that favourable cost-effectiveness results are over-represented. Finally, the commissioning recommendations were based solely on general population, lifetime models, even though non-general population and/or non-lifetime models still offer useful information to decision-makers. Models evaluating alternative time horizons found that longer horizons improved cost-effectiveness [60, 86, 87, 89, 92], meaning that current commissioning recommendations are over-prescriptive for decision-makers with shorter horizons. Such decision-makers are invited to utilise the outcomes gathered in Table A11 in Supplementary Materials alongside the synthesised methodological features to reach appropriate commissioning decisions.

## Conclusions

There is model-based evidence that combined multifactorial and environmental intervention, general physical activity promotion for women, and targeted vitamin D supplementation are cost-effective relative to no intervention. Narrative synthesis found significant heterogeneity in modelling methods across falls epidemiology, falls prevention intervention and evaluation. This systematic review provides comprehensive catalogues of modelling methods and evaluation results for community-based falls prevention which should inform model selection and development, and commissioning strategies.

## Supplementary Information

**Additional file 1.** Supplementary Materials document for PRISMA checklist and Tables A1-A11.

## Data Availability

The Excel file containing extracted data is available from the corresponding author upon reasonable request.

## Abbreviations

AC: All-Cause
BMD: Bone Mass Density
BODE3: Burden of Disease Epidemiology, Equity & Cost-Effectiveness
CBA: Cost-Benefit Analysis
CBT: Cognitive Behavioural Therapy
CD: Community-Dwelling
CEA: Cost-Effectiveness Analysis
CEAC: Cost-Effectiveness Acceptability Curve
CEAF: Cost-Effectiveness Acceptability Frontier
CG: Clinical Guideline
Com.: Composite
CPI: Consumer Price Index
CSP: Chartered Society of Physiotherapy
CUA: Cost-Utility Analysis
CVD: Cardiovascular Disease
DCEA: Distributional Cost-Effectiveness Analysis
DSA: Deterministic Sensitivity Analysis
DT: Decision Tree
ED: Emergency Department
Exp.: Expedited
Extrapol.: Extrapolated
FRA: Falls Risk Assessment
FRAT: Falls Risk Assessment Tool
FRID: Fall-Risk-Increasing Drug
FRS: Falls Risk Screening
HAM: Home Assessment and Modification
HTA: Health Technology Assessment
ICER: Incremental Cost-Effectiveness Ratio
Int.: Intervention
LTC: Long-Term Care
MA fall: Fall requiring Medication Attention
MC: Multiple-Component
Med.: Medication
Met-An.: Meta-Analysis
MF: Multifactorial
MoB/VLL: Matter of Balance Lay-Led Version
MRA: Multifactorial Risk Assessment only
N/A: Not Applicable
NICE: National Institute for Health and Care Excellence
NR: Non-Receipt of modelled intervention(s)
NSW: New South Wales
OMAS: Ontario Medical Advisory Secretariat
OR: Odds Ratio
PBAC: Pharmaceutical Benefits Advisory Committee
Pharma.: Pharmaceuticals
PHE: Public Health England
PIP: Potentially Inappropriately Prescribed
PPI: Purchasing Power Parity
PPIn: Proton Pump Inhibitor
PRISMA: Preferred Reporting Items for Systematic Reviews and Meta-Analyses
PSA: Probabilistic Sensitivity Analysis
QALY: Quality-Adjusted Life Year
QTUG: Quantitative Timed-Up-and-Go
RaR: Rate Ratio
RCN: Royal College of Nursing
RCT: Randomised Controlled Trial
Rehab.: Rehabilitation
ROI: Return on Investment
RR: Relative Risk
SR: Systematic Review
TUG: Timed-Up-and-Go
UC: Usual Care
UI: Uncertainty Interval
Vit. D: Vitamin D
