# Supplementary material for: Economic models of community-based falls prevention: a systematic review with subsequent commissioning and methodological recommendations
Source: BMC Health Serv Res. 2022 Mar 7;22:316. doi: 10.1186/s12913-022-07647-6 (PMC8902781; doi:10.1186/s12913-022-07647-6)
Supplement: Supplementary file 1 — Additional file 1. Supplementary Materials document for PRISMA checklist and Tables A1-A11. [file 12913_2022_7647_MOESM1_ESM.docx]

# Economic models of community-based falls prevention: a systematic review with subsequent commissioning and methodological recommendations

**Supplementary Materials**

**Authors:**

Mr Joseph Kwon^1*^, [jkwon6@sheffield.ac.uk](mailto:jkwon6@sheffield.ac.uk); ORCID 0000-0002-2860-7280

Dr Hazel Squires^1^, [h.squires@sheffield.ac.uk](mailto:h.squires@sheffield.ac.uk); ORCID 0000-0001-8467-0471

Dr Matthew Franklin^1^, [matt.franklin@sheffield.ac.uk](mailto:matt.franklin@sheffield.ac.uk); ORCID 0000-0002-2776-4014

Ms Yujin Lee^2^, [Yujin.Lee.1@warwick.ac.uk](mailto:Yujin.Lee.1@warwick.ac.uk); ORCID 0000-0003-0450-9667

Professor Tracey Young^1^, [t.a.young@sheffield.ac.uk](mailto:t.a.young@sheffield.ac.uk); ORCID 0000-0002-0754-7223

^1^ School of Health and Related Research, University of Sheffield, Regent Court (ScHARR), 30 Regent Street, Sheffield, England, S1 4DA

^2^ Warwick Medical School, University of Warwick, Gibbet Hill Road, Coventry, England, CV4 7AL

* Corresponding author

# PRISMA 2020 Checklist

| **Section and Topic** | **Item #** | **Checklist item** | **Location where item is reported** |
| --- | --- | --- | --- |
| **TITLE** | | |  |
| Title | 1 | Identify the report as a systematic review. | Title |
| **ABSTRACT** | | |  |
| Abstract | 2 | See the PRISMA 2020 for Abstracts checklist. | Abstract checklist items all met |
| **INTRODUCTION** | | |  |
| Rationale | 3 | Describe the rationale for the review in the context of existing knowledge. | Section 1, first five paragraphs |
| Objectives | 4 | Provide an explicit statement of the objective(s) or question(s) the review addresses. | Section 1, last paragraph |
| **METHODS** | | |  |
| Eligibility criteria | 5 | Specify the inclusion and exclusion criteria for the review and how studies were grouped for the syntheses. | Section 2.1, 3^rd^ and 4^th^ paragraphs |
| Information sources | 6 | Specify all databases, registers, websites, organisations, reference lists and other sources searched or consulted to identify studies. Specify the date when each source was last searched or consulted. | Section 2.1, 1^st^ paragraph; Supplementary Materials (SM), Database search strategies |
| Search strategy | 7 | Present the full search strategies for all databases, registers and websites, including any filters and limits used. | SM, Database search strategies |
| Selection process | 8 | Specify the methods used to decide whether a study met the inclusion criteria of the review, including how many reviewers screened each record and each report retrieved, whether they worked independently, and if applicable, details of automation tools used in the process. | Section 2.1, 2^nd^ paragraph |
| Data collection process | 9 | Specify the methods used to collect data from reports, including how many reviewers collected data from each report, whether they worked independently, any processes for obtaining or confirming data from study investigators, and if applicable, details of automation tools used in the process. | Section 2.2, 1^st^ paragraph |
| Data items | 10a | List and define all outcomes for which data were sought. Specify whether all results that were compatible with each outcome domain in each study were sought (e.g. for all measures, time points, analyses), and if not, the methods used to decide which results to collect. | Table 1 |
|  | 10b | List and define all other variables for which data were sought (e.g. participant and intervention characteristics, funding sources). Describe any assumptions made about any missing or unclear information. | No other variables sought |
| Study risk of bias assessment | 11 | Specify the methods used to assess risk of bias in the included studies, including details of the tool(s) used, how many reviewers assessed each study and whether they worked independently, and if applicable, details of automation tools used in the process. | Methodological appraisal: Section 2.2.1 and 2.2.2; SM, Table A2 |
| Effect measures | 12 | Specify for each outcome the effect measure(s) (e.g. risk ratio, mean difference) used in the synthesis or presentation of results. | Section 2.2.3 |
| Synthesis methods | 13a | Describe the processes used to decide which studies were eligible for each synthesis (e.g. tabulating the study intervention characteristics and comparing against the planned groups for each synthesis (item #5)). | Section 2.1, 3^rd^ and 4^th^ paragraphs |
|  | 13b | Describe any methods required to prepare the data for presentation or synthesis, such as handling of missing summary statistics, or data conversions. | Sections 2.2.1-2.2.3 |
|  | 13c | Describe any methods used to tabulate or visually display results of individual studies and syntheses. | Sections 2.2.1-2.2.3 |
|  | 13d | Describe any methods used to synthesize results and provide a rationale for the choice(s). If meta-analysis was performed, describe the model(s), method(s) to identify the presence and extent of statistical heterogeneity, and software package(s) used. | Sections 2.2.1-2.2.3 |
|  | 13e | Describe any methods used to explore possible causes of heterogeneity among study results (e.g. subgroup analysis, meta-regression). | Section 2.2.3: results from general population, lifetime models for commissioning |
|  | 13f | Describe any sensitivity analyses conducted to assess robustness of the synthesized results. | No sensitivity analysis |
| Reporting bias assessment | 14 | Describe any methods used to assess risk of bias due to missing results in a synthesis (arising from reporting biases). | No risk of bias analysis for synthesis |
| Certainty assessment | 15 | Describe any methods used to assess certainty (or confidence) in the body of evidence for an outcome. | No assessment of certainty |
| **RESULTS** | | |  |
| Study selection | 16a | Describe the results of the search and selection process, from the number of records identified in the search to the number of studies included in the review, ideally using a flow diagram. | Section 3.1; Figure 1 |
|  | 16b | Cite studies that might appear to meet the inclusion criteria, but which were excluded, and explain why they were excluded. | SM, Excluded studies at full text screening stage |
| Study characteristics | 17 | Cite each included study and present its characteristics. | Table 2 |
| Risk of bias in studies | 18 | Present assessments of risk of bias for each included study. | Methodological appraisal: Table 3; Sections 3.4 and 3.5 |
| Results of individual studies | 19 | For all outcomes, present, for each study: (a) summary statistics for each group (where appropriate) and (b) an effect estimate and its precision (e.g. confidence/credible interval), ideally using structured tables or plots. | Table 9; SM, Table A11 |
| Results of syntheses | 20a | For each synthesis, briefly summarise the characteristics and risk of bias among contributing studies. | No risk of bias analysis for synthesis |
|  | 20b | Present results of all statistical syntheses conducted. If meta-analysis was done, present for each the summary estimate and its precision (e.g. confidence/credible interval) and measures of statistical heterogeneity. If comparing groups, describe the direction of the effect. | No statistical synthesis |
|  | 20c | Present results of all investigations of possible causes of heterogeneity among study results. | Table 9 for general population, lifetime models; SM, Table A11 for non-general population and/or non-lifetime models |
|  | 20d | Present results of all sensitivity analyses conducted to assess the robustness of the synthesized results. | No sensitivity analysis |
| Reporting biases | 21 | Present assessments of risk of bias due to missing results (arising from reporting biases) for each synthesis assessed. | No risk of bias analysis for synthesis |
| Certainty of evidence | 22 | Present assessments of certainty (or confidence) in the body of evidence for each outcome assessed. | No assessment of certainty |
| **DISCUSSION** | | |  |
| Discussion | 23a | Provide a general interpretation of the results in the context of other evidence. | Section 4, 3^rd^ and 4^th^ paragraphs |
|  | 23b | Discuss any limitations of the evidence included in the review. | Section 4.2 |
|  | 23c | Discuss any limitations of the review processes used. | Section 4.2 |
|  | 23d | Discuss implications of the results for practice, policy, and future research. | Section 4, 2^nd^ paragraph; Section 4.1 |
| **OTHER INFORMATION** | | |  |
| Registration and protocol | 24a | Provide registration information for the review, including register name and registration number, or state that the review was not registered. | Section 2 |
|  | 24b | Indicate where the review protocol can be accessed, or state that a protocol was not prepared. | Section 2 |
|  | 24c | Describe and explain any amendments to information provided at registration or in the protocol. | No amendment made |
| Support | 25 | Describe sources of financial or non-financial support for the review, and the role of the funders or sponsors in the review. | Declarations, Funding |
| Competing interests | 26 | Declare any competing interests of review authors. | Declarations, Competing interests |
| Availability of data, code and other materials | 27 | Report which of the following are publicly available and where they can be found: template data collection forms; data extracted from included studies; data used for all analyses; analytic code; any other materials used in the review. | Declarations, Availability of data and materials |

# Database search strategies

| **Table A1.1** Medline search strategy | | | |
| --- | --- | --- | --- |
| Medline (Epub Ahead of Print, In-Process & Other Non-Indexed Citations, Ovid MEDLINE(R) Daily and Ovid MEDLINE(R)) – run on 7^th^ January 2021 | | | |
| **Theme** | **Type** | **Ref** | **Search Term** |
| Falls | Free text^1^ | 1 | (fall or falls or falling or faller* or fallen or fell or slip* or trip* or stumbl*).ti,ab. |
|  | MeSH | 2 | exp Accidental falls/ |
|  |  | 3 | 1 or 2 |
| Elderly and frailty | Free text^1^ | 4 | (old or older or senior* or elder* or aged or geriatric* or frail* or pensioner).ti,ab. |
|  | MeSH | 5 | exp Aged/ or Frailty/ |
|  |  | 6 | 4 or 5 |
| Economic evaluation | Free text^1^ | 7 | (economic or decision or Markov or cost-effectiveness or cost-utility or cost-benefit or cost or budget or expenditure or pric* or ROI).ti,ab. |
|  | MeSH | 8 | exp Models, economic/ or exp Economics/ or exp Economics, medical/ or exp Economics, nursing/ or exp Economics, pharmaceutical/ or exp Decision trees/ or exp Cost-benefit analysis/ or exp Costs and cost analysis/ or exp Budgets/ |
|  |  | 9 | 7 or 8 |
|  |  | 10 | 3 AND 6 AND 9 |
| Exclusions |  | 11 | Limit to Humans |
|  |  | 12 | (news or comment or editorial or letter or case reports).pt. or case report.ti. |
|  |  | 13 | 11 NOT 12 |
|  |  | 14 | Limit 13 to English |
|  |  | 15 | Limit 14 to 1^st^ January 2003 – 31^st^ December 2020 |
| **Abbreviation:** MeSH: medical subject heading  ^1^ Covering title and abstract. | | | |

| **Table A1.2** Embase search strategy | | | |
| --- | --- | --- | --- |
| Embase (source: OvidSP) – run on 7^th^ January 2021 | | | |
| **Theme** | **Type** | **Ref** | **Search Term** |
| Falls | Free text^1^ | 1 | (fall or falls or falling or faller* or fallen or fell or slip* or trip* or stumbl*).ti,ab,kw. |
|  | MeSH | 2 | exp falling/ OR exp fall risk/ |
|  |  | 3 | 1 or 2 |
| Older and frailty | Free text^1^ | 4 | (old or older or senior* or elder* or aged or geriatric* or frail* or pensioner).ti,ab. |
|  | MeSH | 5 | exp Aged/ or Frailty/ |
|  |  | 6 | 4 or 5 |
| Economic evaluation | Free text^1^ | 7 | (economic OR evaluation OR budget OR expenditure OR cost* OR ROI).ti,ab,kw. |
|  | MeSH | 8 | exp Health economics/ OR exp Economic model/ OR exp Economic evaluation/ OR exp Health care cost/ OR Pharmacoeconomics/ OR Cost effectiveness analysis/ OR Cost utility analysis/ OR Cost benefit analysis/ OR Cost minimization analysis/ OR Cost of illness/ |
|  |  | 9 | 7 or 8 |
|  |  | 10 | 3 AND 6 AND 9 |
| Exclusions |  | 11 | Limit to Humans |
|  |  | 12 | (news or comment or editorial or letter or case reports).pt. or case report.ti. |
|  |  | 13 | 11 NOT 12 |
|  |  | 14 | Limit to English |
|  |  | 15 | Exclude Medline journals |
|  |  | 16 | Limit to 1^st^ January 2003 – 31^st^ December 2020 |
| **Abbreviation:** MeSH: medical subject heading; Ref: reference  ^1^ Covering title and abstract. | | | |

| **Table A1.3** PubMed search strategy | | | |
| --- | --- | --- | --- |
| PubMed – run on 7^th^ January 2021 | | | |
| **Theme** | **Type** | **Ref** | **Search Term** |
| Falls | Free text^1^ | 1 | [tiab] fall or falls or falling or fallen or fell or slip or trip or stumbl* |
|  | MeSH | 2 | Accidental falls |
|  |  | 3 | 1 or 2 |
| Older and frailty | Free text^1^ | 4 | [tiab] old or older or senior or elder or aged or geriatric or frail or pensioner |
|  | MeSH | 5 | Aged or Frailty |
|  |  | 6 | 4 or 5 |
| Economic evaluation | Free text^1^ | 7 | [tiab] economic or budget or expenditure or evaluation or cost or markov or model or ROI |
|  | MeSH | 8 | Model, economic or Economics, medical or Economics, nursing or Economics, pharmaceutical or Costs and cost analysis or Costs and benefits or Budget or Markov chain or Decision analysis |
|  |  | 9 | 7 or 8 |
|  |  | 10 | 3 AND 6 AND 9 |
| Exclusions |  | 11 | Limit to Humans |
|  |  | 12 | (news or comment or editorial or letter or case reports).pt. or case report.ti. |
|  |  | 13 | 11 NOT 12 |
|  |  | 14 | Limit to English |
|  |  | 15 | Remove [Child: Birth-18 years], [Infant: 1-23 months] |
|  |  | 16 | Limit to 1^st^ January 2003 – 31^st^ December 2020 |
| **Abbreviation:** MeSH: medical subject heading; Ref: reference; tiab: titles and abstract  ^1^ Covering title and abstract. | | | |

| **Table A1.4** Cochrane Library (CSDR and CENTRAL) search strategy | | | |
| --- | --- | --- | --- |
| Cochrane Library (Cochrane Database of Systematic Reviews and CENTRAL trials registry) – run on 7^th^ January 2021 | | | |
| **Theme** | **Type** | **Ref** | **Search Term** |
| Falls | Free text^1^ | 1 | (fall or falls or falling or fallen or fell or slip or trip or stumbl*).ti,ab,kw. |
|  | MeSH | 2 | exp Accidental falls/ |
|  |  | 3 | 1 or 2 |
| Older and frailty | Free text^1^ | 4 | (old or older or senior or elder or aged or geriatric or frail or pensioner).ti,ab,kw. |
|  | MeSH | 5 | exp Aged/ or exp Aging/ or exp Frailty/ or exp Frail elderly/ |
|  |  | 6 | 4 or 5 |
| Economic evaluation | Free text^1^ | 7 | (economic OR evaluation OR budget OR expenditure OR cost* OR ROI).ti,ab,kw. |
|  | MeSH | 8 | exp Economics/ or exp Economics, nursing/ or exp Economics, pharmaceutical/ or exp Economics, medical/ or exp Models, Economic/ or exp Costs and cost analysis/ or exp Cost-benefit analysis/ or exp Cost of illness/ or exp Budgets/ or exp Health expenditures/ |
|  |  | 9 | 7 or 8 |
|  |  | 10 | 3 AND 6 AND 9 |
| Exclusions |  | 11 | Limit to 1^st^ January 2003 – 31^st^ December 2020 |
| **Abbreviation:** MeSH: medical subject heading; Ref: reference  ^1^ Covering title and abstract. | | | |

| **Table A1.5** EconLit search strategy | | | |
| --- | --- | --- | --- |
| EconLit (source: OvidSP) – run on 7^th^ January 2021 | | | |
| **Theme** | **Type** | **Ref** | **Search Term** |
| Falls | Free text^1^ | 1 | (fall or falls or falling or fallen or fell or slip or trip or stumbl*).ti,ab,kw. |
| Older and frailty | Free text^1^ | 2 | (old or older or senior or elder or aged or geriatric or frail or pensioner).ti,ab,kw. |
| Economic evaluation | Free text^1^ | 3 | (economic OR evaluation OR budget OR expenditure OR cost* OR ROI).ti,ab,kw. |
|  |  | 4 | 1 AND 2 AND 3 |
| Exclusions |  | 5 | Limit to English |
|  |  | 6 | Limit to 1^st^ January 2003 – 31^st^ December 2020 |
| **Abbreviation:** MeSH: medical subject heading; Ref: reference  ^1^ Covering title and abstract. | | | |

| **Table A1.6** CINAHL search strategy | | | |
| --- | --- | --- | --- |
| CINAHL (source: EBSCO) – run on 7^th^ January 2021 | | | |
| **Theme** | **Type** | **Ref** | **Search Term** |
| Falls | Free text^1^ | 1 | TI(fall or falls or falling or fallen or fell or slip or trip or stumbl*) |
|  | Free text | 2 | AB(fall or falls or falling or fallen or fell or slip or trip or stumbl*) |
|  | MeSH | 3 | MH(Accidental falls) |
|  |  | 4 | 1 or 2 or 3 |
| Older and frailty | Free text^1^ | 5 | TI(old or older or senior or elder or aged or geriatric or frail or pensioner) |
|  | Free text | 6 | AB(old or older or senior or elder or aged or geriatric or frail or pensioner) |
|  | MeSH | 7 | MH(Aged+) |
|  |  | 8 | 5 or 6 or 7 |
| Economic evaluation | Free text^1^ | 9 | TI(economic or evaluation or budget or expenditure or cost* or ROI) |
|  | Free text | 10 | AB(economic or evaluation or budget or expenditure or cost* or ROI) |
|  | MeSH | 11 | MH(Economics or Economic aspects of illness or Economics, pharmaceutical or Accidental falls economics) |
|  |  | 12 | 9 or 10 or 11 |
|  |  | 13 | 4 AND 8 AND 12 |
| Exclusions |  | 14 | Limit to Humans |
|  |  | 15 | PT(news or comment or editorial or letter or case reports) |
|  |  | 16 | 14 NOT 15 |
|  |  | 17 | Limit to English |
|  |  | 18 | Limit to Academic Journals (remove Dissertations, Magazines and CEUs) |
|  |  | 19 | Limit to 1^st^ January 2003 – 31^st^ December 2020 |
| **Abbreviation:** AB: abstract; MeSH: medical subject heading; Ref: reference; TI: title.  ^1^ Covering title and abstract. | | | |

| **Table A1.7** PsycInfo search strategy | | | |
| --- | --- | --- | --- |
| PsycInfo (source: OvidSP) – run on 7^th^ January 2021 | | | |
| **Theme** | **Type** | **Ref** | **Search Term** |
| Falls | Free text^1^ | 1 | (fall or falls or falling or fallen or fell or slip or trip or stumbl*).ti,ab. |
|  | MeSH | 2 | Falls/ |
|  |  | 3 | 1 or 2 |
| Older and frailty | Free text^1^ | 4 | (old or older or senior or elder or aged or geriatric or frail or pensioner).ti,ab. |
|  | MeSH | 5 | exp Aging/ or Geriatrics/ or Gerontology/ |
|  |  | 6 | 4 or 5 |
| Economic evaluation | Free text^1^ | 7 | (economic OR evaluation OR budget OR expenditure OR cost* OR ROI).ti,ab. |
|  | MeSH | 8 | exp Economics/ or exp “Costs and cost analysis”/ or “Resource allocation”/ or exp “Decision making”/ |
|  |  | 9 | 7 or 8 |
|  |  | 10 | 3 AND 6 AND 9 |
| Exclusions |  | 11 | Limit to Humans |
|  |  | 12 | (news or comment or editorial or letter or case reports).pt. or case report.ti. |
|  |  | 13 | 11 NOT 12 |
|  |  | 14 | Limit to English |
|  |  | 15 | Limit to 1^st^ January 2003 – 31^st^ December 2020 |
| **Abbreviation:** MeSH: medical subject heading; Ref: reference  ^1^ Covering title and abstract. | | | |

| **Table A1.8** ASSIA search strategy | | | |
| --- | --- | --- | --- |
| ASSIA (source: ProQuest) – run on 7^th^ January 2021 | | | |
| **Theme** | **Type** | **Ref** | **Search Term** |
| Falls | Free text^1^ | 1 | ti(fall or falls or falling or fallen or fell or slip or trip or stumbl*) |
|  | Free text | 2 | ab(fall or falls or falling or fallen or fell or slip or trip or stumbl*) |
|  | SH | 3 | Mainsubject.Exact(“falls” OR “accidental falls” OR “fall prone elderly people”) |
|  |  | 4 | 1 or 2 or 3 |
| Older and frailty | Free text^1^ | 5 | ti(old or older or senior or elder or aged or geriatric or frail or pensioner) |
|  | Free text | 6 | ab(old or older or senior or elder or aged or geriatric or frail or pensioner) |
|  | SH | 7 | Mainsubject.Exact(“aged, 80 & over” or “aged” or “frailty” or “frail elderly” or “frail elderly people” or “frail”) |
|  |  | 8 | 5 or 6 or 7 |
| Economic evaluation | Free text^1^ | 9 | ti(economic or evaluation or budget or expenditure or cost* or ROI) |
|  | Free text | 10 | ab(economic or evaluation or budget or expenditure or cost* or ROI) |
|  | SH | 11 | Mainsubject.Exact(“economic costs” or “economic aspects” or “economic analysis” or “economic impact” or “economic” or “budgets” or “benefit cost analysis” or “costs” or “cost-benefit analysis” or “cost effectiveness” or “costing” or “cost utility analysis” or “cost minimization analysis” or “cost benefit analysis” or “costs & cost analysis” or “cost analysis” or “cost of illness”) |
|  |  | 12 | 9 or 10 or 11 |
|  |  | 13 | 4 AND 8 AND 12 |
| Exclusions |  | 14 | Exclude commentary, news and editorial |
|  |  | 15 | Limit to English |
|  |  | 16 | Limit to 1^st^ January 2003 – 31^st^ December 2020 |
| **Abbreviation:** AB: abstract; Ref: reference; SH: subject heading; TI: title.  ^1^ Covering title and abstract. | | | |

**Other databases**

Search strategy for Centre for Reviews and Dissemination (CRD) – search run on 7^th^ January 2021

Title: “Fall” AND Limit publication year to 2003-2020

Result #: 61

Search strategy for Cost-Effectiveness Analysis (CEA) Registry – search run on 7^th^ January 2021

“Fall” as search term

Result #: 100 (only the most recent 100 hits available)

Search strategy for Physiotherapy Evidence Database (PEDro) – search run on 7^th^ January 2021

Title: “Fall” AND Limit publication year to 2003-2020

Result #: 226

**Grey literature**

The following sites were searched on 9^th^ January 2021 with term “Falls prevention”

- Age UK: <https://www.ageuk.org.uk/>
- Chartered Society of Physiotherapy: <https://www.csp.org.uk/>
- College of Occupational Therapy: <https://www.rcot.co.uk/>
- Department of Health: <https://www.gov.uk/government/organisations/department-of-health-and-social-care>
- Royal College of Nursing: <https://www.rcn.org.uk/>

# Checklist for assessing quality of falls prevention economic models

Table A2 shows the 32 items contained in checklist applied to studies included in this systematic review. The items are drawn from a checklist developed by an international group of falls prevention experts [1]. All items are drawn from Table 1 of the guideline document which displays the checklist [1]. Final column of Table A2 details the original numbering of the items. Each study is given a score of 1 if deemed to have followed the item recommendation fully, 0.5 if partially and 0 if not followed. The total potential score is hence 32.

| **Table A2.** Items for model quality assessment drawn from expert-validated checklist for conducting and reporting economic evaluation of falls prevention interventions. | | |
| --- | --- | --- |
| **#** | **Item^1,2^** | **Reference in guideline [1]** |
| **Define the type of study and the main objective(s)** | | |
| 1 | State whether a cost-effectiveness, cost-utility, or cost-benefit analysis and state the main outcomes of the analysis | Table 1, Item 1.1 |
| 2 | State whether carried out as part of a clinical trial or a model | Table 1, Item 1.2 |
| 3 | State the aim of the economic evaluation | Table 1, Item 1.3 |
| 4 | State the viewpoint [perspective] of the analysis and justify choice of viewpoint.^3^ | Table 1, Item 1.4 |
| **Describe competing alternatives** | | |
| 5 | Describe the intervention(s): (1) who delivered the intervention(s); (2) the components; (3) staff training; (4) how and where it was delivered; (5) frequency and dose; (6) whether sample in intervention study is similar to model population; (7) whether method of recruitment in intervention study is similar to intervention access method in model; (8) whether inclusion and exclusion criteria in intervention study are similar to intervention eligibility criteria in model.^4^ | Table 1, Item 2.1 |
| 6 | Classify the intervention(s) as single, multiple or multifactorial | Table 1, Item 2.2 |
| 7 | Include the justification for the intervention(s) and the comparator.^5^ | Table 1, Item 2.3 |
| 8 | Justify rationale for either including or excluding a “do-nothing” alternative.^5^ | Table 1, Item 2.4 |
| **Describe the method used to establish effectiveness** | | |
| 9 | State the source of the estimate for the effectiveness used: e.g., randomized controlled trial, systematic review | Table 1, Item 3.1 |
| 10 | State the definition of a fall used | Table 1, Item 3.2 |
| 11 | State the definition of a fall injury used.^6^ | Table 1, Item 3.4 |
| 12 | Provide the total number of falls (injuries) in each comparison group | Table 1, Item 3.5 |
| 13 | [Incorporate] uncertainty surrounding the effectiveness estimate | Table 1, Item 3.6 |
| 14 | If effectiveness is measured using a quality of life outcome such as QALYs, describe the method used for estimating QALY values | Table 1, Item 3.7 |
| **Identify all relevant costs and consequences for each alternative and comparator evaluated** | | |
| 15 | Identify all relevant total health resource utilisation costs for each alternative and comparator evaluated. Use total health resource utilisation costs for base case analysis. | Table 1, Item 4.1 |
| 16 | Identify all relevant fall-related costs and consequences for each alternative and comparator evaluated. Use fall-related costs for sensitivity analysis. | Table 1, Item 4.2 |
| **Ensure costs and consequences are measured accurately and in appropriate units** | | |
| 17 | Provide the units used for all cost items and sources for identifying these items | Table 1, Item 5.2 |
| 18 | Define fall-related costs as those incurred directly as a result of the fall. Provide the definition used for defining cost items as fall-related | Table 1, Item 5.3 |
| **Value costs and consequences credibly** | | |
| 19 | State the year and currency that costs were collected | Table 1, Item 6.1 |
| 20 | Use actual costs or validated methods to value each cost item if available | Table 1, Item 6.2 |
| 21 | Report total health resource utilisation costs, fall-related healthcare costs, and intervention costs separately. Report these costs both as a total and mean value broken down by group. | Table 1, Item 6.3 |
| **Costs and consequences should be adjusted for differential timing** | | |
| 22 | State and justify the time horizon over which costs and consequences were collected | Table 1, Item 7.1 |
| 23 | If costs were collected over a period of more than 1 year, use the recommended discount rate | Table 1, Item 7.2 |
| 24 | The effect of the intervention on the number of falls after completion of the trial should not be estimated or modelled as there are not adequate data available to estimate the future risk or cost of falls accurately | Table 1, Item 7.3 |
| 25 | If appropriate data permit, model the lifetime costs and consequences using a Markov model or discrete event simulation | Table 1, Item 7.4 |
| **Perform an incremental analysis of costs and consequences for all alternatives** | | |
| 26 | [Where natural unit of falls are used as health outcome under CEA,] report the ICER in three ways: (1) incremental cost per fall prevented; (2) incremental cost per unit decrease in falls per person-year (falls rate); and (3) incremental cost per unit decrease in mean number of falls per person | Table 1, Item 8.1 |
| 27 | Report all elements of incremental cost-effectiveness ratios (e.g., incremental costs, QALYs, total number of falls) separately for each group (preferably in a table). Avoid merely stating that one intervention “dominated” an alternative. | Table 1, Item 8.2 |
| **Identify key parameters and assumptions that may lead to different conclusions from the incremental analysis** | | |
| 28 | Estimate uncertainty for costs and consequences using comprehensive one-way sensitivity analyses and probabilistic sensitivity analyses | Table 1, Item 9.1 |
| **Present and discuss results from base case and sensitivity analyses** | | |
| 29 | Report key assumptions and values that substantially affected the estimates for costs and health outcomes | Table 1, Item 10.1 |
| 30 | Include a discussion of the assumptions and values of cost items and measures of effectiveness incorporated in the point estimates of cost-effectiveness or cost-utility outcomes | Table 1, Item 10.2 |
| 31 | Include a discussion of issues related to implementation of the intervention(s) – e.g., generalizability, feasibility, alternative settings, relevant ethical issues | Table 1, Item 10.3 |
| 32 | Discuss how the economic evaluation will inform health policy. | Table 1, Item 10.4 |
| **Abbreviation:** CEA: cost-effectiveness analysis; HRQoL: health-related quality of life; ICER: incremental cost-effectiveness ratio; ProFaNE: Prevention of Falls Network Europe; QALY: quality-adjusted life year.  ^1^ Each study is given a score of 1 for each item if it is deemed to have followed the recommendation sufficiently, 0.5 if followed sub-optimally and 0 if not followed. The maximum potential score is 32.  ^2^ The following items from the original checklist were excluded because they were deemed less relevant to the decision modelling context [1]: Item 3.3 concerning primary collection of falls data in trials; Item 3.8 concerning trial sample size calculation; Item 5.1 concerning primary collection of cost data in trials; Item 5.4 concerning methods for handling missing cost data; Item 7.5 concerning methods for costing resource items without decision modelling; Item 8.3 concerning methods for handling missing HRQoL and falls data; Item 8.4 concerning methods for identifying biases due to non-random missing data; Item 8.5 concerning adjusting the ICER for baseline HRQoL; Item 9.2 concerning estimation of intervention efficacy; and Item 9.3 concerning tests for statistical significance in between-group differences in outcomes.  ^3^ The original checklist adds: “A societal perspective is regarded as most comprehensive; however, a funder or provider perspective may be more appropriate depending on the research question” [1]. Therefore, unless justifying reasons are given, models which did not employ the societal perspective are given the score of 0.5.  ^4^ The original checklist recommends that the study describe the components, staff training, how and where it was delivered, frequency and dose, *the sample receiving the intervention, the method of recruitment and inclusion and exclusion criteria*. The latter italicized features are less relevant to decision models that infrequently conduct primary sampling and participant recruitment. They are hence adapted to address the issues of whether the external intervention study evidence suit the model’s population and intervention eligibility and access criteria. Out of 8 components, models that incorporated 6-8 are given a score of 1; those that incorporated 3-5 given 0.5; and those that incorporated 0-2 given 0.  ^5^ Specifically, the guideline recommends the comparator represent the usual practice in the decision-making setting. Models should therefore justify how their choice of comparator reasonably represents usual practice.  ^6^ The original checklist adds: “The number of radiographically confirmed peripheral fracture events per person-year is included in the dataset recommended by ProFaNE, classified using the ICD-10 classification system [2]. Fractures of the hip, wrist, and spine are the most common consequences of a fall. These should be reported individually. Other injuries as a result of a fall (e.g., traumatic brain injury) should be considered.” Models should incorporate granulated injury types. | | |

Ten items from the original Davis checklist were deemed primarily relevant to single-vehicle economic evaluations alongside randomized controlled trials rather than decision models and hence were excluded. Items 3.3 and 5.1 concerned methods for primary collection of falls and cost data in trial settings, respectively, while item 7.5 concerned methods for costing primary resource use data. Items 5.4, 8.3 and 8.4 concerned methods for handling missing data in trial settings, including the use of multiple imputation and identification of any biases resulting from non-random missing patterns. Item 3.8 concerned sample size calculation to detect statistically significant intervention effect, while Item 9.2 concerned estimation of the intervention effect and its 95% confidence interval. These issues regarding the quality of primary data collection and the calculation of effect in trial setting only indirectly affect decision models which can incorporate evidence from other (better designed and powered) trials and meta-analyses. Item 8.5 recommends that incremental cost per QALY ratios in cost-utility analyses are adjusted for baseline differences in HRQoL between intervention groups. Though this is feasible in decision models, the primary aim of the adjustment appears to be to control for covariates in identifying statistically significant differences in cost and health outcomes between the intervention groups in a trial. Indeed, Item 9.3 more explicitly recommends that statistical tests of significance be conducted on all cost and health outcomes. Such frequentist statistical tests that aim to reject or fail to reject a specific hypothesis are not relevant to decision models that are based on a Bayesian approach to statistics [3]. Items 8.5 and 9.3 were hence also excluded.

For Item 5, the original checklist recommends that the study describe the sample receiving the intervention, the method of recruitment and inclusion and exclusion criteria [1]. This recommendation appears to be primarily aimed at trial-based evaluations. Hence, the item modifies the original recommendation to better suit the modelling context. Specifically, the models should ensure that (or at least discuss whether) the inclusion and exclusion criteria, the methods of recruitment and the sample characteristics of external intervention studies that provide parameter estimates sufficiently match the models’ target population and intervention eligibility and access criteria. Out of the 8 components considered in this item, models that incorporated 6 or more are given a score of 1; those that incorporated 3-5 given 0.5; and those that incorporated 0-2 given 0.

# Excluded studies at full text screening stage

| **Table A3.** Studies excluded from systematic review at full text screening and exclusion reason. | | |
| --- | --- | --- |
| **First author (year)** | **Title** | **Main exclusion reason** |
| Benzinger (2016) | The impact of preventive measures on the burden of femoral fractures – a modelling approach to estimating the impact of fall prevention exercises and oral bisphosphonate treatment for the years 2014 and 2025 | Not full economic evaluation |
| Bray Jenkyn (2010) | Fall-related health service utilization, costs, and cost-effectiveness of a multi-factorial falls prevention program delivered to community-dwelling older adults | Not decision model |
| Busbee (2003) | Cost-utility analysis of cataract surgery in the second eye | No falls outcome |
| Campbell (2005) | Randomised controlled trial of prevention of falls in people aged > or =75 with severe visual impairment: the VIP trial | Not decision model |
| Cockayne (2017) | Clinical effectiveness and cost-effectiveness of a multifaceted podiatry intervention for falls prevention in older people: a multicentre cohort randomised controlled trial (the REducing Falls with ORthoses and a Multifaceted podiatry intervention trial) | Not decision model |
| Cohen (2015) | Prevention Program Lowered The Risk Of Falls And Decreased Claims For Long-Term Services Among Elder Participants | Not decision model |
| Church (2015) | Cost Effectiveness of Falls and Injury Prevention Strategies for Older Adults Living in Residential Aged Care Facilities | Not community-dwelling older population |
| Davis (2011) | Economic evaluation of dose-response resistance training in older women: a cost-effectiveness and cost-utility analysis | Not decision model |
| Davis (2011b) | Sustained economic benefits of resistance training among community-dwelling senior women | Not decision model |
| Davis (2020) | Action Seniors! Cost-effectiveness analysis of a secondary falls prevention strategy among community-dwelling older fallers | Not decision model |
| Department of Health (2009) | Impact assessment of fracture prevention interventions | Not community-dwelling older population |
| Evers (2020) | Economic evaluation of a home-based programme to reduce concerns about falls in frail, independently-living older people | Not decision model |
| Farag (2015) | Cost-effectiveness of a Home-Exercise Program Among Older People After Hospitalization | Not decision model |
| Farag (2016) | Economic evaluation of a falls prevention exercise program among people With Parkinson's disease | Not decision model |
| Fletcher (2012) | An exercise intervention to prevent falls in Parkinson's: an economic evaluation | Not decision model |
| Ghimire (2015) | Effects of a Community-Based Fall Management Program on Medicare Cost Savings | Not decision model; Not full economic evaluation |
| Harper (2019) | Cost analysis of a brief intervention for the prevention of falls after discharge from an emergency department | Not decision model |
| Haumschild (2003) | Clinical and economic outcomes of a fall-focused pharmaceutical intervention program | Not community-dwelling older population |
| Hendriks (2008) | Cost-effectiveness of a multidisciplinary fall prevention program in community-dwelling elderly people: a randomized controlled trial (ISRCTN 64716113) | Not decision model |
| Hiligsmann (2009) | Development and validation of a Markov microsimulation model for the economic evaluation of treatments in osteoporosis | Not falls prevention |
| Hiligsmann (2010) | Cost–effectiveness of osteoporosis screening followed by treatment: the impact of medication adherence | Not falls prevention |
| Irvine (2010) | Cost-effectiveness of a day hospital falls prevention programme for screened community-dwelling older people at high risk of falls | Not decision model |
| Isaranuwatchai (2017) | Cost-effectiveness analysis of a multifactorial fall prevention intervention in older home care clients at risk for falling | Not decision model |
| Johnson (2015) | Yield and cost-effectiveness of laboratory testing to identify metabolic contributors to falls and fractures in older persons | Not decision model |
| Kingkaew (2012) | Evidence to inform decision makers in Thailand: a cost-effectiveness analysis of screening and treatment strategies for postmenopausal osteoporosis | Not falls prevention |
| Lamb (2020) | Screening and Intervention to Prevent Falls and Fractures in Older People | Not decision model |
| Li (2015) | Economic Evaluation of a Tai Ji Quan Intervention to Reduce Falls in People With Parkinson Disease, Oregon, 2008-2011 | Not decision model |
| Li (2016) | Implementing an Evidence-Based Fall Prevention Intervention in Community Senior Centers | Not decision model |
| Li (2019) | Cost-Effectiveness of a Therapeutic Tai Ji Quan Fall Prevention Intervention for Older Adults at High Risk of Falling | Not decision model |
| Markle-Reid (2010) | The effects and costs of a multifactorial and interdisciplinary team approach to falls prevention for older home care clients 'at risk' for falling: a randomized controlled trial | Not decision model |
| Matchar (2018) | A Cost-Effectiveness Analysis of a Randomized Control Trial of a Tailored, Multifactorial Program to Prevent Falls Among the Community-Dwelling Elderly | Not decision model |
| Mueller (2008) | Cost effectiveness of the German screen-and-treat strategy for postmenopausal osteoporosis | Not falls prevention |
| Nayak (2011) | Cost-effectiveness of different screening strategies for osteoporosis in postmenopausal women | Not falls prevention |
| Nayak (2016) | Cost‐Effectiveness of Osteoporosis Screening Strategies for Men | Not falls prevention |
| Patil (2016) | Cost-effectiveness of vitamin D supplementation and exercise in preventing injurious falls among older home-dwelling women: findings from an RCT | Not decision model |
| Peeters (2011) | Multifactorial evaluation and treatment of persons with a high risk of recurrent falling was not cost-effective | Not decision model |
| Polinder (2016) | Cost-utility of medication withdrawal in older fallers: results from the improving medication prescribing to reduce risk of FALLs (IMPROveFALL) trial | Not decision model |
| Qin (2016) | Economic impact of using fesoterodine for the treatment of overactive bladder with urge urinary incontinence in a vulnerable elderly population in the United States | Not falls prevention |
| Sach (2012) | Community falls prevention for people who call an emergency ambulance after a fall: an economic evaluation alongside a randomised controlled trial | Not decision model |
| Schousbee (2013) | Cost-effectiveness of bone densitometry among Caucasian women and men without a prior fracture according to age and body weight | Not falls prevention |
| Si (2015) | Screening for and treatment of osteoporosis: construction and validation of a state-transition microsimulation cost-effectiveness model | Not falls prevention |
| Stanmore (2019) | The effectiveness and cost-effectiveness of strength and balance Exergames to reduce falls risk for people aged 55 years and older in UK assisted living facilities: a multi-centre, cluster randomised controlled trial | Not decision model |
| Stevens (2018) | The Potential to Reduce Falls and Avert Costs by Clinically Managing Fall Risk | Not decision model; Not full economic evaluation |
| Strom (2007) | Cost-effectiveness of alendronate in the treatment of postmenopausal women in 9 European countries-an economic evaluation based on the fracture intervention trial | Not falls prevention |
| Van Haastregt (2013) | Cost-effectiveness of an intervention to reduce fear of falling | No falls outcome |
| Zethraeus (2007) | Cost-effectiveness of the treatment and prevention of osteoporosis—a review of the literature and a reference model | Not falls prevention |

# Falls risk factors

| **Table A4.** Risk factors for fall-related events in models conducting primary analysis of epidemiological data or using published epidemiological evidence. | | | | | | | | | | | | | |
| --- | --- | --- | --- | --- | --- | --- | --- | --- | --- | --- | --- | --- | --- |
|  |  | **Falls risk factors** | | | | | | | | | | | |
| **Study label (n=33)^1^** | **Fall-related event** | Age | Sex | Ethnicity | Region/ Residence | Non-inj./MA falls history | Inj./MA falls history | Fear of falling | Chronic disease | Medication | Physical capacity | Service use history | Risk identification tool |
| ***Primary analysis of epidemiological data (n=8)*** | | | | | | | | | | | | | |
| BODE3 models | MA fall; Hospitalised fall; Fatal fall | ˟ | ˟ | ˟ |  |  | ˟ |  |  |  |  |  |  |
|  | Cataract surgery benefit in Boyd (2020) |  |  |  |  |  |  |  | ˟ |  |  |  |  |
| Eldridge (2005) | Any fall | ˟ |  |  |  | ˟ | ˟ |  | ˟ | ˟ | ˟ |  | FRAT |
|  | Fear of falling | ˟ |  |  | *LTC* | ˟ | ˟ |  | ˟ | ˟ | ˟ |  | FRAT |
|  | Fracture | ˟ |  |  | *LTC* |  |  | ˟ |  |  |  |  |  |
| Ippoliti (2018) | Hip fracture |  |  |  | *Region* |  |  |  |  |  |  |  |  |
| OMAS (2008) | MA fall; Fracture; Hip fracture; Fatal fall; LTC fall; Hospitalised fall | ˟ | ˟ |  |  |  | ˟ |  |  |  |  |  |  |
| Smith (2016) | MA fall | ˟ | ˟ |  |  |  | ˟ |  | ˟ | ˟ |  | ˟ | Internal tool |
| ***Use of published epidemiological evidence or expert opinion (n=25)*** | | | | | | | | | | | | | |
| Agartioglu (2020) | Any fall; MA fall; Hospitalised fall; Different injuries | *Single risk* | | | | | | | | | | | |
| Carande-Kulis (2015) | Any fall; Falls healthcare cost | ˟ |  |  |  |  |  |  |  |  |  |  |  |
| CSP (2016) | Any fall; Recurrent fall | ˟ | ˟ |  |  |  |  |  |  |  |  |  |  |
|  | Any fall |  |  |  |  |  |  |  |  |  |  |  | TUG |
|  | MA fall | *Single risk* | | | | | | | | | | | |
|  | No. of recurrent falls |  | ˟ |  |  |  |  |  |  |  |  |  |  |
|  | Care events | ˟ |  |  |  |  |  |  |  |  |  |  |  |
| Church (2011); (2012) | Any fall | ˟ |  |  |  | ˟ | ˟ |  |  |  |  |  |  |
|  | Fatal fall; Care events; LTC fall | ˟ |  |  |  |  |  |  |  |  |  |  |  |
| Farag (2015) | Any fall | ˟ |  |  |  | ˟ | ˟ |  |  |  |  |  |  |
|  | MA fall; Care events; LTC fall | ˟ |  |  |  |  |  |  |  |  |  |  |  |
| Franklin (2019) | Any fall; MA fall; Fatal fall; LTC fall; Care events | ˟ |  |  |  |  |  |  |  |  |  |  |  |
|  | Any fall |  |  |  |  |  |  |  |  |  |  |  | TUG & QTUG |
| Frick (2010) | Any fall; Hip fracture | *Single risk* | | | | | | | | | | | |
| Hiligsmann (2014) | Hip fracture; Vertebral fracture; Wrist fracture; Other fracture | ˟ | ˟ |  |  |  |  |  | *Exo* |  |  |  |  |
| Hirst (2016) | Hip fracture; Humerus fracture; Wrist fracture; Other fracture | ˟ | *Exo* |  |  |  |  |  |  | ˟ |  |  |  |
| Honkanen (2006) | Hip fracture; LTC hip fracture | ˟ | ˟ |  |  |  | ˟ |  |  |  | Funct. dep. |  |  |
|  | Functional dependency | ˟ | ˟ |  |  |  | ˟ |  |  |  |  |  |  |
| Howland (2015) | MA fall; Care events |  |  |  |  |  | *Exo* |  |  |  |  |  |  |
| Lee (2013) | Any fall; MA fall | ˟ | ˟ | *Exo* |  |  | *Exo* |  |  |  | Vit. D def. |  | Vit. D level |
| Ling (2008) | Any fall | ˟ |  |  |  | ˟ | ˟ |  | ˟ | ˟ | ˟ |  |  |
| Miller (2011) | Hospitalised fall | ˟ |  |  |  |  |  |  |  |  |  |  |  |
| Mori (2017) | Hip fracture; Humerus fracture; Wrist fracture; Other fracture | ˟ | *Exo* | *Exo* |  |  | ˟ |  | ˟ |  |  |  |  |
| Moriarty (2019) | Hip fracture; Other MA falls | ˟ |  |  |  |  | ˟ |  |  | ˟ |  |  |  |
| Nshimyu-mukiza (2013) | Hip fracture; Vertebral fracture; Wrist fracture | ˟ | *Exo* |  |  |  | ˟ |  | ˟ |  | BMD |  | Fracture risk tool |
|  | Fatal hip/vertebral fracture | ˟ |  |  |  |  |  |  |  |  |  |  |  |
| Poole (2014) | Hip fracture; Fatal hip fracture | ˟ | ˟ |  |  |  |  |  |  |  |  |  |  |
| Poole (2015) | MA fall; Hospitalised fall; LTC fall; Fatal fall | ˟ |  |  |  |  |  |  |  |  |  |  |  |
| RCN (2005) | MA fall; Severity of MA fall | ˟ |  |  |  |  |  |  |  |  |  |  |  |
| Tannenbaum (2015) | Any fall |  |  |  |  |  | ˟ |  | *Exo* | ˟ |  |  |  |
| Turner (2020) | Non-fracture MA fall; Hospitalised hip & non-hip fractures; Fatal hip fracture |  |  |  |  |  |  |  | *Exo* | ˟ |  |  |  |
| Wu (2010) | Recurrent MA fall | ˟ |  |  |  |  | *Exo* |  |  |  |  |  |  |
| Zarca (2014) | Hip fracture | ˟ | ˟ |  |  |  | ˟ |  |  |  | Vit. D level |  | Vit. D level |
|  | Post-hip fracture excess mortality |  | ˟ |  |  |  |  |  |  |  |  |  |  |
| **Abbreviation:** BMD: bone mass density; BODE3: Burden of Disease Epidemiology, Equity and Cost-Effectiveness Programme studies, including Boyd (2020), Deverall (2018), Pega (2016) and Wilson (2017); CSP: Chartered Society of Physiotherapy; Funct. dep.: functional dependency; FRAT: fall risk assessment tool; LTC fall: fall requiring long-term care; MA fall: fall requiring medical attention; OMAS: Ontario Medical Advisory Secretariat; QTUG: quantitative timed up and go test; RCN: Royal College of Nursing; TUG: timed up and go test; Vit. D def.: vitamin D deficiency  **Note:** ‘*Exo*’ denotes the case where a risk factor is exogenous to the model evaluation. ‘*Single Risk*’ denotes the case where the model incorporates a single event risk/rate.  ^1^ See Table 2 in main manuscript text for study references; parenthesised number refers to the number of models included in the table. | | | | | | | | | | | | | |

# Health utilities data

| **Table A5.** Summary of health utilities data and source used in decision models | | | |
| --- | --- | --- | --- |
| **Study label (n=29)^1^** | **Health state** | **Health utility** | **Source** |
| Albert (2016) | Health states defined by all-cause care utilisation frequencies, falls incidence and intervention receipt | EQ-5D range 0.263-0.942 | Internal data |
| Alhambra-Borras (2019) | Improved falls risk and/or frailty from baseline | EQ-5D 0.81 | Internal data |
|  | Same/worse falls risk and/or frailty | EQ-5D 0.75 | Internal data |
| BODE3 models | New Zealand population norm aged 65+ | Vary by age, sex and ethnicity; not reported. |  |
|  | MA fall | DW 0.100 for 1 year  (95% CI 0.060-0.150) | [4] |
|  | Vision improvement from cataract removal in Boyd (2020) | QALY gain of 0.057 (95% CI 0.041-0.075) | [4] |
| Church (2011); (2012) | Australian population aged 65+ | EQ-5D range 0.676-0.806; vary by age | [5] |
|  | Fall requiring ED visit | EQ-5D loss 0.014 (Range 0.010-0.016) for 1 year | [6] |
|  | Hospitalised fall | EQ-5D loss 0.144 (Range 0.000-0.255) for 1 year | [5, 6] |
|  | LTC admission (fall/other cause) | EQ-5D loss 0.060 (Range 0.030-0.338) for 1 year | [7] |
|  | Post-fracture state | EQ-5D loss 0.072 (Range 0.000-0.128) for 1 year | [5, 6] |
|  | Fear of falling after any fall | EQ-5D loss 0.045 (Range 0.033-0.058) for 1 year | [8] |
| Eldridge (2005) | No fall or fear | TTO value of 1.000 | Assumed |
|  | Fear of falling | TTO value of 0.670 | [9] |
|  | Hip fracture, no LTC admission | TTO value of 0.310 | [9] |
|  | Hip fracture, LTC admission | TTO value of 0.050 | [9] |
| Farag (2015) | Australian population aged 65 | EQ-5D 0.806 (Range 0.676-1); vary by age group | [5] |
|  | Fall not requiring ED visit | EQ-5D loss 0.018 (Range 0.013-0.070) for 1 year | [5] |
|  | Fall requiring ED visit | EQ-5D loss 0.040 (Range 0.014-0.150) for 1 year | [10] |
|  | Hospitalised fall, 1^st^ year | EQ-5D loss 0.239 (Range 0.144-0.250) for 1 year | [10] |
|  | Hospitalised fall, 2^nd^ year | EQ-5D loss 0.126 (Range 0.050-0.200) for 1 year (unclear whether permanent) | [10] |
|  | LTC admission (fall/other cause) | EQ-5D loss 0.100 (Range 0.030-0.940) for 1 year | [5, 11, 12] |
| Franklin (2019) | UK population norm aged 65+ | EQ-5D 0.780 (SE 0.11) for age 65; vary by age | [13, 14] |
|  | Fall requiring A&E, not hospital | EQ-5D loss 0.025 (SE 0.003) for 1 year | [15] |
|  | Hospitalised fall | EQ-5D loss 0.098 (SE 0.010) for 1 year | [15] |
|  | Fall requiring LTC admission | EQ-5D loss 0.194 (SE 0.019) for 1 year | [16] |
| Frick (2010) | US population aged 65+ | EQ-5D 0.823 (SD 0.025) | [17] |
|  | Hip fracture, 1^st^ year | EQ-5D loss 0.200 (SD 0.010) for 1 year | [17] |
|  | Hip fracture, after 1^st^ year | EQ-5D loss 0.060 (SD 0.010) each year | [17] |
| Hiligsmann (2014) | Hip fracture, 1^st^ year | Multiplier 0.80 (Range 0.770-0.825) for 1 year | [18] |
|  | Hip fracture, after 1^st^ years | Multiplier 0.90 (Range 0.885-0.910) each year | [18] |
|  | Vertebral fracture, 1^st^ year | Multiplier 0.72 (Range 0.660-0.775) for 1 year | [18] |
|  | Vertebral fracture, after 1^st^ year | Multiplier 0.93 (Range 0.916-0.946) each year | [18] |
|  | Wrist fracture, 1^st^ year | Multiplier 0.94 (Range 0.910-0.960) for 1 year | [18] |
|  | Wrist fracture, after 1^st^ year | Multiplier 1.00 each year | [18] |
|  | Other fracture, 1^st^ year | Multiplier 0.91 for 1 year | [18] |
|  | Other fracture, after 1^st^ year | Multiplier 1.00 each year | [18] |
| Hirst (2016) | UK women aged 75+ | EQ-5D 0.710 (SE 0.02) | [19] |
|  | Hip fracture | Multiplier 0.7 (SE 0.14) for 1 year | [20] |
|  | Humerus, wrist and other fracture | Multiplier 0.934 (SE 0.19) for 1 year | [20] |
| Honkanen (2006) | US population aged 60+ | TTO range 0.792-0.841; vary by age group, sex | [21] |
|  | Hip fracture | TTO loss 0.312 (Range 0.000-0.692) for 1 year | [5] |
|  | Post-hip fracture, non-disabling | HUI2 loss 0.112 (Range 0.000-0.482) each year | [22] |
|  | Functional dependence | HUI2 loss 0.170 (Range 0.140-0.230) each year | [23] |
|  | LTC admission | HUI2 loss 0.060 (Range 0.030-0.338) each year | [23] |
|  | Hip protector use | Utility loss 0.010 (Range -0.005-0.050) each year | [24] |
| Johansson (2008) | Swedish population aged 65+ | EQ-5D range 0.660-0.780; vary by age group, sex | [25] |
|  | Hip fracture | EQ-5D loss 0.170 for 1 year (unclear whether permanent) | [26] |
| Lee (2013) | US population aged 65+ | EQ-5D range 0.724-0.840; vary by age group, sex | [27] |
|  | Non-MA fall, no fear of falling | EQ-5D loss 0.044 (Range 0.000-0.075) for 1 year | [8, 10] |
|  | MA fall, fear of falling | EQ-5D loss 0.161 (Range 0.105-0.253) for 1 year | [8, 10] |
| McLean (2015) | No fall | EQ-5D 1.000 for 18 months | Assumed |
|  | Any fall [women only] | EQ-5D 0.993 [0.985] for 18 months | [8, 28, 29] |
|  | Hip fracture | EQ-5D 0.730 for 18 months | [10] |
|  | Shoulder fracture | EQ-5D 0.940 for 18 months | [30] |
|  | Wrist fracture [women only] | EQ-5D 0.969 [0.966] for 18 months | [10] |
|  | Other fracture [women only] | EQ-5D 0.958 [0.955] for 18 months | [30] |
| Mori (2017) | US population aged 65+ | EQ-5D range 0.677-0.801; vary by age group | [27] |
|  | Hip fracture, 1^st^ year | Multiplier 0.776 (Range 0.720-0.844) for 1 year | [31, 32] |
|  | Hip fracture, after 1^st^ year | Multiplier 0.855 (Range 0.800-0.909) each year | [31, 32] |
|  | Vertebral fracture, 1^st^ year | Multiplier 0.724 (Range 0.667-0.779) for 1 year | [31, 32] |
|  | Vertebral fracture, after 1^st^ year | Multiplier 0.868 (Range 0.827-0.922) each year | [31, 32] |
|  | Wrist fracture, 1^st^ year | Multiplier 0.940 (Range 0.910-0.960) for 1 year | [18] |
|  | Other fracture, 1^st^ year | Multiplier 0.910 (Range 0.880-0.940) for 1 year | [18] |
| Moriarty (2019) | UK population aged 65+ | EQ-5D VAS 0.770 (Beta 129, 39) for age 65-74; 0.740 (Beta 109, 38) for age 75+ | [13] |
|  | Hip fracture | Loss of 0.203 (Gamma 209, 1031) | [18] |
|  | Other MA falls | EQ-5D loss of 0.060 (Gamma 22, 369) | [26] |
|  | LTC admission (hip fracture/other cause) | HUI2 loss of 0.060 (Gamma 1, 10) (unclear whether permanent) | [7, 33] |
| Nshimyumukiza (2013) | Hip fracture hospitalisation | HUI3 0.300 (Range 0.510-0.600) for 1 year | [34] |
|  | Hip fracture rehabilitation | HUI3 0.560 (Range 0.630-0.700) for 1 year | [34] |
|  | Hip fracture post-rehabilitation | HUI3 0.850 (Range 0.730-0.900) each year | [34] |
|  | Vertebral fracture hospitalisation | HUI3 0.330 for 1 year | [35, 36] |
|  | Vertebral fracture rehabilitation | HUI3 0.680 for 1 year | [35, 36] |
|  | Vertebral fracture post-rehabilitation | HUI3 0.850 (Range 0.760-0.900) each year | [35, 36] |
|  | Wrist fracture hospitalisation | HUI3 0.610 for 1 year | [36] |
|  | Wrist fracture rehabilitation | HUI3 0.880 for 1 year | [36] |
|  | Wrist fracture post-rehabilitation | HUI3 1.000 (Range 0.820-1.000) each year | [36] |
| Poole (2015) | UK population aged 60+ | EQ-5D range 0.730-0.800; vary by age group | [13] |
|  | Fall requiring A&E, not hospital | EQ-5D range 0.713-0.783 by age group | [15] |
|  | Hospitalised fall | EQ-5D range 0.698-0.768 by age group | [15] |
|  | Fall requiring LTC admission | EQ-5D range 0.536-0.606 by age group | [37] |
| PHE (2018) | UK population aged 75+ | EQ-5D 0.730 | [13, 16] |
|  | Fall requiring GP or A&E | EQ-5D 0.730 | [13, 16] |
|  | Hip fracture, hospitalisation | EQ-5D 0.582 each year | [5] |
|  | Non-hip fracture or non-fracture, hospitalisation | EQ-5D 0.699 each year | [5] |
|  | Fall requiring LTC admission | EQ-5D loss 0.060 each year | [38] |
|  | Fear of falling after MA fall | EQ-5D loss 0.045 each year | [8, 38] |
| RCN (2005) | UK population aged 60+ | EQ-5D 0.800 for age 60-64 and 65-69; 0.750 for 70-74 and 75+ | [19] |
|  | Hip fracture | Multiplier unspecified; range 0.166 for 60-69 and 0.146 for 80+ | Assumed |
|  | Non-hip fracture | Multiplier unspecified; range 0.074 for 60-69 and 0.065 for 80+ | Assumed |
| Sach (2007); (2010) | Patient-level variation for women aged 70+ with bilateral cataracts | EQ-5D (range not reported) | Internal data |
| Tannanbaum (2015) | Insomnia patients, no treatment | SF-6D 0.630 | [39] |
|  | Insomnia patients, treatment | SF-6D 0.660 | [39] |
|  | Any fall | EQ-5D loss 0.030 for 6 months | [8] |
|  | Fear of falling after fall | EQ-5D loss 0.060 each 6-month cycle | [8] |
|  | Hip fracture | EQ-5D loss 0.170 for 6 months | [40] |
|  | Vertebral and write fractures | EQ-5D loss 0.140 for 6 months | [40] |
| Turner (2020) | Insomnia patients, sedative users | SF-6D 0.630 | [39] |
|  | Non-fracture fall | Loss of 0.005 (Gamma) | [41] |
|  | Non-hip fracture | EQ-5D loss of 0.025 (Gamma) | [26] |
|  | Hip fracture | EQ-5D loss of 0.019 (Gamma) | [26] |
| Zarca (2014) | French population aged 60+ | EQ-5D 0.780 for 60-69; 0.740 for 70-79 and 80-89 | [25] |
|  | Hip fracture, 1^st^ year | Multiplier 0.79 to norm for 1 year | [42, 43] |
|  | Hip fracture, 2^nd^ year | Multiplier 0.81 for 1 year | [42, 43] |
|  | Hip fracture, after 2^nd^ year | Multiplier 0.90 each year | [42, 43] |
| **Abbreviation:** BODE3: Burden of Disease Epidemiology, Equity and Cost-Effectiveness Programme studies, including Boyd (2020), Deverall (2018), Pega (2016) and Wilson (2017); CI: confidence interval; DW: disability weight; ED: emergency department; LTC: long-term care; MA fall: fall requiring medical attention; PHE: Public Health England; QALY: quality-adjusted life year; RCN: Royal College of Nursing; SD: standard deviation; SE: standard error; TTO: time trade-off; VAS: visual analogue scale  ^1^ See Table 2 in main manuscript text for study references; parenthesised number refers to the number of models included in the table. | | | |

# Falls economic consequences

| **Table A6.** Summary of economic consequences of falls from the health and social care perspective included in decision models.^1^ | | | | | | | |
| --- | --- | --- | --- | --- | --- | --- | --- |
| **Study label (n=46)^2^** | Ambulatory care | ED | Hospital-isation | Rehabili-tation | Short-term social care | LTC admission | Comorbidity care cost |
| Agartioglu (2020) | ˟ | ˟ | ˟ |  |  |  |  |
| Albert (2016) |  | AC | AC |  |  |  |  |
| Alhambra-Borras (2019) | AC |  | AC |  |  |  |  |
| Beard (2006) | ˟ | NS | ˟ | ˟ | NS | ˟ |  |
| BODE3 models | ˟ |  | ˟ | ˟ |  |  | ˟ |
| Carande-Kulis (2015) | NS | NS | NS | NS |  |  |  |
| CSP (2016) | ˟ | ˟ | ˟ | ˟ |  | ˟ |  |
| Church (2011); (2012) | NS | ˟ | ˟ | ˟ |  | ˟ |  |
| Comans (2009) | ˟ | ˟ | ˟ | ˟ | ˟ | ˟ |  |
| Day (2009); (2010) |  |  | No cost^3^ |  |  |  |  |
| Eldridge (2005) | NS | NS | NS | ˟ |  | ˟ |  |
| Farag (2015) | NS | ˟ | ˟ | NS |  | ˟ |  |
| Franklin (2019) | ˟ | ˟ | ˟ | ˟ |  | ˟ |  |
| Frick (2010) | NS | NS | NS | NS | NS | NS |  |
| Hektoen (2009) | ˟ | ˟ | ˟ | ˟ | ˟ | ˟ |  |
| Hiligsmann (2014) | NS | NS | NS | NS |  | ˟ |  |
| Hirst (2016) | NS | NS | NS | NS |  | ˟ |  |
| Honkanen (2006) | ˟ | NS | ˟ | ˟ |  | ˟ | ˟ |
| Howland (2015) |  | ˟ | ˟ |  |  |  |  |
| Ippoliti (2018) |  |  | ˟ | ˟ |  |  |  |
| Johansson (2008) | ˟ | NS | ˟ | ˟ | ˟ | ˟ | ˟ |
| Lee (2013) | ˟ | NS | ˟ |  |  |  |  |
| Ling (2008) | NS | NS | NS | NS |  | ˟ |  |
| McLean (2015) | ˟ | ˟ | ˟ | ˟ |  |  |  |
| Miller (2011) | ˟ | ˟ | ˟ | ˟ |  |  |  |
| Mori (2017) | NS | NS | NS | NS |  | ˟ |  |
| Moriarty (2019) | ˟ | ˟ | ˟ | ˟ |  | ˟ |  |
| Nshimyumukiza (2013) |  | ˟ | ˟ | ˟ |  | ˟ |  |
| OMAS (2008) |  | ˟ | ˟ | ˟ |  | ˟ |  |
| Poole (2014); (2015) | ˟ | ˟ | ˟ | ˟ |  | ˟ |  |
| PHE (2018) | ˟ | ˟ | ˟ | ˟ |  | ˟ |  |
| RCN (2005) | ˟ | ˟ | ˟ | ˟ |  | ˟ |  |
| Sach (2007); (2010) | AC | AC | AC | AC | AC | AC |  |
| Smith (2016) | ˟ |  | ˟ |  |  |  |  |
| Tannenbaum (2015) |  | ˟ | ˟ |  |  |  |  |
| Turner (2020) | ˟ | ˟ | ˟ | ˟ |  |  |  |
| Velde (2008) |  | ˟ | ˟ | ˟ | ˟ | ˟ |  |
| Wu (2010) |  | ˟ | ˟ | ˟ |  |  |  |
| Zarca (2014) |  |  | ˟ |  |  |  |  |
| **Abbreviation:** AC: all-cause; BODE3: Burden of Disease Epidemiology, Equity and Cost-Effectiveness Programme studies, including Boyd (2020), Deverall (2018), Pega (2016) and Wilson (2017); CSP: Chartered Society of Physiotherapy; ED: emergency department; Int. cost only: intervention cost only; LTC: long-term care; NS: not (precisely) specified; OMAS: Ontario Medical Advisory Secretariat; PHE: Public Health England; RCN: Royal College of Nursing.  ^1^ Economic consequences are marked even if only their costs are incorporated without separate model states or probabilities.  ^2^ See Table 2 in main manuscript text for study references; parenthesised number refers to the number of models included in the table.  ^3^ The models estimated the intervention impact on hospital admission rate but not cost and used only intervention costs in conducting cost-effectiveness analysis. | | | | | | | |

# Intervention characteristics summary

| **Table A7.** Summary of interventions evaluated in models | | |
| --- | --- | --- |
| **Study label (n=46)^1^** | **Intervention type** | **Description** |
| Agartioglu (2020) | Community-based HAM | - Comparator: usual care - Component: older people in community visited at home monthly for 3 months; each visit lasted 40 minutes; home security checklist filled out. - Pathway: unclear - Resource/cost: implemented by GP nurse with 1-day training; salary; booklet. - Efficacy source: internal RCT; meta-analysis [44]; previous model which used meta-analysis [33] |
| Albert (2016) | Multifactorial intervention – Healthy Steps for Older Adults | - Comparator: community centre attendance only without intervention receipt - Component: FRA (TUG, one-legged stand, 60-second chair stand); referral to physician and HAM for participants scoring below age- and gender-based norms; education on home hazards and exercise. - Pathway: self-referred – voluntary take-up at community senior centres - Resource/cost: FRA by professional or trained volunteer; state department assured fidelity by training staff, monitoring data and interviewing participants; per-participant reimbursement from state department. - Efficacy source: internal quasi-experimental study |
| Alhambra-Borras (2019) | Exercise – nine-month group-based multi-component physical exercise | - Comparator: usual care - Component: 60 balance and strength exercise routines – 34 from Otago (adapted to group setting) and 26 designed *ad hoc* by PT leader; two 45-minute sessions per week; session supervised by PT; excluded walking component from Otago; aim to affect frailty as well as falls. - Pathway: proactive – assessed by home visit at baseline for study inclusion eligibility (i.e., at high falls risk and/or frail) - Resource/cost: PT labour; training and community venue but not costed - Efficacy source: internal quasi-experimental study |
| Beard (2006) | Intersectoral intervention, Stay on Your Feet, including multifactorial and environmental interventions | - Comparator: usual care in control regions - Component: education, HAM, exercise and public space safety improvements; FRA part of multifactorial intervention; targeting 8 falls risk factors (balance, gait, insufficient exercise, inappropriate footwear, poor vision, medication use, undertaking medical conditions, environmental hazards) - Pathway: self-referred – investment in marketing; environmental - Resource/cost: local clinicians and community staff labour; printing; marketing; overheads (administration); exercise cost borne by participants included in societal evaluation; travel and HAM costs borne by participants excluded from evaluation; time opportunity cost of local clinicians and community staff excluded; lobbying cost for public safety improvements included. - Efficacy source: internal quasi-experimental study |
| Boyd (2020) | Cataract surgery: (i) Expedited; (ii) Non-expedited first-eye surgery | - Comparator: no surgery receipt - Component: expedited surgery involves public sector purchase of private sector practice and reduces waiting time by 12 months; non-expedited surgery involves public sector delivery; surgery generates permanent vision improvement - Pathway: unclear – likely proactive; but no mention of how cataracts diagnosed - Resource/cost: specialist service; public sector reimbursement for private or public sector delivery - Efficacy source: external RCT [45] |
| Carande-Kulis (2015) | (i) Otago exercise; (ii) Tai Chi – Moving for Better Balance; (iii) ‘Stepping On’ – multiple-component intervention | - Comparator: unclear – control group in external RCTs - Component: (i) Otago – individually tailored muscle-strengthening and balance-retraining exercises of increasing difficulty combined with a walking programme; set of in-home exercises for appropriate and increasing levels of difficulty and walking plan; home visits by PT or trained nurse in first two months; (ii) Tai Chi – one-hour sessions of Tai Chi including warm-up and cool-down; 24 Tai Chi forms for weight shifting, postural alignment, and coordinated movements; three classes per week for 26 weeks; delivered by Tai Chi instructors; implemented in senior centres, adult activity centres and community centres; (iii) Stepping On – seven weekly three-hour group sessions in community setting with follow-up home visits; led by OT who introduced exercises and sessions on topics related to falls; most sessions attended by trained volunteer discussing medication management, home and community safety, sleep quality, and hip protector use. - Pathway: self-referred – cost of 10 hours of pre-intervention marketing; but also includes Otago strategy targeting persons aged 80+ – unclear how - Resource/cost: marketing; staff labour; training – lead trainer to train instructors who need retraining; materials; volunteer labour under Stepping On not costed - Efficacy source: external RCTs [46-48] |
| CSP (2016) | Falls risk screening and physiotherapy | - Comparator: no physiotherapy - Component: different physiotherapy forms – physiotherapy only, individual exercise, group exercise, modern exercise, all forms combined - Pathway: proactive – referral after TUG risk screening - Resource/cost: TUG screening not costed; two clients per PT; 20% of PTs implement group exercise, six per group; each PT provides 4.6 sessions per year. - Efficacy source: external meta-analysis [44] |
| Church (2011) | Multiple types | - Types: (A) For general older populations: (i) group exercise; (ii) home exercise; (iii) Tai Chi; (iv) Stepping On – multiple-component intervention; (v) multifactorial intervention; (vi) multifactorial risk assessment; (B) For specific older populations: (i) expedited cataract surgery; (ii) psychotropic medication withdrawal; (iii) cardiac pacing. - Comparator: no intervention received - Component: (A) General: (i) group exercise – two group classes and one home exercise session per week for 26 weeks; (ii) home exercise – five district nurse home visits in the first week, followed by home visits at week 2, 4 and 8 weeks with a booster at 6 months; (iii) Tai Chi – 6-month instructed classes twice a week for 12 participants; (iv) Stepping On – two-hour weekly group information sessions on falls prevention run by OT for 7 weeks, follow-up home visit, 2-hour nurse interview; (v) multifactorial intervention – FRA plus weekly exercise, HAM by OT, vision assessment, medication review and counselling; (vi) multifactorial risk assessment – FRA plus physician follow-up, 1-hour OT home visit and 2-hour nurse interview; (B) Specific: (i) expedited cataract surgery – surgery within 4 weeks vs. usual 12-month and two specialist visits; (ii) medication reduction over 14 weeks with six GP visits and nurse time; (iii) cardiac pacing – screening by carotid sinus massage, cardiovascular assessment, insertion of a pacemaker and post-pacemaker visit. - Pathway: unclear – screening required to identify specific patient groups but not mentioned or costed. - Resource/cost: see components. - Efficacy source: external meta-analysis [44] |
| Church (2012) | Multiple types | - Types: (A) For general-risk older populations: (i) group exercise; (ii) home exercise; (iii) Tai Chi; (iv) multiple-component intervention; (v) multifactorial intervention; (vi) multifactorial risk assessment; (B) For high-risk older populations: (i) group exercise; (ii) HAM; (iii) multifactorial intervention; (C) For specific older populations: (i) expedited cataract surgery; (ii) psychotropic medication withdrawal; (iii) cardiac pacing. - Comparator: no intervention received; cross-comparison between alternatives - Component: general- and high-risk group exercise – two classes per week for 26 weeks and home exercise; Tai Chi – two classes per week for six months with 12 participants per group; cataract surgery – not stated; multiple-component intervention – see Carande-Kulis (2015); other interventions – see Day (2009) - Pathway: unclear – no mention of how high-risk populations were identified - Resource/cost: 30% administration fee for group exercise (general- and high-risk) and Tai Chi but other resource/cost not stated; diagnostic-related group reimbursement rate for cataract surgery; multiple-component intervention – see Carande-Kulis (2015); other interventions – see Day (2009) - Efficacy source: external meta-analysis [44] |
| Comans (2009) | Multifactorial intervention: (i) centred-based; (ii) home-based | - Comparator: no intervention receipt - Component: for both multifactorial forms – FRA; Tai Chi; HAM; education; dietary advice - Pathway: unclear – likely proactive since targets persons with recent falls history, self- or GP-identified functional decline or self-reported gait instability; but no mention of routine care screening - Resource/cost: fixed – office space, equipment, storage, motor vehicle lease; variable – motor vehicle running, PT/OT labour, consumables - Efficacy source: external RCT [48] |
| Day (2009) | Multiple types | - Types: (i) Tai Chi for mobile 70+; (ii) home exercise for mobile 80+; (iii) HAM for all-cause hospital inpatients 65+ (including cognitively impaired) with falls history; (iv) multifactorial intervention for fall patients 65+ admitted to ED; (v) psychotropic medication withdrawal for medication users 65+; (vi) cardiac pacing for fall patients 50+ admitted to ED and have cardioinhibitory carotid sinus hypersensitivity. - Comparator: no intervention receipt - Component: (i) Tai Chi – group class twice per week (45 minutes, 12 participants per group) for 15 weeks and twice-daily home practice; progressive difficulty (gradual reduction of standing support until single limb stance achieved, increased body and rotation, increased reciprocal arm movements); (ii) home exercise – muscle-strengthening and balance retraining individually prescribed by PT-trained district or GP nurse and twice-weekly walks; five home visits over first six months and monthly telephone call to boost motivation over one year; (iii) HAM – home assessment around one hour conducted by OT using standardised home assessment form; list of specific recommendations on modifications made; telephone follow-up two weeks later to check modifications and encourage compliance; (iv) multifactorial intervention – falls risk screening at ED admission for fall; 36% referred to hospital outpatient department; 21% referred to multidisciplinary falls clinic – FRA by geriatrician, PT and nurse followed by tailored treatments (home/group exercise, gait aid change, footwear change, footcare, hip protectors, day hospital service, further medical tests); 18% referred to GP; 15% referred to optometrist; 100% received HAM by OT; (v) psychotropic medication withdrawal – doses of benzodiazepine, other hypnotic, antidepressant or major tranquiliser gradually reduced over 14 weeks: 80% of original dose after two weeks, 60% after five, 40% after eight, 20% after 11, placebo by 14; (vi) cardiac pacing – carotid screening, specialist consultation and cardiovascular assessment, pacemaker insertion, post-insertion specialist visit - Pathway: (i) Tai Chi – self-referred with marketing; (ii) home exercise – proactive because GPs’ time included in recruitment; (iii) HAM – reactive, though for all-cause hospital inpatients; (iv) multifactorial intervention – reactive; (v) psychotropic medication withdrawal – proactive, users referred by GPs; (vi) cardiac pacing – reactive. - Resource/cost: (i) Tai Chi – instructor labour, venue, music license, community marketing, administration; (ii) home exercise – staff training, recruitment, material, staff labour; (iii) HAM – staff labour and travel, equipment; (iv) multifactorial intervention – falls risk identification, staff labour, equipment, overhead; (v) psychotropic medication withdrawal – falls risk screening, medication cost, staff labour; (vi) cardiac pacing – staff labour, equipment. - Efficacy source: external RCTs [46, 49-54] |
| Day (2010) | Group-based Tai Chi | - Comparator: no intervention receipt - Component: group-based Tai Chi for persons 70+ without profound limits in communication, mobility and self-care; twice weekly classes for 26 weeks - Pathway: self-referred – participants recruited through newspaper advertisement - Resource/cost: recruitment; coordination; staff labour; venue; music license fee - Efficacy source: external meta-analysis (2009 version) [44] |
| Deverall (2018) | Exercise: (i) Peer-led group exercise; (ii) Home exercise; (iii) Commercial group exercise | - Comparator: no intervention receipt - Component: (i) peer-led group exercise “Steady As You Go” – based on Otago exercise programme (OEP); weekly sessions, first 10 weeks supervised by trained staff, after which a group member trained as peer leader; individuals can participate until age 90 or death; (ii) home exercise – physiotherapy-based exercise individually tailored by nurse specialist; based on OEP; home visits at week 1, 2, 4 and 8 and at 6 months; (iii) commercial group exercise – group exercise classes in commercial gym, not specifically designed for older adults; focus on balance and strength (e.g., Tai Chi, Pilates, Yoga) - Pathway: self-referred – discusses mass media campaigns to promote uptake - Resource/cost: (i) group exercise – private transport costs for 50% of group exercise participants; venue hire using donations; maintained for 25 years with same cost; (ii) home exercise – recruitment, equipment, consumables, staff labour, overheads; (iii) commercial exercise – cost of gym class enrolment - Efficacy source: external meta-analysis [44] |
| Eldridge (2005) | Falls risk screening and multifactorial intervention at falls clinic or bi-disciplinary gait and balance exercise | - Comparator: usual care in primary care trust (PCT) area - Component: facilitator at PCT to introduce programme; enhancement of existing falls risk screening and referral system; screening using Falls Risk Assessment Tool (FRAT) – high falls risk if three or more risk factors out of falls history, 4+ prescribed medication per day, stroke or Parkinson’s disease, balance problems, and inability to rise from chair without using arms; screening conducted in community by primary healthcare and social care staff and in hospital and A&E by healthcare staff; establishment of a falls clinic if none existed multidisciplinary FRA and treatment by geriatrician, nurse, OT and PT (multifactorial intervention); reactive patients always referred to falls clinic; proactive patients referred to falls clinic or OT/PT (bi-disciplinary) gait and balance treatment - Pathway: (i) proactive – screening by FRAT conducted by primary healthcare and social care services plus additional screening by GP before referral to falls clinic or OT/PT gait and balance treatment; (ii) reactive – screening by FRAT conducted by healthcare staff at A&E and hospital before referral to falls clinic; (iii) self-referred – individuals screened but not referred can still self-refer to (presumably) gait and balance exercise (50% of false and 10% of true negative individuals self-refer). - Resource/cost: fixed cost – programme set-up; staff labour (facilitator, OT/PT); falls clinic running; printing and miscellaneous; per-participant cost – staff labour cost (GP, primary and community nurses, A&E staff) administering FRAT. - Efficacy source: external meta-analysis (2001 version) [44]; same efficacy applied to falls clinic and OT/PT treatment. |
| Farag (2015) | Non-specific intervention | - Comparator: no programme condition - Component: non-specific intervention representative of “intervention strategies suited to application to the broader population” such as individual/group exercise and multifactorial intervention. - Pathway: self-referred – intervention targets individuals without falls history; mentions information provision to community groups and GPs to increase uptake. - Resource/cost: not stated; per-participant cost of AUS$700 - Efficacy source: assumption |
| Franklin (2019) | Falls risk screening and: (i) Otago home exercise; (ii) FaME group exercise; (iii) Group Tai Chi; (iv) HAM | - Comparator: no falls risk screening and no treatment; strategy cross comparison - Component: TUG or QTUG for falls risk screening – in QTUG individuals wear inertial sensor on each leg whilst performing TUG; both performed by GP nurse; (i) Otago – multiple-component exercise conducted at home; trained instructor teaches participants and monitors progress; initial assessment performed by PT or postural stability instructor to determine initial difficulty; 10 contact hours over year; (ii) FaME – multiple-component group exercise programme delivered by postural stability instructor; weekly group sessions for 45-75 minutes plus home exercises for six months; (iii) Tai Chi – performed at home or in group; exercises combining deep breathing and relaxation with flowing movements; (iv) HAM – professional assesses person’s usual residence to identify environmental hazards (e.g., poor lighting, no handrails) and carries out actions to reduce these - Pathway: proactive – only screened high-risk individuals referred - Resource/cost: TUG/QTUG – set-up cost (training for QTUG); staff labour; equipment; treatments – costs from PHE (2018) including 5% evaluation cost - Efficacy source: external meta-analysis for TUG efficacy [55]; external study for QTUG efficacy [56]; external meta-analyses for treatments [44, 57]. |
| Frick (2010) | Multiple types | - Types: (i) multifactorial intervention for general-risk population; (ii) multifactorial intervention for high-risk population (fallen in past year); (iii) HAM for high-risk population; (iv) vitamin D supplementation; (v) medication modification; (vi) exercise; (vii) Tai Chi. - Comparator: standard care – standard medical evaluation on health status and routine examinations and treatments where needed; cross comparisons - Component: (i/ii) multifactorial intervention – not stated; (iii) HAM – delivered by OT, PT, nurses; (iv) vitamin D – 800 IU per day; (v) medication modification – management of central nervous system drugs, particularly withdrawal of psychotropics such as benzodiazepines, anti-depressants and antipsychotics; (vi) exercise – muscle and balance training; (vii) Tai Chi – not stated - Pathway: unclear – screening required for (ii), (iii) and (v) but not mentioned and not costed, though possibly included in overheads - Resource/cost: staff labour (adjusted upwards by 30% to cover benefits); overheads (adjusted upwards by 50% for office and administrative costs) - Efficacy source: external meta-analysis (2003 version) [44] |
| Hektoen (2009) | Otago home exercise | - Comparator: RCT control group - Component: strength and balance re-training for people at high risk of falling; for first two months, four one-hour home visits by PT who gave instructions for home-based training; for next 10 months, PT made telephone calls every second month; exercise lasted 30 minutes per session and performed three times weekly; walking plan - Pathway: self-referred – RCT invested resources for participant recruitment but no mention of professional referrals. - Resource/cost: recruitment; staff labour (exercise instruction, follow-up telephone calls); equipment - Efficacy source: external RCT [58] |
| Hiligsmann (2014) | Vitamin D and calcium supplementation for osteoporotic patients | - Comparator: no supplementation - Component: one BMD measurement at first and third years of three-year supplementation programme; one physician visit per year during programme; vitamin D – 800 IU daily; calcium – ‘magistral formula’, 1000mg daily. - Pathway: proactive – BMD screening to diagnose osteoporosis - Resource/cost: BMD screening; physician labour; vitamin D and calcium tablets - Efficacy source: external meta-analyses – efficacy on hip fracture reduction [59]; on vertebral fracture [60]; on non-vertebral fractures [61] |
| Hirst (2016) | Transdermal buprenorphine vs. Tramadol for pain management | - Comparator: Tramadol - Component: (i) Transdermal buprenorphine – BuTrans 1.68 mg; 0.24 mg daily dose (1/7 of single patch); average of 107.18 days dosed per year; (ii) Tramadol – modified release non-propriety 100 mg; 240 mg daily dose; 107.18 days/year; BNF conversion ratio used to equate dosage of two medications. - Pathway: unclear – likely proactive because population is women 75+ using tramadol to treat moderate-to-severe pain; but screening not mentioned or costed - Resource/cost: see component. - Efficacy source: external case-control study for fracture odds ratio [62]; retrospective cohort study for medication average annual adherence rate [63]. |
| Honkanen (2006) | Hip protector | - Comparator: no hip protector or non-adherence to hip protector use - Component: hip protector efficacy gained only during hours it is worn during day; soft-shell version; functionally independent persons use less expensive pull-up model, dependent persons use more expensive wrap-around model; 3 or 5 pairs of protectors required per year in community (according to functional status), 4 or 7 in nursing home; protector use incurs health utility loss. - Pathway: unclear – likely proactive because study providing the adherence data [64] mentioned that women were referred from GPs to hospital outpatient clinic to discuss hip protector use; but referral process not modelled or costed. - Resource/cost: see component; depends on functional status and residence. - Efficacy source: efficacy – external RCT [65]; adherence (% of daily hours protector is worn) – external observational studies [64, 66]; utility loss [24]. |
| Howland (2015) | Multiple-component intervention – Matter of Balance lay-led version | - Comparator: usual care received by persons admitted to ED for fall - Component: ED fall patients advised by healthcare professional to participate in intervention; Matter of Balance lay-led version (MoB/VLL) – eight-session cognitive behavioural programme developed to reduce fear of falling and associated activity restriction; change perception of falls as something controllable; set goals to increase activity; reduce fall risk at home; exercise to increase strength and balance - Pathway: reactive – targets adults admitted to ED for fall - Resource/cost: cost of healthcare professionals promoting participation not included; per-participant cost estimate of MoB/VLL from previous cost analysis; deliberately excluded start-up cost included in Miller (2011). - Efficacy source: external RCT [67] |
| Ippoliti (2018) | Multifactorial intervention delivered by community nurses in mountainous areas | - Comparator: no intervention - Component: proactive engagement of community nurses with older persons living in mountainous areas of Italy; lifestyle changes (walking groups and low impact exercise); HAM (e.g., adequate lighting, bathroom handrails); active collaboration with family doctors to coordinate health and social care services (e.g., outpatient clinic visits, prescriptions, specialist services); community organisations and stakeholders to help identify needy older persons. - Pathway: proactive – relies on community organisations and stakeholders to identify needy older persons and promote uptake - Resource/cost: unclear whether community organisations are reimbursed by local health authority; staff labour; transport cost - Efficacy source: policy variable for break-even analysis |
| Johansson (2008) | Intersectoral intervention, Safe Seniors in Sundbyberg, including multifactorial and environmental interventions | - Comparator: usual care in community - Component: programme implemented in Sweden municipality with around 5,500 persons aged 65+; principle of community and intersectoral collaboration; components based on safety promotion and injury prevention methods; project team included full-time project coordinator, steering group (executives from regional healthcare management and elderly care organisations and representatives of public and voluntary organisations and businesses); (i) multifactorial intervention – safety education, group balance exercises delivered by PT; Tai Chi and other physical activities; HAM delivered by nurse and PT; (ii) environmental – safety surveillance in neighbourhoods; new routines in housing reconstruction (representatives from council, housing firm and tenants’ voluntary organisation inspected buildings and recommended new norms) - Pathway: (i) multifactorial intervention – likely self-referred, no mention of screening to refer eligible persons; (ii) environmental - Resource/cost: total cost of 2.5 million Swedish Krona over five years; primary collection of resource use data; coordinator labour cost for access; overheads costed based on hours of project work, assuming value of 20% of hourly labour costs for providing overheads; standardised cost of venues; labour wage cost according to profession; time costs for unpaid volunteers and participants valued at 35% of average wage; equipment (e.g., reconstruction costs, devices); only a portion of local stakeholder costs borne by the public sector - Efficacy source: internal quasi-experimental study |
| Lee (2013) | Vitamin D level screening and universal or targeted supplementation | - Comparator: no vitamin D level screening and no supplementation - Component: (i) universal supplementation – cholecalciferol 1,000 IU daily regardless of underlying 25-hydroxyvitamin D status; persons with vitamin D insufficiency gain benefit of falls risk reduction; persons with vitamin D deficiency gain no benefit due to insufficient dose; (ii) targeted supplementation – all persons screened for 25-hydroxyvitamin D levels; vitamin D dose varied according to level; no supplement for sufficient level; 1,000 IU daily for vitamin D insufficiency; 2,000-4,000 IU daily for vitamin D deficiency. - Pathway: proactive – even for universal supplementation because it is initiated by healthcare professional - Resource/cost: vitamin D screening reimbursement rate; supplements cost; physician time not costed. - Efficacy source: external meta-analyses (unclear referencing) [44, 68, 69] |
| Ling (2008) | HAM | - Comparator: no intervention - Component: Hana Program – installation of access ramps, minor floor repairs and grab-bars plus education on falls and follow-up by community volunteers; goal to promote independent living at home; targeted individuals with falls history and other falls risk factors - Pathway: unclear – targets those with falls history and other falls risk factors but unclear how they were identified and no cost of screening included. - Resource/cost: material; labour; community volunteer labour (not costed) - Efficacy source: external RCT (evaluated intervention includes not only HAM but also multidisciplinary multifactorial intervention) [52] |
| McLean (2015) | Group exercise | - Comparator: routine care and activity considered standard care - Component: NoFalls exercise programme – weekly one-hour group exercise (graded exercises for flexibility, leg strength, balance) for 15 weeks, supplemented by daily home exercises - Pathway: self-referred – investment in advertising (printed flyers, local newspapers in alternative scenario) - Resource/cost: staff labour; administration support; venue; music license fee; exercise equipment; consumables - Efficacy source: internal RCT [70] |
| Miller (2011) | Multiple-component intervention – Matter of Balance lay-led version | - Comparator: no intervention - Component: Matter of Balance lay-led version (MoB/VLL) – change perception of falls as something controllable; set goals to increase activity; reduce fall risk at home; exercise to increase strength and balance; eight two-hour sessions over 4-8 week period; start with training video and talk by qualified instructor on falls risk factors; later sessions involve exercise; training of lay leaders to lead sessions - Pathway: unclear – targets high-risk group but screening not mentioned or costed - Resource/cost: start-up and development cost (amortised over three years); coordinator fixed salary; labour; venues (donated by local organisations); supplies; training (master trainers and volunteers; fixed cost); total capacity of 10 classes with 140 participants (100 adherents). - Efficacy source: policy variable for break-even analysis |
| Mori (2017) | Exercise and oral bisphosphonate combined | - Comparator: cross-comparison – exercise alone, oral bisphosphonate alone or no intervention; exercise alone and no intervention strategies are dominated, hence comparison between oral bisphosphonate alone and combined strategy. - Component: combined strategy of falls prevention exercise for one year and oral bisphosphonate therapy for five years; (i) exercise – Otago programme; (ii) bisphosphonate therapy – initial dual-energy X-ray absorptiometry (DXA) measurement of femoral neck and lumbar spine; generic alendronate 70 mg once weekly for five years for those with osteoporosis (T-score -2.5 or less) - Pathway: unclear – likely proactive for oral bisphosphonate prescribing by healthcare professional; considers targeting osteoporotic patients only in alternative scenario but screening not mentioned or costed. - Resource/cost: (i) exercise – cost taken from Carande-Kulis (2015) plus time opportunity cost for exercise participants; (ii) bisphosphonate therapy – reimbursement rates for DXA and physician visits; medication - Efficacy source: exercise – external meta-analysis [71]; multiplicative efficacies for combined strategy. |
| Moriarty (2019) | Modification of inappropriately prescribed benzodiazepine and proton pump inhibitors | - Comparator: continued inappropriate prescription of benzodiazepine – use for four or more weeks – and proton pump inhibitors (PPIs) – use at maximal dose for longer than eight weeks. - Component: appropriate prescribing of benzodiazepine (complete withdrawal) and PPIs (use at maintenance dose); patients not shifted to appropriate prescribing after fall due to withdrawal symptoms for benzodiazepine and unlikely attribution of adverse events for PPIs – i.e., no reactive intervention pathway. - Pathway: unclear – likely proactive to identify inappropriately prescribed medication users, but screening not mentioned or costed; no reactive pathway. - Resource/cost: medication ingredient costs from reimbursement rates – annual cost at daily dose estimated; labour – pharmacist dispensing fee - Efficacy source: external RCTs for rates of discontinuation of inappropriately prescribed drugs – benzodiazepine [72]; PPI [73]. |
| Nshimyumu-kiza (2013) | BMD screening and physical activity and/or vitamin D and calcium supplementation as primary prevention of osteoporosis in women | - Comparator: cross-comparisons between primary and secondary osteoporosis prevention strategies and no prevention (absence of any specific national programme to initiate prevention) - Component: (i) primary prevention of osteoporosis for women not already engaged in the options recommended by the 2010 Canadian guidelines: (a) vitamin D and calcium supplementation; (b) physical activity (as simple was daily walking) promotion; (ii) secondary prevention of osteoporosis – universal screening programme using: (a) risk questionnaires (Simple Calculated Osteoporosis Risk Estimation (SCORE), Osteoporosis Risk Assessment Instrument (ORAI), Osteoporosis Self-Assessment Tool (OST)); (b) the Canadian Association of Radiologists and Osteoporosis Canada (CAROC) assessment tool, based on age, gender, BMD, prior fracture and prior use of glucocorticoids; (c) women categorised into low risk (<10% ten-year risk of fracture), moderate risk (10-20% risk) and high risk (>20% risk) based on CAROC thresholds; primary prevention for low-risk persons and osteoporosis treatment for high-risk. Following fracture, reactive intervention is initiated involving risedronate pharmacotherapy and vitamin D and calcium supplement. - Pathway: (i) primary prevention – proactive, because initiated only for those not currently engaged in adequate physical activity or vitamin D/calcium supplementation; under universal screening scenario, this assessment is made at screening; under primary prevention only scenario, this assessment process is not costed; but model includes cost of national physical activity promotion campaign, suggesting that there is a self-referred element; (ii) secondary prevention – proactive, always initiated after screening; (iii) reactive intervention – reactive, initiated after fracture incidence. - Resource/cost: (i) primary prevention – promotion campaign for physical activity access; vitamin D and calcium unit costs; cost of BMD screening; (ii) secondary prevention – cost of BMD screening; labour and pharmaceuticals. - Efficacy source: physical activity – external meta-analysis of prospective cohort studies [74]; vitamin D and calcium – external meta-analysis [60]; take the highest of the two efficacies. |
| OMAS (2008) | Multiple types | - Types: (i) exercise longer than six months; (ii) HAM; (iii) vitamin D and calcium supplementation; (iv) psychotropic medication withdrawal; (v) gait stabiliser - Comparator: no intervention - Component: (i) exercise – 26 group classes with PT for year, nine people per group; for mobile seniors without disability; (ii) HAM – one OT home visit (two hours) plus modifications; for frail seniors with disability; (iii) vitamin D and calcium – 1,000 IU vitamin D and 1,000 mg calcium daily; for older women with fracture risk factor(s); (iv) psychotropic medication withdrawal – one pharmacy consultation in year; for older psychotropic medication users; (v) gait stabiliser – single device replaced every year; for mobile seniors without disability. - Pathway: unclear – screening required for HAM, vitamin D and calcium, and psychotropic medication withdrawal; but not mentioned or costed. - Resource/cost: (i) exercise – labour (PT fee); (ii) HAM – labour; equipment; (iii) vitamin D and calcium – equipment; (iv) psychotropic medication withdrawal – labour; (v) gait stabiliser – equipment. - Efficacy source: internal meta-analysis |
| Pega (2016) | HAM | - Comparator: no intervention - Component: personalised assessment of injury hazards in the home (generally by OT) and systematic removal of these hazards (e.g., reducing tripping hazards, adding grab-bars, adding stairway handrails, improving home lighting); for older persons living in still unmodified private dwellings; evaluates alternative scenario of targeting subgroup with MA falls history. - Pathway: unclear – likely self-referred in base case but does not include cost of citywide invitation programme as in Wilson (2017); likely proactive in alternative scenario where HAM targeted at those who experience MA fall, but screening not mentioned or costed; mentions a further scenario in Appendix of prospective HAM provision for MA fallers at point of the fall, i.e., a reactive pathway. - Resource/cost: labour; equipment - Efficacy source: external meta-analysis [44] |
| Poole (2014) | Vitamin D supplementation | - Comparator: no supplementation - Component: licensed oral colecalciferol 800 IU daily (Desunim or Fultium-D3) - Pathway: unclear - Resource/cost: vitamin D daily dose cost - Efficacy source: external meta-analysis [75] |
| Poole (2015) | Vitamin D supplementation | - Comparator: no supplementation - Component: licensed oral colecalciferol 800 IU daily - Pathway: unclear - Resource/cost: vitamin D daily dose cost - Efficacy source: external meta-analysis [76] |
| PHE (2018) | (i) Otago home exercise; (ii) FaME group exercise; (iii) Group Tai Chi; (iv) HAM | - Comparator: control group in RCTs - Component: (i) Otago home exercise – initial assessment by PT or postural stability instructor to set starting level; supervised by trained instructor, with assumed mix of 50% PT, 40% technical assistant and 10% leisure service employees; 50% of staff require training; 10 contact hours for year (one initial visit, four follow-up visits, nine catch-up calls) (ii) FaME group exercise – initial assessment of ability; weekly one-hour sessions for 24 weeks; group of 10 participants; delivery staff mix of 20% PT, 45% technical assistant, 25% leisure service exercise instructors; 50% of staff require training; (iii) Tai Chi – 49 one-hour sessions twice per week; staff mix of 20% PT, 20% technical assistant and 60% self-employed instructors; 10 participants per group; 50% of staff require training; (iv) HAM – delivered only by OT; initial safety assessment at home and recommendations on required modifications plus follow-up visits; modifications and equipment – non-slip bathmat, stair rail, grab-rail, raised toilet seat, shower seat, rollator, wet rom conversion, move electrical cord - Pathway: unclear for Otago, FaME and Tai Chi – targeted high-risk group (34% of those aged 65+) but no screening mentioned or costed; reactive for HAM – targeted at hospitalised fallers (2% of those aged 65+) - Resource/cost: (i) Otago – labour, training, travel, equipment, evaluation; (ii) FaME – labour, training, travel, equipment, venue, evaluation; (iii) Tai Chi – labour, training, travel, equipment, venue, evaluation; (iv) HAM – labour, equipment, evaluation. - Efficacy source: (i) Otago home exercise – external meta-analysis [71]; (ii) FaME group exercise – external RCT [77]; (iii) Tai Chi – external meta-analysis [44]; (iv) HAM – external meta-analysis [44] |
| RCN (2005) | (i) Exercise; (ii) Multifactorial intervention | - Comparator: no intervention receipt - Component: falls risk screening for both interventions targeted at ‘high risk’ - Pathway: proactive – falls risk screening costed - Resource/cost: not stated - Efficacy source: internal meta-analysis |
| Sach (2007) | Expedited first-eye cataract surgery | - Comparator: ‘waiting list’ controls – surgery after 9-13 months - Component: first-eye cataract surgery (median time to surgery 27 days) for women aged 70+ with bilateral unoperated cataract, suitable for phacoemulsification - Pathway: proactive – involves referral to secondary care; resource item includes GP consultation (though not explicitly for screening) - Resource/cost: staff labour for screening; specialist cataract operation - Efficacy source: internal RCT |
| Sach (2010) | Expedited second-eye cataract surgery | - Comparator: ‘waiting list’ controls – surgery after median 316 days - Component: immediate second-eye cataract surgery (median time to surgery 30 days) for women aged 70+ who previously had successful first-eye cataract surgery and have a second operable cataract (baseline acuity of 6/12 or better, i.e., good vision in the operated eye) - Pathway: likely proactive as in Sach (2007), though referral not mentioned - Resource/cost: staff labour for screening; specialist cataract operation - Efficacy source: internal RCT |
| Smith (2016) | Falls risk screening and multifactorial intervention | - Comparator: cross comparison between falls risk cut-off levels for referral - Component: (i) falls risk prediction model generated from variables identified from multilevel logistic regressions (patients nested within GP) using Bayesian information criterion; variables – age group, sex, recent inpatient episodes, recent outpatient visits and frequency, recent A&E investigation, recent non-elective admission, fracture history recorded in GP, osteoporosis, falls history recorded in GP and hospital; COPD, stroke history, depression, mental health condition, asthma, urinary tract infection history, polypharmacy and drug number; (ii) components of multifactorial intervention not stated. - Pathway: proactive – falls risk screening followed by multifactorial intervention - Resource/cost: not stated; resource use/cost of using the falls risk prediction model not included. - Efficacy source: external meta-analysis [44] |
| Tannenbaum (2015) | Cognitive behavioural therapy (CBT) or sedative-hypnotic therapy for insomnia | - Comparator: no insomnia intervention; cross comparison - Component: (i) CBT – six-week course group therapy (private therapy in alternative scenario; (ii) sedative-hypnotic therapy – generic zolpidem tartrate 5 mg one tablet nightly (branded version in alternative scenario) - Pathway: proactive – both treatments initiated after GP diagnosis of insomnia which is costed. - Resource/cost: one-time GP consultation fee to diagnose insomnia; (i) CBT – reimbursement rate from the 2013 US National Government Services fee schedule; (ii) sedative-hypnotic therapy – reimbursement rate for monthly dispensing of generic zolpidem. - Efficacy source: external meta-analyses of prospective cohort studies – increased odds of falling under sedative-hypnotics relative to CBT [78]; increased odds under no insomnia intervention relative to CBT [79]. |
| Turner (2020) | Deprescribing of sedatives for insomnia by community pharmacists | - Comparator: usual care from pharmacists and GPs (no deprescribing) - Component: chronic users (>3 months of prescription claims) of sedatives for insomnia received an evidence-based educational brochure on sedative risks and alternative insomnia management strategies by community pharmacists; pharmacists provided evidence-based pharmaceutical opinion to patients’ GPs; GPs supervised deprescribing of sedatives - Pathway: proactive – initiated by community pharmacists - Resource/cost: pharmacist labour (including chronic sedative use identification); sedative medication cost. - Efficacy source: external RCT [80] |
| Velde (2008) | Withdrawal of fall-risk-increasing drugs (FRIDs) | - Comparator: no withdrawal after receiving geriatric assessment - Component: full geriatric assessment by geriatrician at geriatric outpatient clinic; medication list checked for FRID use (anxiolytics/hypnotics, antipsychotics, antidepressants, antihypertensives, anti-arrhythmics, nitrates and other vasodilators, digoxin, beta-andrenoceptor antagonist eye drops, analgesics, anticholinergics, antihistamines, anti-vertigo drugs, antihyperglycaemics); if fallen in past year, redundant FRID use stopped or reduced over one-month; prescribing physician consulted before change; patients consulted by telephone calls every two weeks during one-month withdrawal period; all other interventions postponed during one-month period - Pathway: proactive – patients referred to geriatric outpatient clinic; but this referral process not described or costed. - Resource/cost: staff labour – geriatric assessment and telephone calls; 72% added to labour cost to account for overheads and venue; pharmaceutical costs. - Efficacy source: internal observational study |
| Wilson (2017) | HAM | - Comparator: no intervention which is usual care - Component: personalised assessment of injury hazards in the home (generally by OT) and systematic removal of these hazards (e.g., reducing tripping hazards, adding grab-bars, adding stairway handrails, improving home lighting); for older persons living in still unmodified private dwellings; evaluates alternative scenario of targeting subgroup with MA falls history. - Pathway: self-referred in base case – includes cost of citywide invitation programme; proactive in alternative scenario – HAM targeted at those who experience MA fall, cost of invitation programme may account for screening cost. - Resource/cost: citywide invitation programme – fixed annual cost converted to per-participant cost; HAM – labour; material. - Efficacy source: external meta-analysis [44] |
| Wu (2010) | Multifactorial intervention | - Comparator: no intervention - Component: Falls Rehabilitation Program (FRP) is a proposed Medicare service modelled after a typical multifactorial intervention – FRA (medications, vision, gait, mobility, balance, lifestyle, blood pressure, function, home hazards) by physician at usual office visit followed by group exercise (eight sessions);^2^ targeted at Medicare beneficiaries who have fallen within the previous 12 months; Medicare physician receive additional reimbursement in addition to usual office visit rate plus single follow-up visit to assess compliance. - Pathway: proactive – FRP initiated (i.e., patient screened) by physician at usual office visit; but screening not costed. - Resource/cost: reimbursement rates for FRP (in addition to the physician’s usual office visit fees), group exercise sessions and follow-up visit - Efficacy source: external meta-analysis [81] |
| Zarca (2014) | Vitamin D supplementation – universal or one of two targeting strategies | - Comparator: no supplementation; cross-comparison between strategies - Component: (i) ‘Treat without check’ – universal vitamin D supplementation; (ii) ‘Treat, then check’ – immediate universal supplementation, then screen for 25(OH) vitamin D serum level three months later for subsequent treatment adaptation; (iii) ‘Screen and treat’ – screen for vitamin D insufficiency then treat. Maximum number of check and adaptation cycles for strategies (ii) and (iii) set at two (adaptation followed French guidelines); thereafter, individuals received vitamin D quarterly until death. For vitamin D deficiency: four 100,000 UI doses at two-week intervals followed by one 100,000 UI dose every quarter. For vitamin D insufficiency: two to three 100,000 UI doses at two-week intervals followed by one 100,000 UI dose every quarter. - Pathway: proactive – even universal supplementation initiated by professional - Resource/cost: cost per screening – one GP visit and one 25(OH) vitamin D test; cost per 100,000 UI vitamin D dose. - Efficacy source: effect of vitamin D supplementation on vitamin D level – external RCT [82]; relationship between vitamin D level and hip fracture risk – external meta-analysis [61] |
| **Abbreviation:** BMD: bone mass density; BNF: British National Formulary; CSP: Chartered Society of Physiotherapy; ED: emergency department; FaME: Falls Management Exercise; FRA: falls risk assessment; FRAT: falls risk assessment tool; HAM: home assessment and modification; MA fall: fall requiring medical attention; OMAS: Ontario Medical Advisory Secretariat; OT: occupational therapist; PHE: Public Health England; PT: physiotherapist; QTUG: quantitative timed-up-and-go; RCN: Royal College of Nursing; RCT: randomised controlled trial; TUG: timed-up-and-go  ^1^ See Table 2 in main manuscript text for study references; parenthesised number refers to the number of models included in the table.  ^2^ Text mentions more treatments including medication modification, behavioural recommendations, HAM and rehabilitation therapy; but intervention costing only includes group exercise. | | |

# Intervention resource-use and cost

| **Table A8.** Intervention resource-use and cost from the public sector perspective in included decision models. | | | | | | | | | | | |
| --- | --- | --- | --- | --- | --- | --- | --- | --- | --- | --- | --- |
|  |  | **Auxiliary implementation resources^1^** | | | | **Therapeutic resources^1^** | | | | | **Cost** |
| **Study label (n=46)^2^** | **Intervention** | Initial access | Compliance & sustain. | Falls risk screening | Set-up; other | Staff labour | Training | Staff transport | Venue & Overhead | Health tech. & equipment | Summary |
| ***Exercise*** | | | | | | | | | | | |
| Alhambra-Borras (2019) | Group exercise |  |  |  |  | ˟ |  |  |  |  | PP |
| Carande-Kulis (2015) | Home exercise | ˟ |  |  |  | ˟ | ˟ | ˟ |  |  | PP |
|  | Tai Chi (group) | ˟ |  |  |  | ˟ | ˟ |  |  |  | PP |
| CSP (2016) | FRS and physiotherapy (group or home) |  |  | Not costed |  | ˟ |  |  |  |  | Per staff; Total |
| Day (2009) | Home exercise^3^ | ˟ |  | ˟ |  | ˟ | ˟ | ˟ |  | ˟ | PP |
| Day (2009); (2010) | Tai Chi (group) | ˟ | ˟ |  |  | ˟ |  |  | VN | ˟ | PP |
| Deverall (2018) | Home exercise | ˟ |  |  |  | ˟ |  |  | OH | ˟ | PP |
|  | Group exercise (peer-led) |  |  |  |  | ˟ | ˟ | ˟ |  |  | PP |
| Franklin (2019) | FRS and home exercise |  |  | ˟ | Set-up; Eval. cost | ˟ | ˟ | ˟ |  | ˟ | Fixed & PP |
|  | FRS and group exercise (FaME) |  |  | ˟ | Set-up; Eval. cost | ˟ | ˟ | ˟ | VN&OH | ˟ | Fixed & PP |
|  | FRS and Tai Chi (group or home) |  |  | ˟ | Set-up; Eval. cost | ˟ | ˟ | ˟ | VN&OH | ˟ | Fixed & PP |
| Frick (2010) | Muscle & balance training |  |  |  |  | ˟ |  | ˟ | OH |  | PP |
|  | Tai Chi |  |  |  |  | ˟ |  | ˟ | OH |  | PP |
| Hektoen (2009) | Home exercise | ˟ |  |  |  | ˟ |  |  |  | ˟ | PP |
| McLean (2015) | Group + home exercise | ˟ |  |  |  | ˟ |  |  | VN&OH | ˟ | PP |
| Mori (2017) | Home exercise | ˟ |  |  |  | ˟ | ˟ | ˟ |  |  | PP |
| OMAS (2008) | Group exercise |  |  |  |  | ˟ |  |  |  |  | PP |
| PHE (2018) | Home exercise |  |  |  | Eval. cost | ˟ | ˟ | ˟ |  | ˟ | PP |
|  | Group exercise (FaME) |  |  |  | Eval. cost | ˟ | ˟ | ˟ | VN&OH | ˟ | PP |
|  | Tai Chi (group or home) |  |  |  | Eval. cost | ˟ | ˟ | ˟ | VN&OH | ˟ | PP |
| Nshimyumukiza (2013) | BMD screening and physical activity | ˟ |  | BMD |  |  |  |  |  |  | PP |
| RCN (2005) | FRS and exercise (unspecified) |  |  | ˟ |  | Not specified | | | | | PP |
| ***Home assessment and modification (HAM)*** | | | | | | | | | | | |
| Agartioglu (2020) | HAM |  |  |  | ˟ | ˟ |  | ˟ |  | ˟ | PP |
| Day (2009) | HAM (reactive)^3^ |  |  | Not costed |  | ˟ |  | ˟ |  | ˟ | PP |
| PHE (2018) | HAM (reactive) |  |  |  | Eval. cost | ˟ |  |  | OH | ˟ | PP |
| Franklin (2019) | FRS and HAM |  |  | ˟ | Set-up; Eval. cost | ˟ |  |  | OH | ˟ | Fixed & PP |
| Frick (2010) | HAM |  |  |  |  | ˟ |  | ˟ | OH |  | PP |
| Ling (2008) | HAM |  |  |  |  | ˟ |  |  |  | ˟ | PP |
| OMAS (2008) | HAM |  |  |  |  | ˟ |  |  |  | ˟ | PP |
| Pega (2016) | HAM |  |  |  |  | ˟ |  |  |  | ˟ | PP |
| Wilson (2017) | HAM | ˟ |  | ˟ |  | ˟ |  |  |  | ˟ | PP |
| ***Medication review and change*** | | | | | | | | | | | |
| Day (2009) | FRS and psychotropic withdrawal^3^ |  |  | ˟ |  | ˟ |  |  |  | ˟ | PP |
| Frick (2010) | Psychotropic withdrawal |  |  |  |  | ˟ |  |  | OH | Not specified | PP |
| Hirst (2016) | Pain medication change |  |  |  |  |  |  |  |  | ˟ | PP |
| Moriarty (2019) | Change in PIP benzodiazepine and PPI |  |  |  |  | ˟ |  |  |  | ˟ | PP |
| OMAS (2008) | Psychotropic withdrawal |  |  |  |  | ˟ |  |  |  | Not specified | PP |
| Tannenbaum (2015) | FRS and insomnia drug vs. CBT |  |  | ˟ |  |  |  |  |  | ˟ | PP |
| Turner (2020) | Sedatives withdrawal |  |  | ˟ |  | ˟ |  |  |  | ˟ | PP |
| Velde (2008) | FRID withdrawal |  |  |  |  | ˟ |  |  | OH | ˟ | PP |
| ***Expedited cataract surgery*** | | | | | | | | | | | |
| Boyd (2020) | Expedited and routine surgeries |  |  |  |  | ˟ |  |  |  | ˟ | PP |
| Church (2011); (2012) | Expedited surgery |  |  |  |  | ˟ |  |  |  | ˟ | PP |
| Sach (2007); (2010) | Expedited surgery |  |  | ˟ |  | ˟ |  |  |  | ˟ | Total |
| ***Vitamin D supplementation*** | | | | | | | | | | | |
| Lee (2013) | Vit. D screening and supplementation |  |  | Vit. D |  |  |  |  |  | ˟ | Unit cost |
| Zarca (2014) | Vit. D screening and supplementation |  |  | Vit. D |  | ˟ |  |  |  | ˟ | Unit cost |
| Frick (2010) | Vit. D |  |  |  |  | ˟ |  |  | OH | Not specified | PP |
| Hiligsmann (2014) | Vit. D screening and Vit. D + calcium |  |  | BMD |  | ˟ |  |  |  | ˟ | PP |
| Nshimyumukiza (2013) | Vit. D screening and Vit. D + calcium |  |  | BMD |  |  |  |  |  | ˟ | PP |
| OMAS (2008) | Vit. D + calcium |  |  |  |  |  |  |  |  | ˟ | PP |
| Poole (2014); (2015) | Vit. D |  |  |  |  |  |  |  |  | ˟ | PP |
| ***Other single-component interventions*** | | | | | | | | | | | |
| Day (2009) | Cardiac pacing^3^ |  |  | ˟ |  | ˟ |  |  |  | ˟ | PP |
| Honkanen (2006) | Hip protector |  |  |  |  |  |  |  |  | ˟ | PP |
| OMAS (2008) | Gait stabiliser |  |  |  |  |  |  |  |  | ˟ | PP |
| ***Multifactorial intervention and risk assessment*** | | | | | | | | | | | |
| Albert (2016) | MF int. |  | ˟ |  |  | ˟ | ˟ |  |  |  | PP |
| Comans (2009) | MF int. |  |  |  |  | ˟ |  | ˟ |  | ˟ | Fixed & PP |
| Day (2009) | FRS and MF int.^3^ | ˟ |  | ˟ |  | ˟ |  |  | OH | ˟ | PP |
| Eldridge (2005) | FRS and falls clinic MF int. or balance and gait treatment | ˟ | ˟ | ˟ | Set-up | ˟ |  |  | VN&OH | ˟ | Fixed & PP |
| Frick (2010) | MF int. |  |  |  |  | ˟ |  | ˟ | OH |  | PP |
|  | MF int. for high risk |  |  |  |  | ˟ |  | ˟ | OH |  | PP |
| Ippoliti (2018) | MF int. |  |  |  |  | ˟ |  | ˟ |  |  | Total |
| RCN (2005) | FRS and MF int. |  |  | ˟ |  | Not specified | | | | | PP |
| Smith (2016) | FRS and MF int. |  |  | Not costed |  | Not specified | | | | | PP |
| Wu (2010) | MF int. |  |  |  |  | Not specified | | | | | PP |
| Church (2011); (2012) | MF risk assessment |  |  |  |  | ˟ |  |  |  |  | PP |
| ***Multiple-component intervention*** | | | | | | | | | | | |
| Beard (2006) | MC (intersectoral) int.^4^ | ˟ |  |  |  | ˟ |  |  | OH |  | Annual total |
| Carande-Kulis (2015) | Stepping On^5^ | ˟ | ˟ |  |  | ˟ | ˟ |  | VN |  | PP |
| Howland (2015) | MoB (lay-led) | Not costed |  |  |  | Not specified | | | | | PP |
| Johansson (2008) | MC (intersectoral) int.^6^ | ˟ | ˟ |  |  | ˟ |  |  | VN&OH | ˟ | Total |
| Miller (2011) | MoB (lay-led) |  | ˟ |  | Set-up | ˟ | ˟ |  |  | ˟ | PP |
| **Abbreviation:** BMD: bone mineral density; CBT: cognitive behavioural therapy; Compliance & sustain.: compliance and sustainability; CSP: Chartered Society of Physiotherapy; FaME: Falls Management Exercise programme; FRID: fall-risk increasing drug; FRS: falls risk screening; Health tech.: health technology; Int.: intervention; MC: multiple-component; MF: multifactorial; MoB: Matter of Balance; OH: overhead; OMAS: Ontario Medical Advisory Secretariat; PHE: Public Health England; PIP: potential inappropriately prescribed; PP: per participant; PPI: proton pump inhibitor; PT: physiotherapy; RCN: Royal College of Nursing; VN: venue  ^1^ ‘Not costed’ refers to cases where resource use is mentioned but not costed. ‘Not specified’ refers to cases where summary cost is reported but component resources are not, requiring inference on the likely components.  ^2^ See Table 2 in main manuscript text for study references; parenthesised number refers to the number of models included in the table.  ^3^ Same intervention resource use and cost data were used by Church (2011) and (2012).  ^4^ Intervention included individually tailored education, HAM and exercise and public space safety improvement.  ^5^ Church (2011) and (2012) cite the same RCT as the source of intervention resource use and cost; but the final PPY mean costs differ.  ^6^ Intervention included individually tailored education, group balance exercises, Tai Chi, other physical activities and HAM, neighbourhood hazard removal and housing reconstruction. | | | | | | | | | | | |

# Assessing parameter uncertainty

| **Table A9.** Parameters explored in deterministic sensitivity analysis and probabilistic sensitivity analysis presentation methods.^1^ | | | |
| --- | --- | --- | --- |
| **Study label (n=46)^2^** | **DSA parameters** | | **PSA presentation methods** |
|  | **Falls epidemiology** | **Falls prevention intervention** |  |
| Agartioglu (2020) | (1) Fracture HC cost; (2) Head injury HC cost |  | (1) CEAC; (2) CEAF; (3) Scatter |
| Albert (2016) | (1) Utility; (2) HC cost |  | (1) CEAC; (2) VoI |
| Alhambra-Borras (2019) | *None* | | |
| Beard (2006) | *None* | | |
| Boyd (2020) | (1) Falls risk; (2) Initial falls history; (3) Hospital fall risk; (4) Fatal fall risk; (5) Utility; (6) HC cost; (7) LTC BG risk | (1) Int. cost; (2) Efficacy | (1) 95% UI |
| Carande-Kulis (2015) | *None* | | |
| CSP (2016) | *None* | | |
| Church (2011) | (1) Falls risk; (2) Utility | (1) Int. cost; (2) Efficacy; (3) Falls rate multiplier |  |
| Church (2012) | (1) Falls risk; (2) Utility | (1) Int. cost; (2) Efficacy; (3) Falls rate multiplier | (1) CEAC |
| Comans (2009) | (1) Falls rate per faller; (2) HC cost | (1) Int. cost |  |
| Day (2009); (2010) | (1) Hospital fall risk | (1) Int. cost; (2) Uptake; (3) Efficacy |  |
| Deverall (2018) | (1) Falls risk; (2) Initial falls history; (3) Hospital fall risk; (4) Fatal fall risk; (5) Utility; (6) HC cost; (7) LTC BG risk | (1) Int. cost; (2) Persistence; (3) Efficacy | (1) CEAC; (2) 95% UI |
| Eldridge (2005) |  |  | (1) CEAC |
| Farag (2015) | (1) Falls risk; (2) Hospital fall risk; (3) LTC fall risk; (4) Utility; (5) HC cost; (6) LTC cost |  | (1) CEAC |
| Franklin (2019) |  |  | (1) CEAC; (2) CE prob. |
| Frick (2010) |  |  | (1) CEAC |
| Hektoen (2009) | *None* | | |
| Hiligsmann (2014) | (1) Falls risk; (2) Utility; (3) HC cost | (1) Int. cost | (1) CEAC |
| Hirst (2016) | (1) Utility; (2) HC cost | (1) Int. cost; (2) Efficacy | (1) CEAC |
| Honkanen (2006) | (1) Falls risk; (2) LTC fall risk; (3) Fatal fall risk; (4) HC cost; (5) BG health transition risk; (6) BG mortality risk; (7) LTC BG risk; (8) Com. care cost | (1) Int. cost; (2) Adherence; (3) Persistence; (4) Efficacy | (1) CE prob. |
| Howland (2015) | *None* | | |
| Ippoliti (2018) | *None* | | |
| Johansson (2008) |  |  | (1) Scatter |
| Lee (2013) | (1) Falls risk; (2) MA falls risk; (3) Utility; (4) HC cost; (5) BG mortality risk | (1) Int. cost; (2) Efficacy | (1) CE prob. |
| Ling (2008) | *None* | | |
| McLean (2015) |  |  | (1) CEAC; (2) CE prob. |
| Miller (2011) | *None* | | |
| Mori (2017) | (1) Fracture risk; (2) Recurrent fracture risk; (3) Osteoporosis risk; (4) Fracture risk with osteoporosis; (5) Excess mortality risk; (6) Utility; (7) BG mortality risk | (1) Int. cost; (2) Exercise time opportunity cost; (3) Efficacy | (1) CE prob. |
| Moriarty (2019) | (1) HC cost | (1) Int. cost | (1) Scatter |
| Nshimyumukiza (2013) |  |  | (1) CEAC; (2) CE prob. |
| OMAS (2008) | *None* | | |
| Pega (2016) | (1) Falls risk; (2) Initial falls history; (3) Hospital fall risk; (4) Fatal fall risk; (5) Utility; (6) HC cost; (7) LTC BG risk; (8) Rate of moving house | (1) Int. cost | (1) 95% UI |
| Poole (2014); (2015) | *None* | | |
| PHE (2018) | (1) Discharge destination after hospital fall; (2) Utility; (3) HC cost |  |  |
| RCN (2005) |  |  | (1) Scatter |
| Sach (2007); (2010) |  |  | (1) CEAC |
| Smith (2016) |  |  | (1) 95% UI |
| Tannenbaum (2015) | (1) Falls risk; (2) Recurrent fall risk; (3) Utility; (4) HC cost | (1) Int. cost | (1) CEAC |
| Turner (2020) |  |  | (1) CEAC; (2) CE prob. |
| Velde (2008) |  |  | (1) 95% UI |
| Wilson (2017) | (1) Falls risk; (2) Initial falls history; (3) Hospital fall risk; (4) Fatal fall risk; (5) Utility; (6) HC cost; (7) LTC BG risk; (8) Rate of moving house | (1) Int. cost; (2) Uptake; (3) Efficacy | (1) 95% UI |
| Wu (2010) | (1) Falls risk; (2) Recurrent fall risk | (1) Efficacy |  |
| Zarca (2014) | (1) Mean baseline Vit. D level; (2) Risk of recurrent fracture; (3) Excess mortality; (4) HC cost | (1) Int. cost; (2) Adherence; (3) Efficacy | (1) CEAC; (2) Scatter; (3) CE prob. |
| **Abbreviation:** BG: background; CE: cost-effectiveness; CEAC: cost-effectiveness acceptability curve; CEAF: cost-effectiveness acceptability frontier; Com.: comorbidity; CSP: Chartered Society of Physiotherapy; DSA: deterministic sensitivity analysis; HC: healthcare; Int.: intervention; LTC: long-term care; MA falls: falls requiring medical attention; OMAS: Ontario Medical Advisory Secretariat; PHE: Public Health England; prob.: probability; PSA: probabilistic sensitivity analysis; RCN: Royal College of Nursing; UI: uncertainty interval; VoI: value of information.  ^1^ To distinguish between assessment of parameter uncertainty in DSA and evaluation of alternative scenarios (see Table A10), attention was paid to studies’ descriptions of the purpose of their sensitivity analyses, though these varied in clarity. For example, if the parameter range assessed in DSA denoted the 95% confidence interval then the analysis concerned parameter uncertainty.  ^2^ See Table 2 in main manuscript text for study references; parenthesised number refers to the number of models included in the table. | | | |

# Scenario analyses

| **Table A10.** Scenarios evaluated by models in sensitivity analysis | | | |
| --- | --- | --- | --- |
| **Study label (n=46)^1^** | **Falls epidemiology** | **Falls prevention intervention** | **Evaluation framework** |
| Agartioglu (2020) | (1) Falls risk | (1) Efficacy: pessimistic, optimal, optimistic |  |
| Albert (2016) | *None* | | |
| Alhambra-Borras (2019) |  |  | (1) Discount rate |
| Beard (2006) | (1) Costs included: narrow HC vs. wide HC vs. societal | (1) Control for secular falls trend in effectiveness estimation | (1) Perspective: HC vs. Societal |
| Boyd (2020) |  | (1) Efficacy: vision benefit only from cataract surgery | (1) Time horizon; (2) Discount rate |
| Carande-Kulis (2015) | (1) Higher HC cost per fall for 80+ | (1) Lower int. training cost; (2) Efficacy break-even analysis |  |
| CSP (2016) |  | (1) Access rate |  |
| Church (2011); (2012) | (1) Baseline age; (2) No fear of falling |  |  |
| Comans (2009) |  | (1) Uptake break-even analysis |  |
| Day (2009) |  | (1) Private co-payment for int. | (1) Time horizon |
| Day (2010) |  | (1) Private co-payment for int. |  |
| Deverall (2018) |  |  | (1) Equity analysis; (2) Discount rate |
| Eldridge (2005) |  | (1) Int. cost; (2) Uptake rate |  |
| Farag (2015) |  | (1) Int. cost; (2) Uptake rate; (3) Efficacy |  |
| Franklin (2019) | (1) Utility | (1) TUG/QTUG efficacy; (2) Uptake | (1) Perspective: HC vs. H&SC |
| Frick (2010) | *None* | | |
| Hektoen (2009) | (1) Risk of injury break-even analysis | (1) Efficacy break-even analysis |  |
| Hiligsmann (2014) |  | (1) Sustain rate; (2) Efficacy on mortality risk | (1) Discount rate |
| Hirst (2016) | (1) Fracture risk; (2) Fracture cost | (1) Int. process cost; (2) Adherence rate |  |
| Honkanen (2006) | (1) Fracture risk | (1) Int. process disbenefit; (2) Int. cost; (3) Int. effectiveness |  |
| Howland (2015) |  | (1) Uptake rate; (2) State level scale-up |  |
| Ippoliti (2018) |  | (1) Efficacy break-even point |  |
| Johansson (2008) | (1) Fracture risk; (2) Mortality risk; (3) HC cost; (4) No health/economic effects after 1^st^ yr.; (5) Utility; (6) Loss of unpaid productivity; (7) Consumption cost of added years | (1) Int. cost; (2) Efficacy – break-even analysis, alternate estimate | (1) Discount rate |
| Lee (2013) | *None* | | |
| Ling (2008) | *None* | | |
| McLean (2015) | (1) HC cost | (1) Int. cost – different professional; (2) Int. cost – no venue or equipment cost; (3) Int. cost – advertising cost; (4) Efficacy threshold analysis | (1) Discount rate; (2) Handling missing data |
| Miller (2011) |  | (1) Efficacy break-even analysis | (1) Perspective: HC vs. Societal |
| Mori (2017) | (1) Excess mortality for vertebral fracture | (1) Longer exercise maintenance; (2) Exercise only for osteoporosis patients | (1) Discount rate |
| Moriarty (2019) |  | (1) Int. cost threshold analysis; (2) Adherence rate | (1) Discount rate |
| Nshimyumukiza (2013) | (1) Fracture hospital cost; (2) Fracture LTC admission risk; (3) Excess mortality risk; (4) Utility | (1) Uptake rates; (2) Reactive access to osteoporosis pathway; (3) Compliance and sustainability of osteoporosis pathway; (4) Efficacy of primary prevention; (5) Efficacy of osteoporosis pathway | (1) Discount rate |
| OMAS (2008) | *None* | | |
| Pega (2016) |  | (1) Int. cost reduced from economies of scale; (2) Efficacy heterogeneity; (3) Perfect efficacy; (4) Shorter efficacy duration | (1) Equity analysis; (2) Discount rate |
| Poole (2014); (2015) | *None* | | |
| PHE (2018) | (1) Falls rate threshold analysis; (2) MA falls risk threshold analysis | (1) Efficacy threshold analysis |  |
| RCN (2005) | *None* | | |
| Sach (2007); (2010) | (1) High cost outliers removed | (1) Int. cost threshold analysis; (2) Different efficacy path (immediate vs. gradual gain over 6 months) | (1) Perspective: HC vs. Societal; (2) Time horizon; (3) Discount rate |
| Smith (2016) |  | (1) Different falls risk cut-off levels for referral to treatment |  |
| Tannenbaum (2015) |  |  | (1) Time horizon |
| Turner (2020) |  | (1) Int. implemented at primary care, not community pharmacy; (2) Int. cost increase; (3) Lower efficacy on deprescribing |  |
| Velde (2008) | (1) Injurious falls risk | (1) National level scale-up |  |
| Wilson (2017) |  | (1) Int. cost reduced from economies of scale; (2) Shorter efficacy duration | (1) Equity analysis; (2) Time horizon; (3) Discount rate |
| Wu (2010) | (1) Magnitude of additional HC cost for recurrent faller; (2) Proportion of fall HC cost averted | (1) Uptake rate; (2) Greater reach of exercise; (3) Int. cost; (4) Efficacy threshold analysis | (1) Perspective – proportion of HC cost accruing to Medicare; (2) Perspective – HC vs. Societal |
| Zarca (2014) |  |  | (1) Discount rate |
| **Abbreviation:** CSP: Chartered Society of Physiotherapy; HC: healthcare; H&SC: health and social care; Int.: intervention; LTC: long-term care; MA fall: fall requiring medical attention; OMAS: Ontario Medical Advisory Secretariat; PHE: Public Health England; RCN: Royal College of Nursing; TUG/QTUG: (quantified) timed-up-and-go  ^1^ See Table 2 in main manuscript text for study references; parenthesised number refers to the number of models included in the table. | | | |

# Evaluation outcomes for non-general population and/or non-lifetime models

| **Table A11.** Evaluation outcomes for non-lifetime and/or non-general population models. | | | | |
| --- | --- | --- | --- | --- |
| **Study label (n=34)^1^** | **Target population** | **Analysis; Perspective; Time horizon** | **Intervention [comparator]** | **Evaluation outcomes^2^** |
| Agartioglu (2020) | CD adults aged 65+ | CEA; Public sector; 1 year | HAM [UC] | ***Ratio***: Unclear – appears to report per-participant intervention cost rather than ICERs.  ***Aggregate***: Not reported  ***Parameter uncertainty***: DSA – results robust to cost variations; PSA – CEAC, CEAF, scatter.  ***Scenarios***: Results robust to change in baseline risk; results reported by efficacy scenarios (pessimistic, optimal, optimistic). |
| Albert (2016) | CD adults aged 50+ (mean age 75.5) | CUA; Public sector; 1 year | MF int. [UC] | ***Ratio***: Intervention had higher health gain and lower cost (dominated) comparator; net saving of US$846 per participant; average EQ-5D gain of 0.008 per participant.  ***Aggregate***: Per-participant results can be scaled up to quasi-experimental study sample size.  ***Parameter uncertainty***: DSA – results robust to treatment cost and utility variations, utility had the greatest impact; PSA – CEAC, no uncertainty for utility parameter favoured intervention.  ***Scenarios***: No analysis |
| Alhambra-Borras (2019) | CD adults aged 65+ at high falls risk or frail with no severe physical or cognitive limitation | CUA; Public sector; Lifetime | Exercise [UC] | ***Ratio*:** Intervention had higher health gain and lower cost (dominated) comparator.  ***Aggregate***: Total cost saving of US$73,564 and QALY gain of 0.513 for intervention group relative to control group – no extrapolation to larger population.  ***Parameter uncertainty***: No analysis  ***Scenarios***: No discounting had little impact on decision. |
| Beard (2006) | CD adults aged 60+ | CBA, ROI; Public sector, Societal; 5 years | MC (intersectoral) int.^3^ [UC] | ***Ratio***: ROI of 8.5:1 from state government perspective (hospitalisation cost only); 13.7:1 from national government perspective (direct healthcare costs); 20.6:1 from societal perspective (direct healthcare costs + intangible cost of falls + participant costs for exercise)  ***Aggregate***: Reports total net economic saving  ***Parameter uncertainty***: No analysis  ***Scenarios***: Range of costs included in public healthcare perspective; Public vs. societal perspective; Minimising the proportion of the secular decline in fall-related healthcare cost in intervention region (relative to comparator regions) that can be attributed to intervention lowered ROI to 6.3:1 (hospitalisation cost only). |
| Boyd (2020) | Adults aged 65+ | CUA; Public sector; Lifetime | Cataract surgery (expedited, routine) [NR] | ***Ratio***: Expedited vs. routine surgery produced ICER of US$8,427 per QALY; Routine vs. no surgery produced ICER of US$3,482 per QALY; Routine vs. no surgery (vision improvement benefit only) produced ICER of US$7,848 per QALY.  ***Aggregate***: Reports aggregate health gain and incremental cost.  ***Parameter uncertainty***: DSA – ICER most sensitive to variations in efficacy, utility loss from cataract, and expedited surgery cost; PSA – reports 95% uncertainty interval for all outcomes.  ***Scenarios***: Expedited surgery cost-effective relative to routine surgery under 10-year and 20-year time horizons, 0% and 6% discount rates and private sector surgery costs. |
| Carande-Kulis (2015) | CD adults aged 65+ | ROI; US health insurance payer; 1 year | Exercise (2 forms); MC int. (Stepping On) [NR] | ***Ratio***: ROI of 1.36:1 for Otago exercise; 2.27:1 for Otago exercise high-risk subgroup; 6.09:1 for Tai Chi; 1.64:1 for Stepping On.  ***Aggregate***: Not reported  ***Parameter uncertainty***: No analysis  ***Scenarios***: All interventions can break-even with lower efficacy; 50% increase in falls economic cost for Otago exercise high-risk subgroup increased ROI from 2.27:1 to 3.40:1; No difference in results under cheaper intervention cost of online training. |
| CSP (2016) | CD adults aged 65+ | ROI; Public sector; 1 year | FRS + Exercise (physiotherapy) [NR] | ***Ratio***: ROI of 1.69:1 for combined physiotherapy form (20% group physiotherapy; 87% TUG test efficacy; 11% referral rate) in England.  ***Aggregate***: Reports total intervention cost and total fall-related healthcare savings.  ***Parameter uncertainty***: No analysis  ***Scenarios***: Same ROI when access level increased from 11% to 100%; Interactive Excel platform allows users to change parameters. |
| Church (2011) | CD adults aged 65+ (separate model for residential care) | CEA, CUA; Public sector; 10 years | Exercise (3 forms); MC int.; MF int.; MRA; Exp. cataract surgery; Med. modification; Cardiac pacing [NR] | ***Ratio***: (CUA results) ICER per QALY vs. no intervention: Tai Chi US$39,328; Group exercise US$63,765; Home exercise US$84,307; MC int. US$65,011; MF int. US$114,045; MRA US$150,736; Expedited cataract surgery US$1,937; Psychotropic medication withdrawal US$14,534; Cardiac pacing US$70,331.  ***Aggregate***: Not reported  ***Parameter uncertainty***: DSA – (For group exercise only) Intervention cost, efficacy and baseline age were the most important determinants of ICER for group exercise. No PSA.  ***Scenarios***: No fear of falling had the largest impact on group exercise ICER among parameter changes (for DSA and scenarios). |
| Comans (2009) | CD adults aged 65+, falls history in past 6 months or gait/functional decline and cognitively intact | ROI; Societal; 1 year | MF int. (2 forms) [NR] | ***Ratio***: Centre-based MF int. needs 57 clients per year to break even; Home-based MF int. 78. Both services have annual capacity of 300, hence likely to return positive ROI.  ***Aggregate***: Not reported  ***Parameter uncertainty***: DSA – Both MF int. forms were not able to break even if HC cost of falls was reduced by 25% or if baseline falls rate was reduced from 6 to 1 per year. No PSA.  ***Scenarios***: Uptake (number of clients) required for break-even (see *Ratio*) |
| Day (2009) | CD adults aged 50+ (age and characteristics differ by intervention type) | CEA; Public sector, Societal; 1 year | Exercise (2 forms); HAM; MF int.; Med. modification; Cardiac pacing [NR] | ***Ratio***: ICER per fall prevented relative to no intervention – Home exercise US$3,841; Tai Chi US$989; HAM US$383; MF int. US$758; Psychotropic med. withdrawal US$459; Cardiac pacing US$2,384  ***Aggregate***: Reports total number of falls prevented and total cost per intervention.  ***Parameter uncertainty***: DSA – Explored impacts of variations in risk of hospitalised fall, intervention uptake, intervention cost and efficacy. No PSA.  ***Scenarios***: Two-year horizon for home exercise produced ICER of US$2,677 per fall prevented; Five-year horizon for cardiac pacing produced ICER of US$476 per fall prevented. Explored scenarios of private co-payments for Tai Chi which improved the ICER for public sector. |
| Day (2010) | CD adults aged 70+ | CEA; Public sector, Societal; 1 year | Exercise (Tai Chi) [NR] | ***Ratio***: Tai Chi produced ICER of US$4,045 per fall prevented relative to no intervention.  ***Aggregate***: Reports total number of falls prevented and total cost per intervention.  ***Parameter uncertainty***: DSA – Explored impacts of variations in risk of hospitalised fall, intervention uptake, intervention cost and efficacy. No PSA.  ***Scenarios***: Private co-payments for Tai Chi improved the ICER for public sector. |
| Franklin (2019) | CD adults aged 65+ | CUA; Public sector (2 types); 2 years | FRS + Exercise (3 forms) or HAM [NR; Cross-comparison] | ***Ratio***: (Results for QTUG pathway vs. no intervention for age 65-89 – see Franklin (2019) for results for TUG pathway and age subgroups) QTUG and Otago exercise produced ICER of US$2,925 per QALY; QTUG and FaME dominated no intervention; QTUG and Tai Chi produced ICER of US$20,450 per QALY; QTUG and HAM dominated no intervention.  ***Aggregate***: Reported incremental HC costs and QALY at GP practice cohort level.  ***Parameter uncertainty***: PSA – CEAC; see Table 2 in Franklin (2019) for CE probabilities.  ***Scenarios***: See Appendix Tables S9 to S11 on impacts of variations in intervention uptake rate, screening efficacy and utility decrements on ICERs. |
| Frick (2010) | CD adults aged 65+ | CUA; US healthcare payer;^4^ 1 year^5^ | Exercise (2 forms); HAM; MF int. (2 forms); Vit. D; Med. modification [Cross-comparison] | ***Ratio***: HAM and vitamin D were the only non-dominated intervention; HAM had ICER of US$19,123 per QALY relative to vitamin D  ***Aggregate***: Not reported  ***Parameter uncertainty***: At threshold of US$64,763 per QALY, HAM had the highest INMB in 54.1% of replications, and vitamin D in 29.7%.  ***Scenarios***: No analysis |
| Hektoen (2009) | CD women aged 80+ | CEA; Societal; 1 year | Exercise [NR] | ***Ratio***: Home-based Otago exercise dominated no intervention.  ***Aggregate***: Not reported  ***Parameter uncertainty***: No analysis  ***Scenarios***: Intervention would break even if risk of injurious fall is reduced from 0.5 to 0.34 and if efficacy reduced to 22% from 40%. |
| Hiligsmann (2014) | Adults aged 60+ with osteoporosis | CUA; Societal; Lifetime | Vit. D and calcium [NR] | ***Ratio***: ICER per QALY for intervention vs. no intervention – US$62,084 and US$35,920 for women and men aged 60; US$12,105 and US$15,683 for women and men aged 70; intervention dominated no intervention for men and women aged 80.  ***Aggregate***: Not reported  ***Parameter uncertainty***: DSA – fracture risk and intervention cost had the largest impact on ICERs. PSA – CEAC; at threshold of US$68,849 per QALY, 49%, 87% and 99% probability of intervention being cost-effective for women aged 60, 70 and 80; 80%, 94% and 99% for men.  ***Scenarios***: Mortality risk reduction due to intervention, varying sustainability duration and varying discount rate had modest impact on ICER. |
| Hirst (2016) | Women aged 75+ on chronic pain medication | CUA; Public sector; 1 year | Med. modification (Transdermal Buprenorphine) [Tramadol] | ***Ratio***: transdermal buprenorphine had ICER of US$11,130 per QALY vs. tramadol  ***Aggregate***: Total cost and health gain per 100,000 women reported.  ***Parameter uncertainty***: DSA – Efficacy had the largest impact on ICER. PSA – CEAC; transdermal buprenorphine had 52% probability of being cost-effective vs. tramadol at threshold of US$31,895 per QALY  ***Scenarios***: Alternative efficacy source, lower adherence rate and targeting at age 85+ made transdermal buprenorphine dominate tramadol. |
| Howland (2015) | CD adults aged 65+ admitted to A&E due to fall | ROI; US healthcare payer;^4^ 1 year | MC int. (MoB/VLL) [NR] | ***Ratio***: ROI for intervention was 1.44:1.  ***Aggregate***: Total HC savings was US$6.3 million for state population at 50% uptake rate; US$3.2 million at 25% uptake and US$9.5 million at 75% uptake.  ***Parameter uncertainty***: No analysis  ***Scenarios***: See *Aggregate* for state-level scale up and impacts of varying uptake rate. |
| Ippoliti (2018) | CD adults aged 65+ living in mountainous areas | ROI; Public sector; 3 years | MF int. [NR] | ***Ratio***: For population of 191,977 over 3-year horizon, 1,657 hip fractures must be prevented for intervention to break even; this requires efficacy of 36% reduction in hip fracture.  ***Aggregate***: Reports number of hip fractures prevented.  ***Parameter uncertainty***: No analysis  ***Scenarios***: See *Ratio* for efficacy break-even analysis. |
| Lee (2013) | CD adults aged 65-80 without falls history | CBA; Public sector; 3 years | Vit. D (targeted, universal) [NR] | ***Ratio***: Targeted supplementation had average INMB of US$267 vs. no intervention for women and US$356 for men; universal supplementation had US$226 and US$310 respectively.  ***Aggregate***: Not reported  ***Parameter uncertainty***: DSA – efficacy and screening cost had the largest impact on INMBs. PSA – at threshold of US$59,545 per QALY, targeted supplementation was the most cost-effective option in 52.8% of the simulations for women and 54.3% for men, compared to 36.3% and 38.2% for universal supplementation.  ***Scenarios***: No analysis |
| Ling (2008) | CD adults aged 65+ with falls history or other risk factors | ROI; US healthcare payer;^4^ 1 year | HAM [NR] | ***Ratio***: HAM had ROI of 3.2:1 vs. no intervention.  ***Aggregate***: Not reported  ***Parameter uncertainty***: No analysis  ***Scenarios***: No analysis |
| McLean (2015) | CD adults aged 70+ | CEA, CUA; Public sector; 18 months | Exercise [UC] | ***Ratio***: (CUA results) Exercise had ICER of US$90,013 per QALY vs. usual care for whole sample and US$40,189 for women subgroup.  ***Aggregate***: Incremental cost and health gains reported.  ***Parameter uncertainty***: PSA – CEAC; 8.08% probability of exercise being cost-effective at US$52,464 per QALY threshold (76.77% for women subgroup)  ***Scenarios***: ICER reduced to US$44,865 per QALY if venue cost excluded and cheaper fitness instructors used (US$18,845 for women subgroup). |
| Miller (2011) | CD adults aged 50+ at high falls risk | ROI; US healthcare,^4^ Societal; 2 years | MC int. (MoB/VLL) [NR] | ***Ratio***: For high-risk subgroup, ROI above one is achieved if intervention averts 7.1 falls among 140 participants within the first year from societal perspective and 4.4 falls from healthcare perspective. For whole group, the numbers are 10.7 and 6.6 falls.  ***Aggregate***: Not reported  ***Parameter uncertainty***: No analysis  ***Scenarios***: Efficacy break-even analysis (see *Ratio*) |
| Mori (2017) | CD women aged 65+ at osteoporosis risk without previous fracture | CUA; Societal; Lifetime | Exercise and bisphosphonate combined [Cross-comparison: single or no intervention] | ***Ratio***: Combined therapy vs. bisphosphonate alone produced ICER per QALY of US$225,218 for age 65, US$132,063 for age 70, US$52,251 for age 75 and US$19,666 for age 80.  ***Aggregate***: Reports incremental cost and QALY which can be scaled up by age group.  ***Parameter uncertainty***: DSA – Efficacy and intervention cost significantly affected outcomes. PSA – At US$111,479 per QALY threshold, probabilities of combined therapy being cost-effective relative to next best alternative were 35% for baseline age 65, 40% for age 70, 42% for age 75 and 48% for age 80.  ***Scenarios***: Targeting exercise at osteoporosis patient subgroups can significantly improve the cost-effectiveness of combined therapy vs. bisphosphonate alone for all ages. |
| Moriarty (2019) | CD adults aged 65, no current/previous adverse events for benzodiazepine/PPI | CUA; Public sector; 35 years | Med. modification (Benzodiazepine, PPI) [Inappropriate prescribing] | ***Ratio***: No sedative use dominated inappropriate benzodiazepine use; Maintenance dose of PPI use dominated maximal dose use.  ***Aggregate***: Reports prevalence rates for each inappropriate medication use – 4.3% for benzodiazepine and 23.6% for PPI.  ***Parameter uncertainty***: DSA – see Table 3 in Moriarty (2019) for impacts of variations in inpatient cost of c. difficile infection (for PPI) and in costs of medications. PSA – scatter plot.  ***Scenarios***: See Table 3 in Moriarty (3) for impacts of variations in discount rate and adherence rate, and Table 4 for results of threshold analysis using medication costs. |
| Poole (2014) | Adults aged 65+ | ROI; Public sector; 1 year | Vit. D [NR] | ***Ratio***: Intervention generated net savings relative to no intervention.  ***Aggregate***: Total national net savings of US$36.7 million and 1,692 hip fractures prevented.  ***Parameter uncertainty***: No analysis  ***Scenarios***: No analysis (except age-based targeting) |
| Poole (2015) | CD adults aged 60+ | CUA, ROI; Public sector; 5 years | Vit. D [NR] | ***Ratio***: (CUA results) Intervention produced ICER of US$30,775 per QALY relative to no intervention for age 60+ (excluding LTC cost).  ***Aggregate***: Including LTC cost savings, intervention produced net cost savings of US$654.2 million for age 60+.  ***Parameter uncertainty***: No analysis  ***Scenarios***: No analysis (except age-based targeting) |
| PHE (2018) | CD adults aged 65+ | CUA, ROI; Public sector; 2 years | Exercise (3 forms); HAM [NR] | ***Ratio***: (CUA results) ICER per QALY were US$3,716 for Otago exercise, US$733 for FaME and US$12,161 for Tai Chi; HAM dominated no intervention. (ROI results) ROI of 0.95:1 for Otago, 0.99:1 for FaME, 0.85:1 for Tai Chi and 3.17:1 for HAM.  ***Aggregate***: Not reported  ***Parameter uncertainty***: DSA – discharge destination after hospitalised fall, utility decrement and HC cost of fall had little impact on results. No PSA.  ***Scenarios***: Tables 31-33 in PHE (2018) report changes to falls rate, MA falls risk and efficacy required to change decision for each intervention. |
| Sach (2007) | Women aged 70+ with bilateral cataracts | CEA, CUA; Public sector, Societal; Lifetime extrapol.^6^ | Exp. cataract surgery (first eye) [UC: Routine surgery] | ***Ratio***: ICER of US$26,412 per QALY under public sector perspective and US$20,817 under societal.  ***Aggregate***: Not reported  ***Parameter uncertainty***: PSA – CEAC; intervention had 90.4% probability of being cost-effective relative to routine surgery at US$41,900 (£30,000) per QALY threshold.  ***Scenarios***: ICER of US$3,490 per QALY if 5% high-cost outliers removed from sample; Significant impacts from changes to perspective and time horizon (e.g., ICER of US$71,616 per QALY under public sector perspective and 1-year horizon). |
| Sach (2010) | Women aged 70+ with second operable cataract | CUA; Public sector, Societal; Lifetime extrapol.^6^ | Exp. cataract surgery (second eye) [UC: No surgery] | ***Ratio***: ICER of US$34,687 per QALY under public sector perspective and US$84,161 under societal.  ***Aggregate***: Not reported  ***Parameter uncertainty***: PSA – CEAC  ***Scenarios***: Significant impacts from changes to perspective and time horizon (e.g., ICER of US$88,785 per QALY under public sector perspective and 1-year horizon). |
| Smith (2016) | Adults aged 65+ covered by GP practice and hospital | ROI; Public sector; 1 year | FRS + MF int. [Cross-comparison] | ***Ratio***: The falls risk cut-off where savings outweigh costs was P=0.27 which would result in 1.8% of the population being referred to intervention.  ***Aggregate***: Reports proportion of the population accessing intervention. The cut-off level which maximised total savings was P=0.53 (0.45% of population referred); the cut-off with maximum sensitivity (81%) and specificity (78%) was P=0.07.  ***Parameter uncertainty***: Reports 95% uncertainty intervals around estimates of net economic saving by cut-off level.  ***Scenarios***: Variation in falls risk cut-off levels (see *Ratio* and *Aggregate*) |
| Tannenbaum (2015) | CD adults aged 65+ with insomnia | CUA; Public sector; 1 year | Med. modification; CBT [NR; Cross-comparison] | ***Ratio***: CBT dominated pharmacologic therapy and no intervention.  ***Aggregate***: Not reported  ***Parameter uncertainty***: DSA – falls risk significantly affected results: at low risk, pharmacologic therapy dominated CBT and no intervention. PSA – CEAC.  ***Scenarios***: CBT remained dominant under 5-year horizon |
| Turner (2020) | CD adults aged 65+ who are chronic users of sedatives for insomnia | CUA; Public sector; 1 year | Med. modification [NR] | ***Ratio***: Intervention dominated no intervention; average INMB of US$4,543 at US$43,380 per QALY threshold and US$7,880 at US$86,758 threshold.  ***Aggregate***: Not reported  ***Parameter uncertainty***: PSA – CEAC, 100% probability of intervention being cost-effective relative to no intervention at US$43,380 and US$86,758 per QALY thresholds.  ***Scenarios***: Intervention dominance did not change for any scenarios. |
| Velde (2008) | CD geriatric outpatient population with falls history (mean age 78) | CEA; Public sector; 1 year^5^ | Med. modification [NR] | ***Ratio***: Intervention dominated no intervention; average net saving of US$2,367 per recipient.  ***Aggregate***: Scaling up intervention to national level for older persons with MA falls history (7% of population aged 65+) would generate total cost saving of US$84.1 million.  ***Parameter uncertainty***: PSA – 95% uncertainty interval reported.  ***Scenarios***: Intervention dominance did not change when falls risk reduced by 50%. |
| Wu (2010) | CD Medicare beneficiaries aged 65+ with falls history | CEA, ROI; Public sector, Societal; 1 year | MF int. [NR] | ***Ratio***: (CEA results) Intervention produced ICER of US$1,067 per MA fall prevented relative to no intervention under Medicare perspective and dominated no intervention under Medicare and private insurance perspective.  ***Aggregate***: Total net cost to Medicare was US$546 million for all age groups, US$352 million for age 65-74 and US$194 million for age 75+; Total net cost to all payers was US$997 million for all age groups, US$277 million for age 65-74 and US$721 million for age 75+.  ***Parameter uncertainty***: DSA – efficacy had the largest impact on ICER for CEA. No PSA.  ***Scenarios***: Proportion of HC cost of those with falls history that can be averted had the second largest impact on ICER for CEA. |
| **Abbreviation:** CBA: cost-benefit analysis; CBT: cognitive behavioural therapy; CD: community-dwelling; CEA: cost-effectiveness analysis; CEAC: cost-effectiveness acceptability curve; CEAF: cost-effectiveness acceptability frontier; CSP: Chartered Society of Physiotherapy; CUA: cost-utility analysis; DSA: deterministic sensitivity analysis; FaME: falls management exercise; HAM: home assessment and modification; HC: healthcare; ICER: incremental cost-effectiveness ratio; INMB: incremental net monetary benefit; LTC: long-term care; NR: non-receipt of modelled intervention(s); PHE: Public Health England; PPI: proton pump inhibitor; PSA: probabilistic sensitivity analysis; QALY: quality-adjusted life year; QTUG: quantified timed-up-and-go; ROI: return on investment; TUG: timed-up-and-go; UC: usual care  ^1^ See Table 2 in main manuscript text for study references; parenthesised number refers to the number of models included in the table.  ^2^ All monetary units are converted to US$ in year 2021 using the average consumer price index (CPI) between the original year of reported currency to 2019 (most recent year for CPI data) [83] in the country of study and purchasing power parity (PPP) rate between the original currency and US$ in year 2020 (most recent PPP data) [84].  ^3^ Intervention included individually tailored education, HAM and exercise and public space safety improvement.  ^4^ This would include Medicare/aid, private health insurance and patients.  ^5^ One-year horizon with lifetime costs and health effects of falls.  ^6^ One-year trial outcomes are extrapolated over lifetime horizon. | | | | |

# References

1. Davis J, Robertson MC, Comans T, Scuffham P. Guidelines for conducting and reporting economic evaluation of fall prevention strategies. Osteoporosis international. 2011;22(9):2449-59.

2. Lamb SE, Jørstad‐Stein EC, Hauer K, Becker C, Europe PoFN, Group OC. Development of a common outcome data set for fall injury prevention trials: the Prevention of Falls Network Europe consensus. Journal of the American Geriatrics Society. 2005;53(9):1618-22.

3. Drummond MF, Sculpher MJ, Claxton K, Stoddart GL, Torrance GW. Methods for the economic evaluation of health care programmes: Oxford university press; 2015.

4. Salomon JA, Vos T, Hogan DR, Gagnon M, Naghavi M, Mokdad A, et al. Common values in assessing health outcomes from disease and injury: disability weights measurement study for the Global Burden of Disease Study 2010. The Lancet. 2012;380(9859):2129-43.

5. Brazier J, Green C, Kanis J. A systematic review of health state utility values for osteoporosis-related conditions. Osteoporosis International. 2002;13(10):768-76.

6. Ström O, Borgström F, Sen S, Boonen S, Haentjens P, Johnell O, et al. Cost-effectiveness of alendronate in the treatment of postmenopausal women in 9 European countries-an economic evaluation based on the fracture intervention trial. Osteoporosis international. 2007;18(8):1047-61.

7. Honkanen LA, Mushlin AI, Lachs M, Schackman BR. Can Hip Protector Use Cost‐Effectively Prevent Fractures in Community‐Dwelling Geriatric Populations? Journal of the American Geriatrics Society. 2006;54(11):1658-65.

8. Iglesias CP, Manca A, Torgerson DJ. The health-related quality of life and cost implications of falls in elderly women. Osteoporosis international : a journal established as result of cooperation between the European Foundation for Osteoporosis and the National Osteoporosis Foundation of the USA. 2009;20(6):869-78. doi: 10.1007/s00198-008-0753-5. PubMed PMID: 18846400.

9. Salkeld G, Ameratunga SN, Cameron I, Cumming R, Easter S, Seymour J, et al. Quality of life related to fear of falling and hip fracture in older women: a time trade off study. BMJ. 2000;320(7231):341-6.

10. Peasgood T, Herrmann K, Kanis JA, Brazier JE. An updated systematic review of Health State Utility Values for osteoporosis related conditions. Osteoporosis international : a journal established as result of cooperation between the European Foundation for Osteoporosis and the National Osteoporosis Foundation of the USA. 2009;20(6):853-68. doi: 10.1007/s00198-009-0844-y. PubMed PMID: 19271098.

11. Eldridge S, Spencer A, Cryer C, Parsons S, Underwood M, Feder G. Why modelling a complex intervention is an important precursor to trial design: lessons from studying an intervention to reduce falls-related injuries in older people. Journal of health services research & policy. 2005;10(3):133-42.

12. Giles LC, Hawthorne G, Crotty M. Health-related quality of life among hospitalized older people awaiting residential aged care. Health and Quality of Life Outcomes. 2009;7(1):71.

13. Kind P, Hardman G, Macran S. UK population norms for EQ-5D. 1999.

14. Ara R, Brazier JE. Populating an economic model with health state utility values: moving toward better practice. Value in Health. 2010;13(5):509-18.

15. Thiem U, Klaaßen-Mielke R, Trampisch U, Moschny A, Pientka L, Hinrichs T. Falls and EQ-5D rated quality of life in community-dwelling seniors with concurrent chronic diseases: a cross-sectional study. Health and quality of life outcomes. 2014;12(1):2.

16. Poole CD, Smith J, Davies JS. Cost-effectiveness and budget impact of Empirical vitamin D therapy on unintentional falls in older adults in the UK. BMJ open. 2015;5(9):e007910. doi: <https://dx.doi.org/10.1136/bmjopen-2015-007910>.

17. Jonsson B, Kanis J, Dawson A, Oden A, Johnell O. Effect and offset of effect of treatments for hip fracture on health outcomes. Osteoporosis international. 1999;10(3):193-9.

18. Hiligsmann M, Ethgen O, Richy F, Reginster J-Y. Utility values associated with osteoporotic fracture: a systematic review of the literature. Calcified tissue international. 2008;82(4):288-92.

19. Kind P, Dolan P, Gudex C, Williams A. Variations in population health status: Results from a United Kingdom national questionnaire survey. British Medical Journal. 1998;316(7133):736-41.

20. Amgen UK Ltd. Single technology appraisal (STA) to NICE denosumab for the prevention of osteoporotic fractures in postmenopausal women. 2010.

21. Fryback DG, Dasbach EJ, Klein R, Klein BE, Dorn N, Peterson K, et al. The Beaver Dam Health Outcomes Study: initial catalog of health-state quality factors. Medical Decision Making. 1993;13(2):89-102.

22. Gabriel SE, Kneeland TS, Melton LJ, Moncur MM, Ettinger B, Tosteson AN. Health-related quality of life in economic evaluations for osteoporosis: whose values should we use? Medical Decision Making. 1999;19(2):141-8.

23. Neumann PJ, Hermann R, Kuntz K, Araki S, Duff S, Leon J, et al. Cost-effectiveness of donepezil in the treatment of mild or moderate Alzheimer’s disease. Neurology. 1999;52(6):1138-.

24. Segui-Gomez M, Keuffel E, Frick KD. Cost and effectiveness of hip protectors among the elderly. International journal of technology assessment in health care. 2002;18(1):55-66.

25. Burström K, Johannesson M, Diderichsen F. Health-related quality of life by disease and socio-economic group in the general population in Sweden. Health policy. 2001;55(1):51-69.

26. Borgström F, Zethraeus N, Johnell O, Lidgren L, Ponzer S, Svensson O, et al. Costs and quality of life associated with osteoporosis-related fractures in Sweden. Osteoporosis International. 2006;17(5):637-50.

27. Hanmer J, Lawrence WF, Anderson JP, Kaplan RM, Fryback DG. Report of nationally representative values for the noninstitutionalized US adult population for 7 health-related quality-of-life scores. Medical Decision Making. 2006;26(4):391-400.

28. Friedman SM, Munoz B, West SK, Rubin GS, Fried LP. Falls and fear of falling: which comes first? A longitudinal prediction model suggests strategies for primary and secondary prevention. J Am Geriatr Soc. 2002;50(8):1329-35. PubMed PMID: 12164987.

29. Murphy SL, Williams CS, Gill TM. Characteristics associated with fear of falling and activity restriction in community-living older persons. J Am Geriatr Soc. 2002;50(3):516-20. PubMed PMID: 11943049; PubMed Central PMCID: PMCPMC3046411.

30. National Osteoporosis Foundation. Osteoporosis: review of the evidence for prevention, diagnosis and treatment and cost-effective analysis. Osteoporosis International. 1998;8:S7-S80.

31. Si L, Winzenberg T, de Graaff B, Palmer A. A systematic review and meta-analysis of utility-based quality of life for osteoporosis-related conditions. Osteoporosis international. 2014;25(8):1987-97.

32. Si L, Winzenberg T, Jiang Q, Palmer A. Screening for and treatment of osteoporosis: construction and validation of a state-transition microsimulation cost-effectiveness model. Osteoporosis International. 2015;26(5):1477-89.

33. Church J, Goodall S, Norman R, Haas M. An economic evaluation of community and residential aged care falls prevention strategies in NSW. New South Wales public health bulletin. 2011;22(3-4):60-8. doi: <https://dx.doi.org/10.1071/NB10051>.

34. Cranney AB, Coyle D, Hopman WM, Hum V, Power B, Tugwell PS. Prospective evaluation of preferences and quality of life in women with hip fractures. The Journal of rheumatology. 2005;32(12):2393-9.

35. Cranney A, Coyle D, Pham B, Tetroe J, Wells G, Jolly E, et al. The psychometric properties of patient preferences in osteoporosis. The Journal of rheumatology. 2001;28(1):132-7.

36. Papaioannou A, Kennedy CC, Ioannidis G, Sawka A, Hopman WM, Pickard L, et al. The impact of incident fractures on health-related quality of life: 5 years of data from the Canadian Multicentre Osteoporosis Study. Osteoporosis International. 2009;20(5):703-14.

37. Couzner L, Crotty M, Norman R, Ratcliffe J. A comparison of the EQ-5D-3L and ICECAP-O in an older post-acute patient population relative to the general population. Applied health economics and health policy. 2013;11(4):415-25.

38. Church J, Goodall S, Norman R, Haas M. The cost-effectiveness of falls prevention interventions for older community-dwelling Australians. Aust N Z J Public Health. 2012;36(3):241-8. doi: 10.1111/j.1753-6405.2011.00811.x. PubMed PMID: 22672030.

39. Léger D, Morin CM, Uchiyama M, Hakimi Z, Cure S, Walsh JK. Chronic insomnia, quality-of-life, and utility scores: comparison with good sleepers in a cross-sectional international survey. Sleep medicine. 2012;13(1):43-51.

40. Borgström F, Sobocki P, Ström O, Jönsson B. The societal burden of osteoporosis in Sweden. Bone. 2007;40(6):1602-9.

41. Moriarty F, Cahir C, Bennett K, Fahey T. Economic impact of potentially inappropriate prescribing and related adverse events in older people: A cost-utility analysis using Markov models. BMJ Open. 2019;9(1):e021832. doi: <http://dx.doi.org/10.1136/bmjopen-2018-021832>.

42. Kanis J, Johnell O, Odén A, Borgstrom F, Zethraeus N, De Laet C, et al. The risk and burden of vertebral fractures in Sweden. Osteoporosis international. 2004;15(1):20-6.

43. Jönsson B, Christiansen C, Johnell O, Hedbrandt J. Cost-effectiveness of fracture prevention in established osteoporosis. Osteoporosis international. 1995;5(2):136-42.

44. Gillespie LD, Robertson MC, Gillespie WJ, Sherrington C, Gates S, Clemson LM, et al. Interventions for preventing falls in older people living in the community. Cochrane database of systematic reviews. 2012;(9).

45. Harwood RH, Foss A, Osborn F, Gregson R, Zaman A, Masud T. Falls and health status in elderly women following first eye cataract surgery: a randomised controlled trial. British Journal of Ophthalmology. 2005;89(1):53-9.

46. Campbell AJ, Robertson MC, Gardner MM, Norton RN, Buchner DM. Falls prevention over 2 years: a randomized controlled trial in women 80 years and older. Age and ageing. 1999;28(6):513-8.

47. Li F, Harmer P, Fisher KJ, McAuley E, Chaumeton N, Eckstrom E, et al. Tai Chi and fall reductions in older adults: a randomized controlled trial. The Journals of Gerontology Series A: Biological Sciences and Medical Sciences. 2005;60(2):187-94.

48. Clemson L, Cumming RG, Kendig H, Swann M, Heard R, Taylor K. The effectiveness of a community‐based program for reducing the incidence of falls in the elderly: A randomized trial. Journal of the American Geriatrics Society. 2004;52(9):1487-94.

49. Wolf SL, Barnhart HX, Kutner NG, McNeely E, Coogler C, Xu T, et al. Reducing frailty and falls in older persons: an investigation of Tai Chi and computerized balance training. Journal of the American Geriatrics Society. 1996;44(5):489-97.

50. Robertson MC, Gardner MM, Devlin N, McGee R, Campbell AJ. Effectiveness and economic evaluation of a nurse delivered home exercise programme to prevent falls. 2: Controlled trial in multiple centres. BMJ. 2001;322(7288):701-4. PubMed PMID: 11264207; PubMed Central PMCID: PMCPMC30095.

51. Cumming RG, Thomas M, Szonyi G, Salkeld G, O'neill E, Westbury C, et al. Home visits by an occupational therapist for assessment and modification of environmental hazards: a randomized trial of falls prevention. Journal of the American geriatrics society. 1999;47(12):1397-402.

52. Close J, Ellis M, Hooper R, Glucksman E, Jackson S, Swift C. Prevention of falls in the elderly trial (PROFET): a randomised controlled trial. Lancet. 1999;353(9147):93-7. doi: 10.1016/S0140-6736(98)06119-4. PubMed PMID: 10023893.

53. Campbell AJ, Robertson MC, Gardner MM, Norton RN, Buchner DM. Psychotropic medication withdrawal and a home‐based exercise program to prevent falls: a randomized, controlled trial. Journal of the American geriatrics society. 1999;47(7):850-3.

54. Kenny RAM, Richardson DA, Steen N, Bexton RS, Shaw FE, Bond J. Carotid sinus syndrome: a modifiable risk factor for nonaccidental falls in older adults (SAFE PACE). Journal of the American College of Cardiology. 2001;38(5):1491-6.

55. Barry E, Galvin R, Keogh C, Horgan F, Fahey T. Is the Timed Up and Go test a useful predictor of risk of falls in community dwelling older adults: a systematic review and meta-analysis. BMC geriatrics. 2014;14(1):14.

56. Greene BR, Redmond SJ, Caulfield B. Fall risk assessment through automatic combination of clinical fall risk factors and body-worn sensor data. IEEE journal of biomedical and health informatics. 2017;21(3):725-31.

57. Sherrington C, Fairhall NJ, Wallbank GK, Tiedemann A, Michaleff ZA, Howard K, et al. Exercise for preventing falls in older people living in the community. Cochrane database of systematic reviews. 2019;(1).

58. Robertson MC, Devlin N, Scuffham P, Gardner MM, Buchner DM, Campbell AJ. Economic evaluation of a community based exercise programme to prevent falls. J Epidemiol Community Health. 2001;55(8):600-6. PubMed PMID: 11449021; PubMed Central PMCID: PMCPMC1731948.

59. Boonen S, Lips P, Bouillon R, Bischoff-Ferrari HA, Vanderschueren D, Haentjens P. Need for additional calcium to reduce the risk of hip fracture with vitamin D supplementation: evidence from a comparative metaanalysis of randomized controlled trials. The Journal of Clinical Endocrinology & Metabolism. 2007;92(4):1415-23.

60. Tang BM, Eslick GD, Nowson C, Smith C, Bensoussan A. Use of calcium or calcium in combination with vitamin D supplementation to prevent fractures and bone loss in people aged 50 years and older: a meta-analysis. The Lancet. 2007;370(9588):657-66.

61. Bischoff-Ferrari HA, Willett WC, Wong JB, Stuck AE, Staehelin HB, Orav EJ, et al. Prevention of nonvertebral fractures with oral vitamin D and dose dependency: a meta-analysis of randomized controlled trials. Archives of internal medicine. 2009;169(6):551-61.

62. Li L, Setoguchi S, Cabral H, Jick S. Opioid Use for Noncancer Pain and Risk of Fracture in Adults: A Nested Case-Control Study Using the General Practice Research Database. American Journal of Epidemiology. 2013;178(4):559-69. doi: 10.1093/aje/kwt013.

63. Gallagher AM, Leighton-Scott J, van Staa TP. Utilization characteristics and treatment persistence in patients prescribed low-dose buprenorphine patches in primary care in the United Kingdom: a retrospective cohort study. Clinical therapeutics. 2009;31(8):1707-15.

64. Patel S, Ogunremi L, Chinappen U. Acceptability and compliance with hip protectors in community‐dwelling women at high risk of hip fracture. Rheumatology. 2003;42(6):769-72.

65. Kannus P, Parkkari J, Niemi S, Pasanen M, Palvanen M, Järvinen M, et al. Prevention of hip fracture in elderly people with use of a hip protector. New England journal of medicine. 2000;343(21):1506-13.

66. Woo J, Sum C, Yiu H, Ip K, Chung L, Ho L. Efficacy of a specially designed hip protector for hip fracture prevention and compliance with use in elderly Hong Kong Chinese. Clinical rehabilitation. 2003;17(2):203-5.

67. Zijlstra GR, Van Haastregt JC, Ambergen T, Van Rossum E, Van Eijk JTM, Tennstedt SL, et al. Effects of a multicomponent cognitive behavioral group intervention on fear of falling and activity avoidance in community‐dwelling older adults: Results of a randomized controlled trial. Journal of the American Geriatrics Society. 2009;57(11):2020-8.

68. Kalyani RR, Stein B, Valiyil R, Manno R, Maynard JW, Crews DC. Vitamin D treatment for the prevention of falls in older adults: systematic review and meta‐analysis. Journal of the American Geriatrics Society. 2010;58(7):1299-310.

69. Murad MH, Elamin KB, Abu Elnour NO, Elamin MB, Alkatib AA, Fatourechi MM, et al. The effect of vitamin D on falls: a systematic review and meta-analysis. The Journal of Clinical Endocrinology & Metabolism. 2011;96(10):2997-3006.

70. Fitzharris MP, Day L, Lord SR, Gordon I, Fildes B. The Whitehorse NoFalls trial: effects on fall rates and injurious fall rates. Age and ageing. 2010;39(6):728-33.

71. Robertson MC, Campbell AJ, Gardner MM, Devlin N. Preventing injuries in older people by preventing falls: A meta‐analysis of individual‐level data. Journal of the American geriatrics society. 2002;50(5):905-11.

72. Tannenbaum C, Martin P, Tamblyn R, Benedetti A, Ahmed S. Reduction of inappropriate benzodiazepine prescriptions among older adults through direct patient education: the EMPOWER cluster randomized trial. JAMA internal medicine. 2014;174(6):890-8.

73. Clyne B, Smith SM, Hughes CM, Boland F, Bradley MC, Cooper JA, et al. Effectiveness of a multifaceted intervention for potentially inappropriate prescribing in older patients in primary care: a cluster-randomized controlled trial (OPTI-SCRIPT Study). The Annals of Family Medicine. 2015;13(6):545-53.

74. Moayyeri A. The association between physical activity and osteoporotic fractures: a review of the evidence and implications for future research. Annals of epidemiology. 2008;18(11):827-35.

75. Bischoff-Ferrari HA, Willett WC, Orav EJ, Lips P, Meunier PJ, Lyons RA, et al. A pooled analysis of vitamin D dose requirements for fracture prevention. New England Journal of Medicine. 2012;367(1):40-9.

76. Bischoff-Ferrari HA, Dawson-Hughes B, Staehelin HB, Orav JE, Stuck A, Theiler R, et al. Fall prevention with supplemental and active forms of vitamin D: a meta-analysis of randomised controlled trials. Bmj. 2009;339:b3692.

77. Iliffe S, Kendrick D, Morris R, Masud T, Gage H, Skelton D, et al. Multicentre cluster randomised trial comparing a community group exercise programme and home-based exercise with usual care for people aged 65 years and over in primary care. Health technology assessment (Winchester, England). 2014;18(49):vii-105. doi: <https://dx.doi.org/10.3310/hta18490>.

78. Woolcott JC, Richardson KJ, Wiens MO, Patel B, Marin J, Khan KM, et al. Meta-analysis of the impact of 9 medication classes on falls in elderly persons. Archives of internal medicine. 2009;169(21):1952-60.

79. Stone KL, Ancoli-Israel S, Blackwell T, Ensrud KE, Cauley JA, Redline S, et al. Actigraphy-measured sleep characteristics and risk of falls in older women. Archives of internal medicine. 2008;168(16):1768-75.

80. Martin P, Tamblyn R, Benedetti A, Ahmed S, Tannenbaum C. Effect of a pharmacist-led educational intervention on inappropriate medication prescriptions in older adults: the D-PRESCRIBE randomized clinical trial. Jama. 2018;320(18):1889-98.

81. Chang JT, Morton SC, Rubenstein LZ, Mojica WA, Maglione M, Suttorp MJ, et al. Interventions for the prevention of falls in older adults: systematic review and meta-analysis of randomised clinical trials. Bmj. 2004;328(7441):680.

82. Heaney RP, Davies KM, Chen TC, Holick MF, Barger-Lux MJ. Human serum 25-hydroxycholecalciferol response to extended oral dosing with cholecalciferol. The American journal of clinical nutrition. 2003;77(1):204-10.

83. World Bank. Inflation, consumer prices (annual %) 2021. Available from: <https://data.worldbank.org/indicator/FP.CPI.TOTL.ZG>.

84. OECD Data. Purchasing power parities (PPP) 2021. Available from: <https://data.oecd.org/conversion/purchasing-power-parities-ppp.htm#indicator-chart>.
